# Supplementary material for: Global patterns in functional rarity of marine fish
Source: Nat Commun. 2022 Feb 15;13:877. doi: 10.1038/s41467-022-28488-1 (PMC8847455; doi:10.1038/s41467-022-28488-1)
Supplement: Supplementary file 1 — Supplementary Information [file 41467_2022_28488_MOESM1_ESM.pdf]

Supplementary Information

This document contains all Supplementary Information for the following article:

"Global patterns in functional rarity of marine fish"

Authors:

Isaac Trinidad-Santos[1*], Faye Moyes[1], Anne E. Magurran[1]

Author Contacts: its5@st-andrews.ac.uk, fhm1@st-andrews.ac.uk, aem1@st-andrews.ac.uk

[1] Centre for Biological Diversity, School of Biology, University of St Andrews, St Andrews KY16 9TH, Scotland, United Kingdom.

[*]Corresponding author

ORCID IDs

Isaac Trindade-Santos - https://orcid.org/0000-0003-4478-8103

Faye Moyes - https://orcid.org/0000-0001-9687-0593

Anne E. Magurran - https://orcid.org/0000-0002-0036-2795

Data Availability

The datasets generated during and/or analysed during the current study are available in the Research Portal of the University of St Andrews repository, https://doi.org/10.17630/397bc872-f7de-4ded-9ed8-4f734c11b14a [1].

Cite data as: Trindade-Santos, I., Moyes, F. & Magurran, A. Data Underpinning Global patterns in functional rarity of marine fish. University of St Andrews, doi:https://doi.org/10.17630/397bc872-f7de-4ded-9ed8-4f734c11b14a (2022).

Supplementary Figures

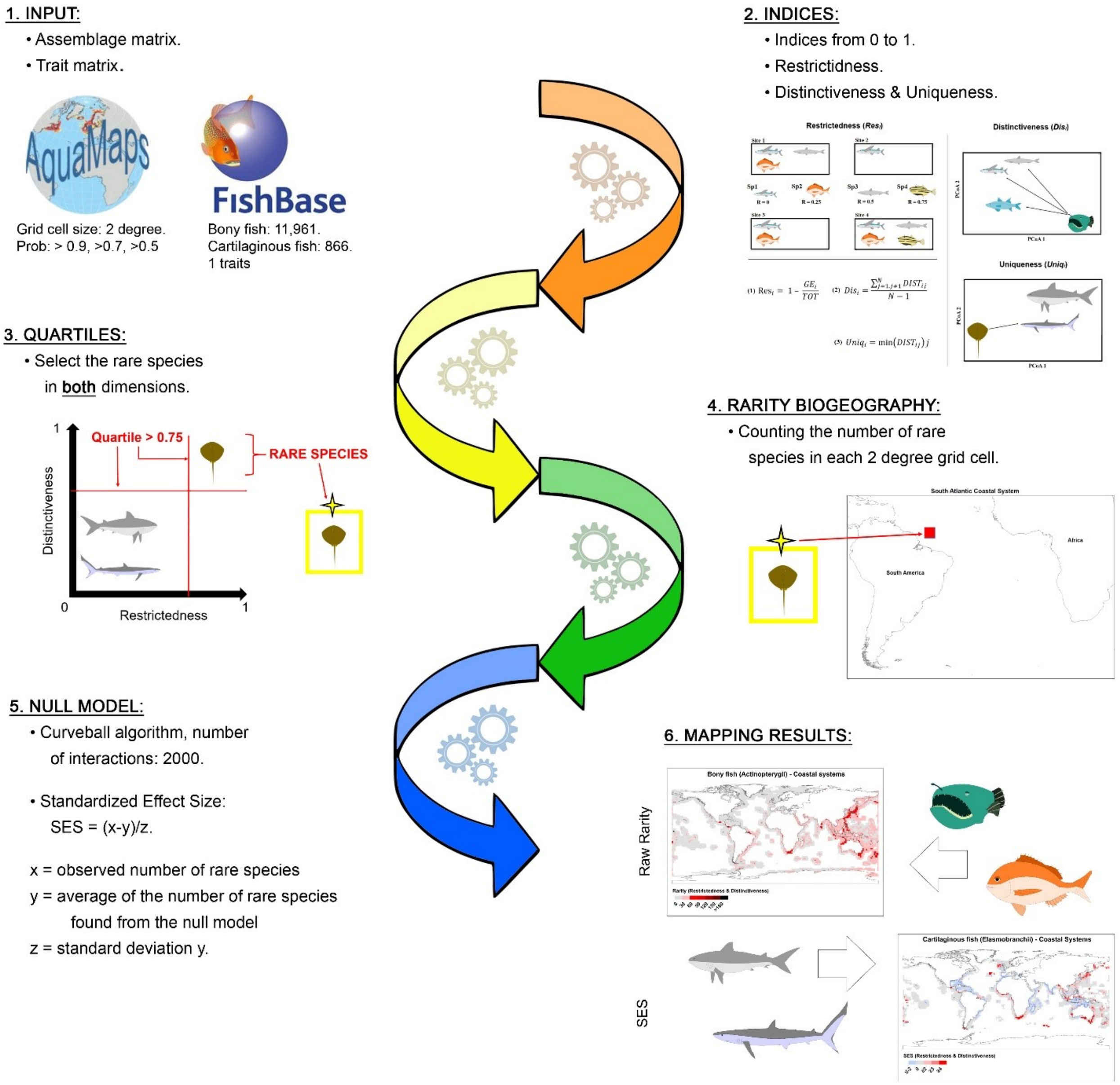

Supplementary Figure 1 Flow chart describing the steps at each stage of our analyses. These are expanded on in detail in the Methods section. The analyses were done individually for each of the following: 7 Coastal Systems and 7 High Seas (Supplementary Figure 3), Actinopterygii and Elasmobranchii, functional distinctiveness and uniqueness, probabilities of occurrences > 0.9, > 0.7 and > 0.5.

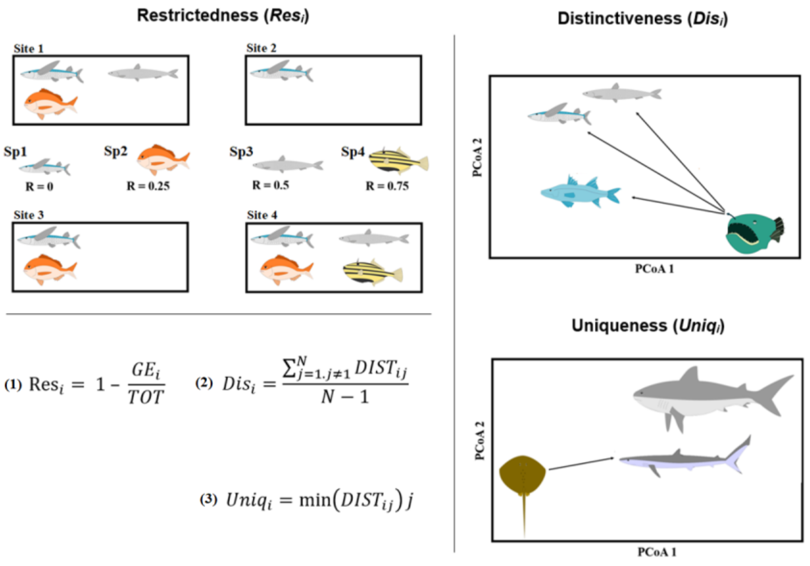

Supplementary Figure 2 | Schematic representation and equations of the rarity metrics used. Taxonomic restrictedness, functional distinctiveness, together with the alternative metric – functional uniqueness. Using the schematic representation we have as an example Sp1 occurring in all sites and Sp4 at only one site (restrictedness 0 and 0.75 respectively). The combination of traits of each species determines its position inside of the functional space, here represented by two PCoA1 and PCoA2 axes. Functional distinctiveness is measured by the average of the functional distance of a given species compared with all other species in the regional pool. In this example, the angler fish will have the highest functional distinctiveness because its combination of traits places it at a greater distance from the other 3 species. Functional uniqueness quantifies the distance in functional space between a given species to its nearest neighbour. In this schematic example we see that the nearest species to the ray is the blue shark, while the blue shark and the white shark are closer to each other, As such the ray will have the highest uniqueness in this assemblage. **Res*ᵢ*** - GE is geographic extent or number of grid cells a species occurs in, TOT is total number of grid cells and *i* is a species. **Dis*ᵢ*** - where N is the number of species, *i* and *j* are species and $DIST_{ij}$ is the distance between species *i* and *j*. **Uniq*ᵢ*** - where *i* and *j* are species and $DIST_{ij}$ is the distance between them.

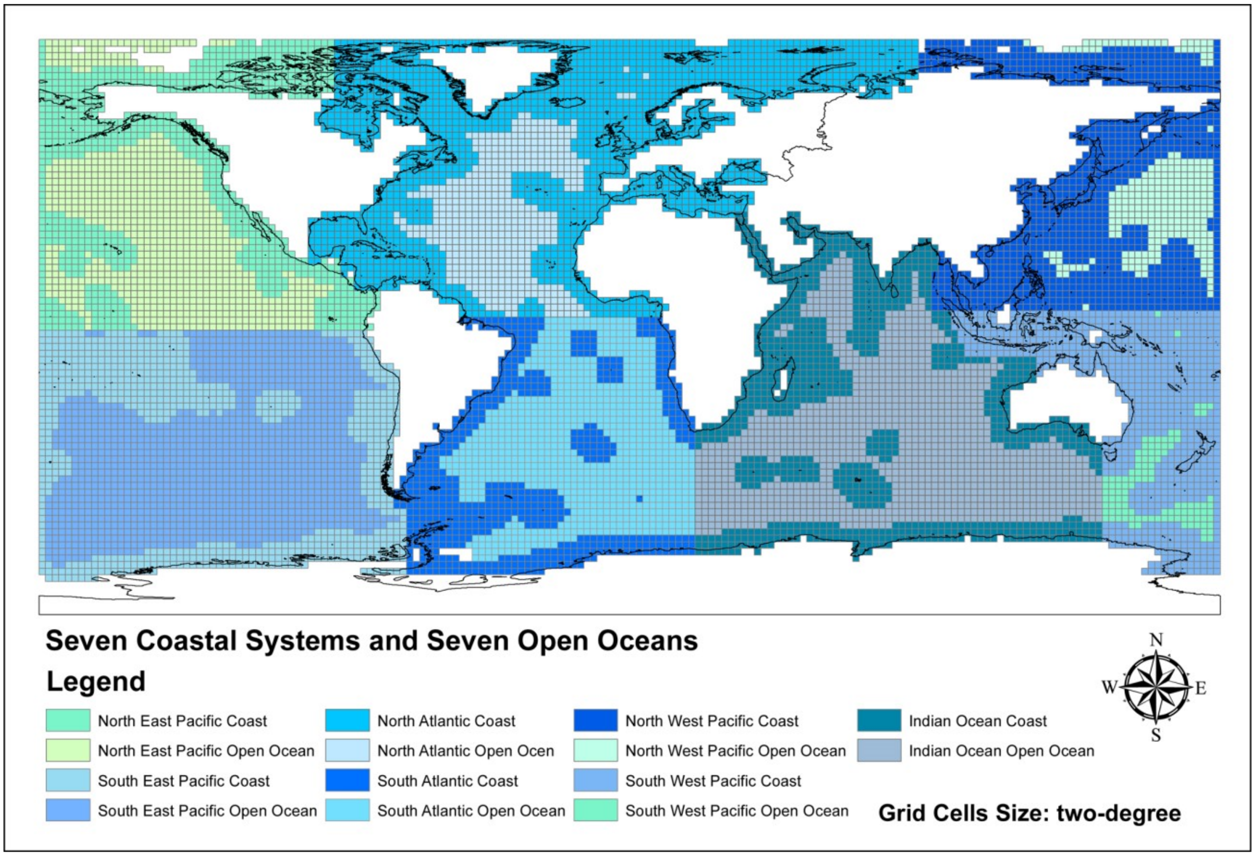

Supplementary Figure 3 | All systems used in our study, from the Seven Oceanic regions and its Coastal Systems and High Seas. All analyses were undertaken separately for bony and cartilaginous fish. The grid cells illustrated here are 2 degrees.

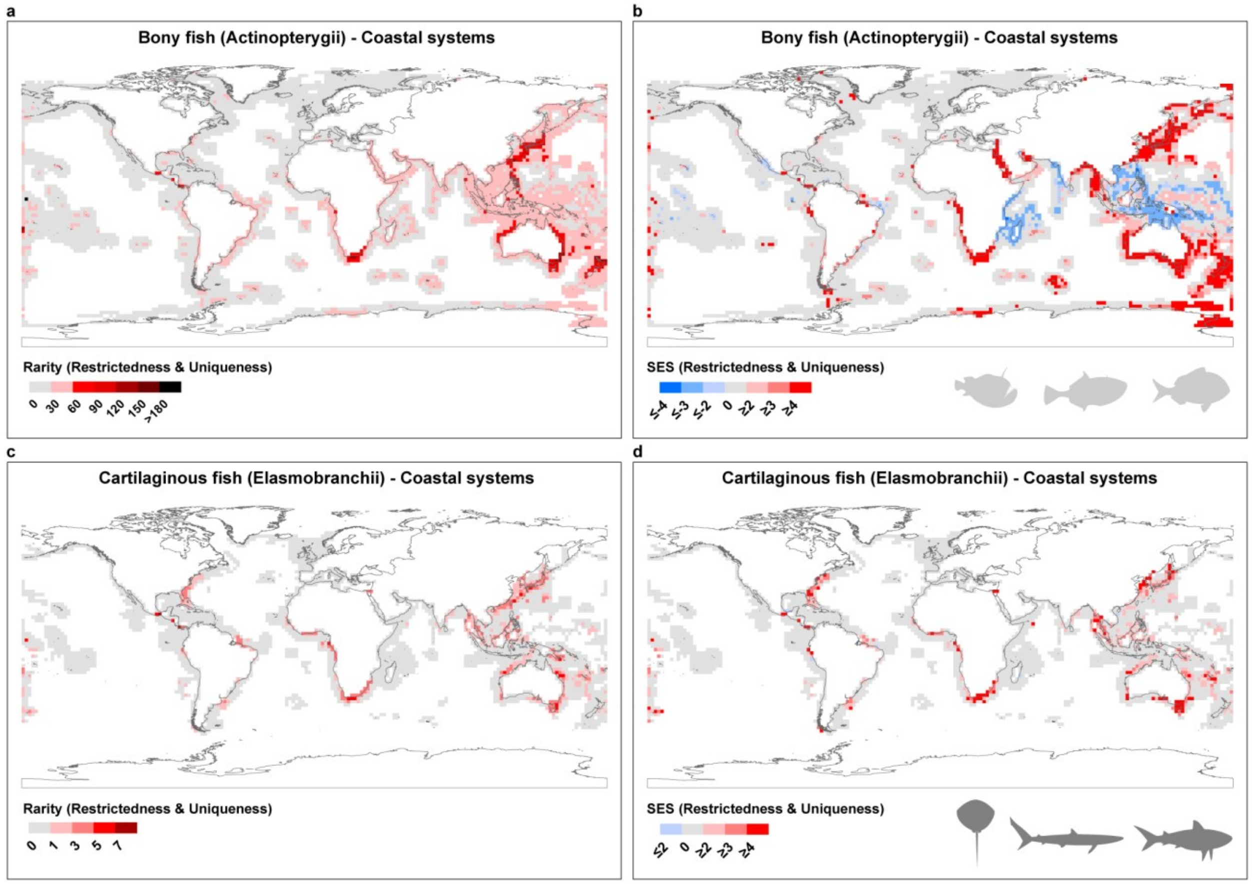

Supplementary Figure 4 | Global biogeography of rarity for bony fishes (a - Actinopterygii) and cartilaginous fishes (c - Elasmobranchii) across Coastal Systems. The functional index used here was **uniqueness** (see Supplementary Figure 1 for workflow and Supplementary Figure 2 for index details). Plots a and c illustrate the numbers of rare species found in each 2º grid cell (species that are rare **taxonomically** and **functionally (unique)** at the same time). Plots b and d show the Standardized Effect Sizes (SES) distribution, where red shaded cells represent an excess of rare species higher than expected by chance and blue cells represent fewer rare species than expected.

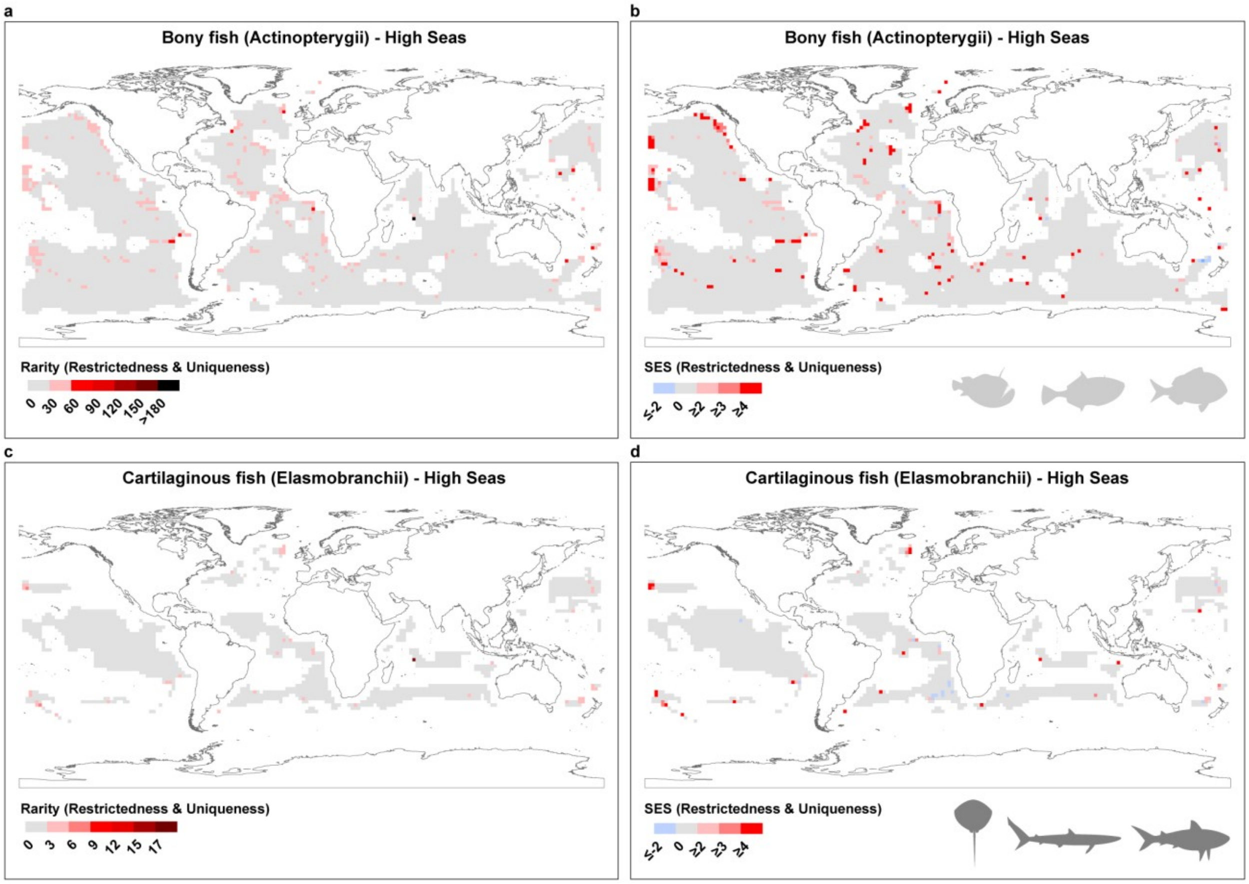

Supplementary Figure 5 | Global biogeography of rarity for bony fishes (a - Actinopterygii) and cartilaginous fishes (c - Elasmobranchii) across High Seas. The functional index used here was **uniqueness** (as shown in Supplementary Figure 2).

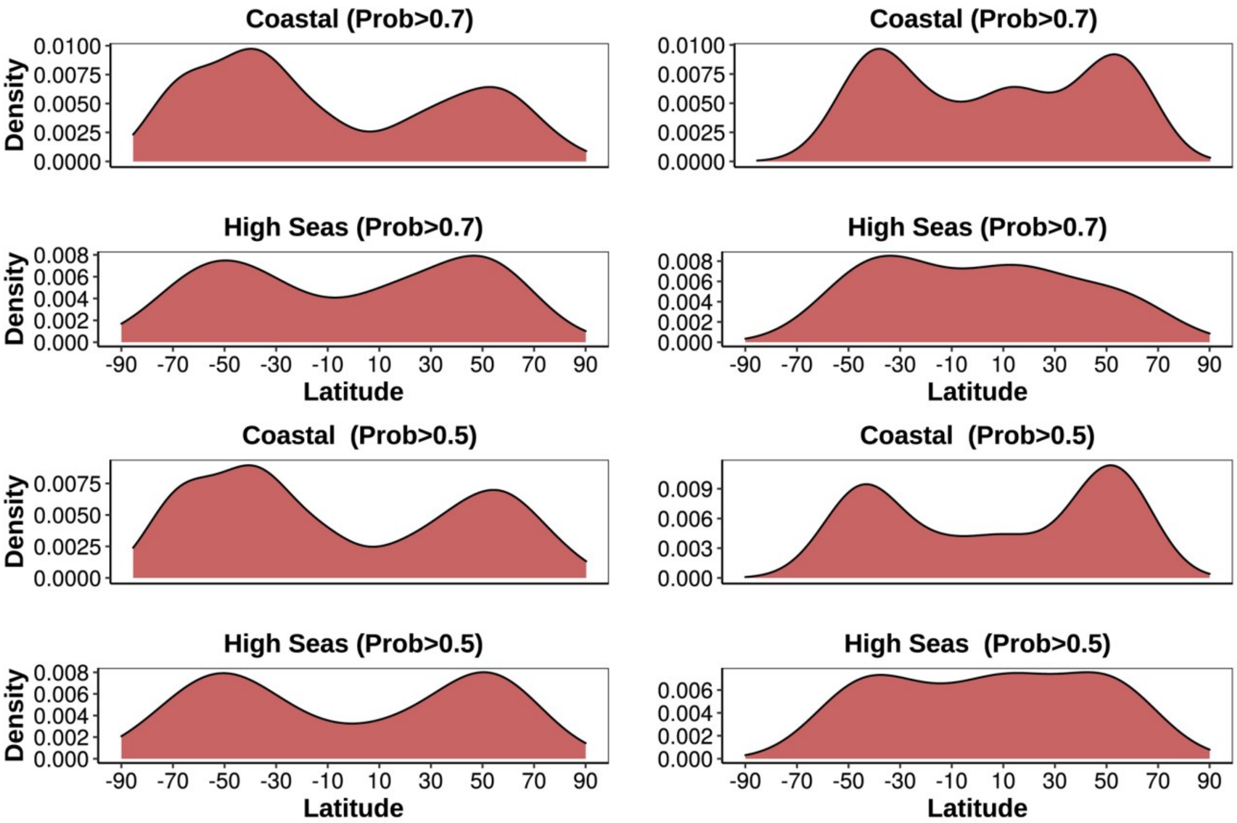

Supplementary Figure 6 | Latitudinal biogeographic distribution of the positive Standardized Effect Sizes (SES), found using two alternative probabilities of occurrances provided by the AquaMaps database, those were: Prob>0.7 (from a to d) and Prob>0.5 (from e to h). These results are for the Coastal Systems (a and b, e and f) and High Seas (c and d, g and h).

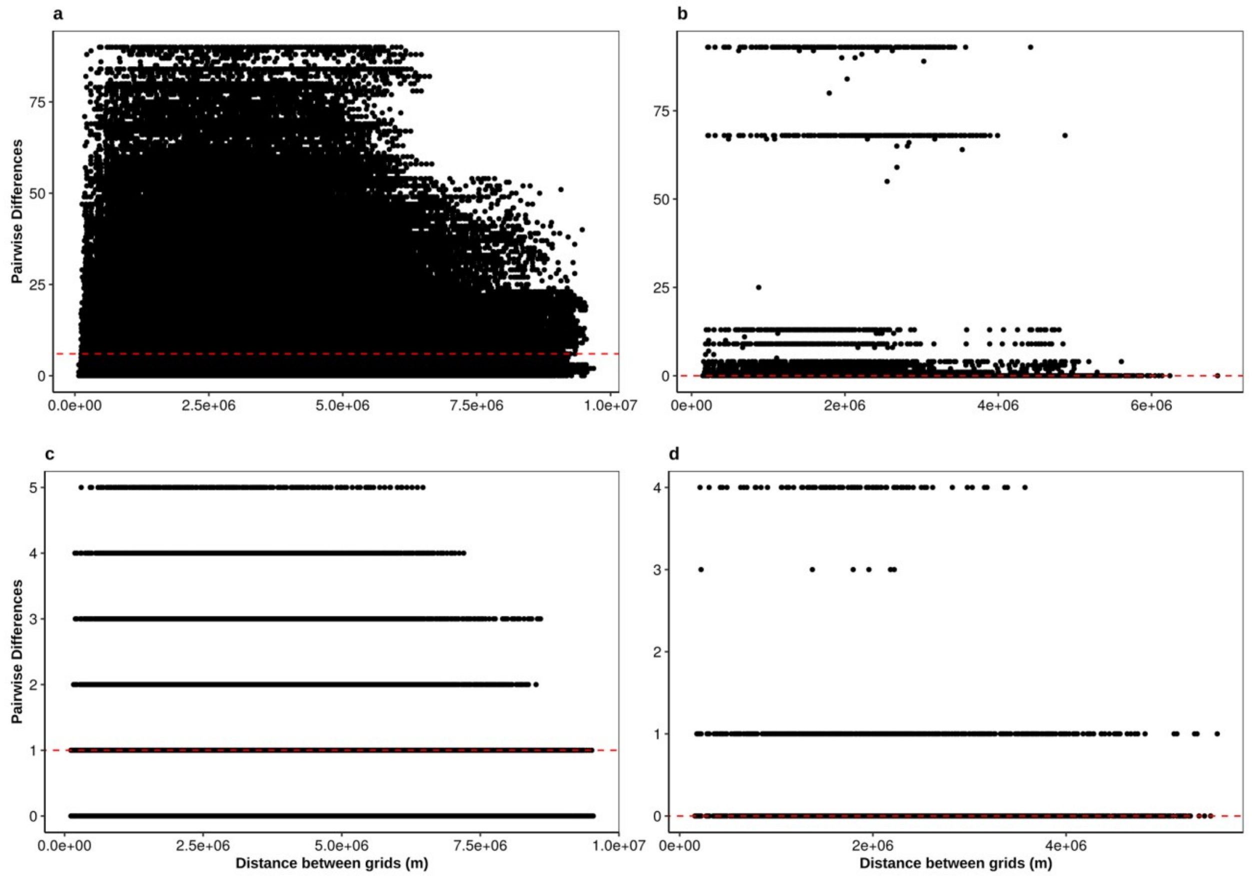

Supplementary Figure 7 | Distance decay plots for the Northwest Pacific Ocean. a and b represent the Coastal System and High Seas from the Northwest Pacific Ocean respectively, both for bony fish. For c and d we have Coastal Systems and High Seas, both for cartilaginous fish. The red dashed line represents the quantile regression.

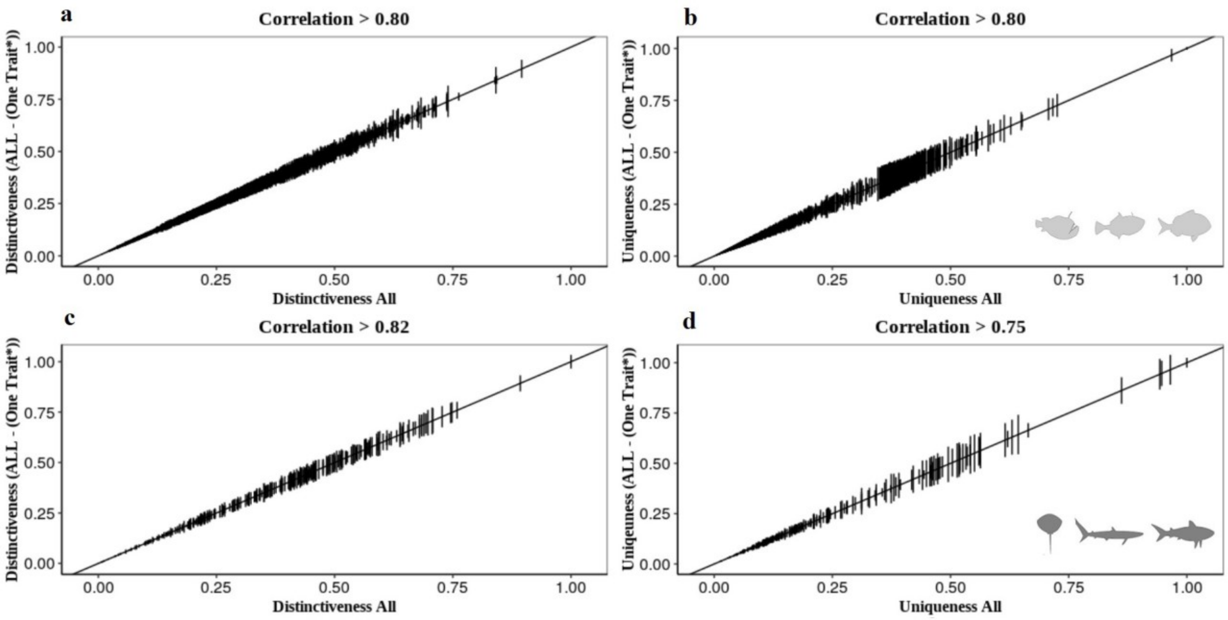

Sensitivity analysis results of the Spearman correlations between indices computed with all 11 traits - One Trait.

	Bony Fish		Cartilaginous Fish	
List of Correlations Made	Distinctivenness	Uniqueness	Distinctivenness	Uniqueness
All - Depth Max	0.98123	0.96198	0.95777	0.92122
All - TemPrefMean	0.90156	0.90691	0.97825	0.91983
All - (Depth Max & TemPrefMean)	0.79537	0.80303	0.89623	0.83049
All - RepGuildCombined	0.82385	0.93699	0.82407	0.93727
All - BodyShapeIII_combined	0.97034	0.84637	0.94303	0.84021
All - PositionWaterColumn	0.87066	0.89407	0.96878	0.75413
All - SwimMode	0.96507	0.94885	0.95196	0.97209

Supplementary Figure 8 | Sensitivity analysis for bony fish a (distinctiveness) and b (uniqueness), cartilaginous fish c (distinctiveness) and d (uniqueness); a visualisation and analysis using the Coastal System of the Northwest Pacific Ocean as an example. The plots illustrate the relationship between the estimates of rarity when each trait (*environmental traits: temperature and depth, and the categorical traits: reproductive guild, body shape, position in water column and swimming mode) is omitted in turn, and the value obtained with all eleven traits combined**.** The correlation shown above each of those was calculated between the indices found with all traits and the indices found without each of those traits. The table below the plot shows all the sensitivity analysis results found from the Spearman correlations (indices found from all traits by indices found from all traits minus one trait).

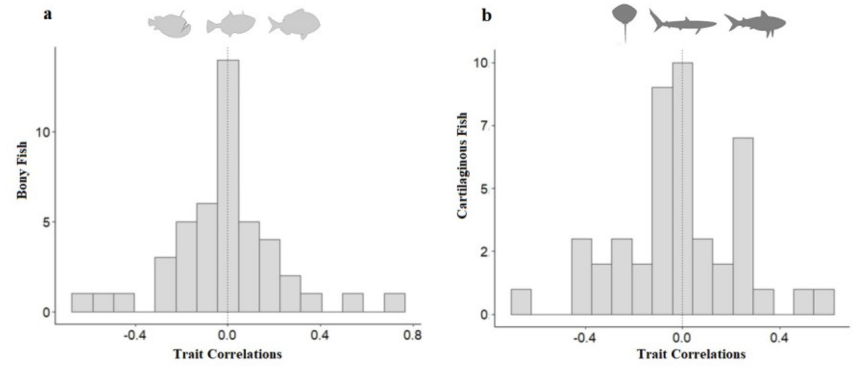

Supplementary Figure 9 | Distribution of correlations between trait values for the Actinopterygii (a) and Elasmobrachii (b). These are weak and largely centered on zero, particularly for the Actinopterygii, indicating that traits provide complementary information on function. This figure shows the a priori correlation test between all 11 traits, performed to clarify that the traits selected in all analyses are largely uncorrelated.

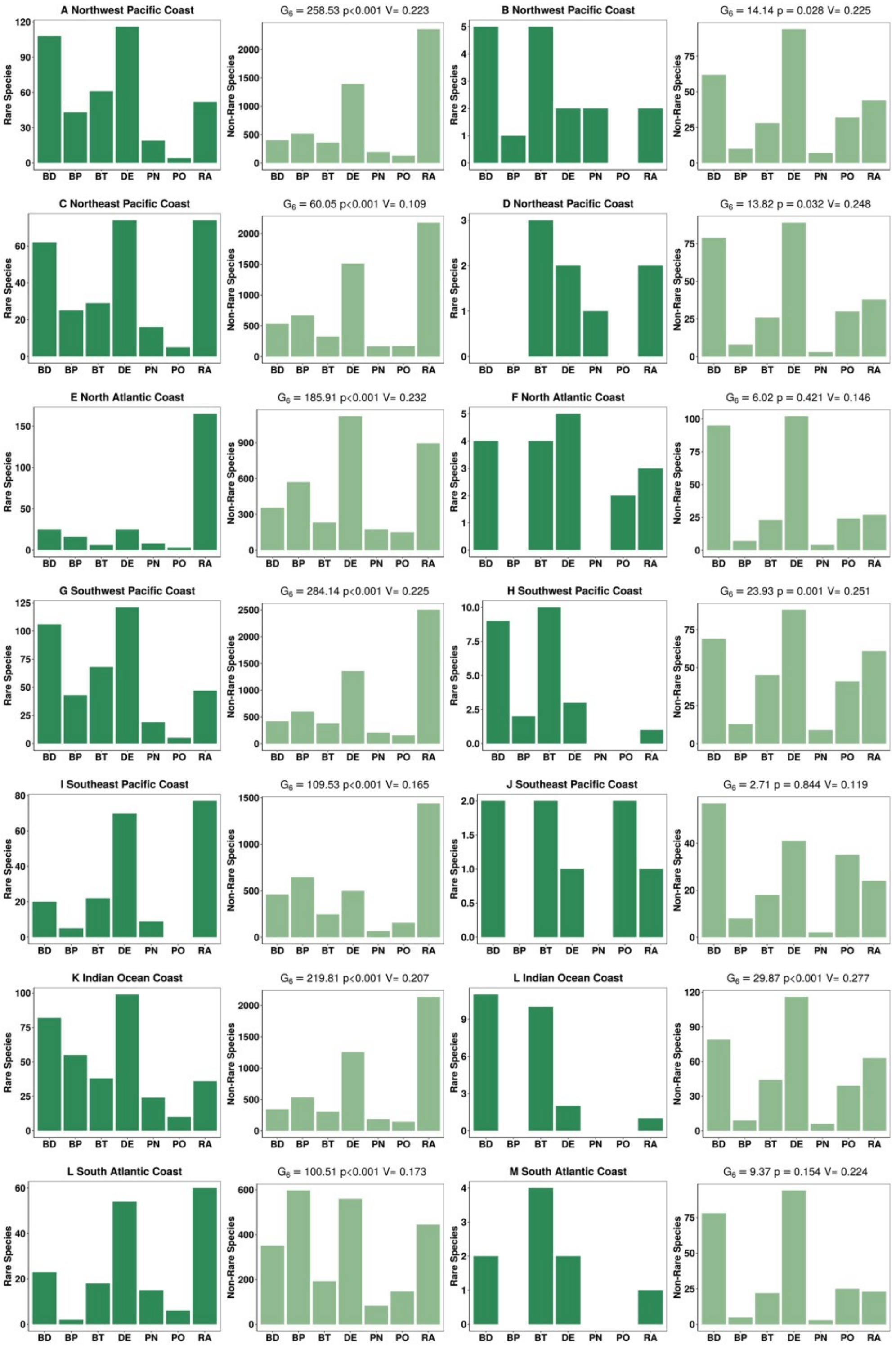

Supplementary Figure 10 | Habitat specialization distribution for rare species (dark green) and non-rare species (light green). The columns are as follows: BD = bathydemersal, BP = bathypelagic, BT = benthopelagic, DE = demersal, PN = pelagic_neritic, PO = pelagic_oceanic and RA = reef_associated. All bony fish are represented at the left side plots and the cartilaginous fish at right side plots. These analyses were done for all seven

coastal systems. The differences between the frequency distribution between rare and non-rare species are represented between the plots for rare and non-rare species as the values from the G- tests, p-values and Cramer`s V.

Supplementary Analysis

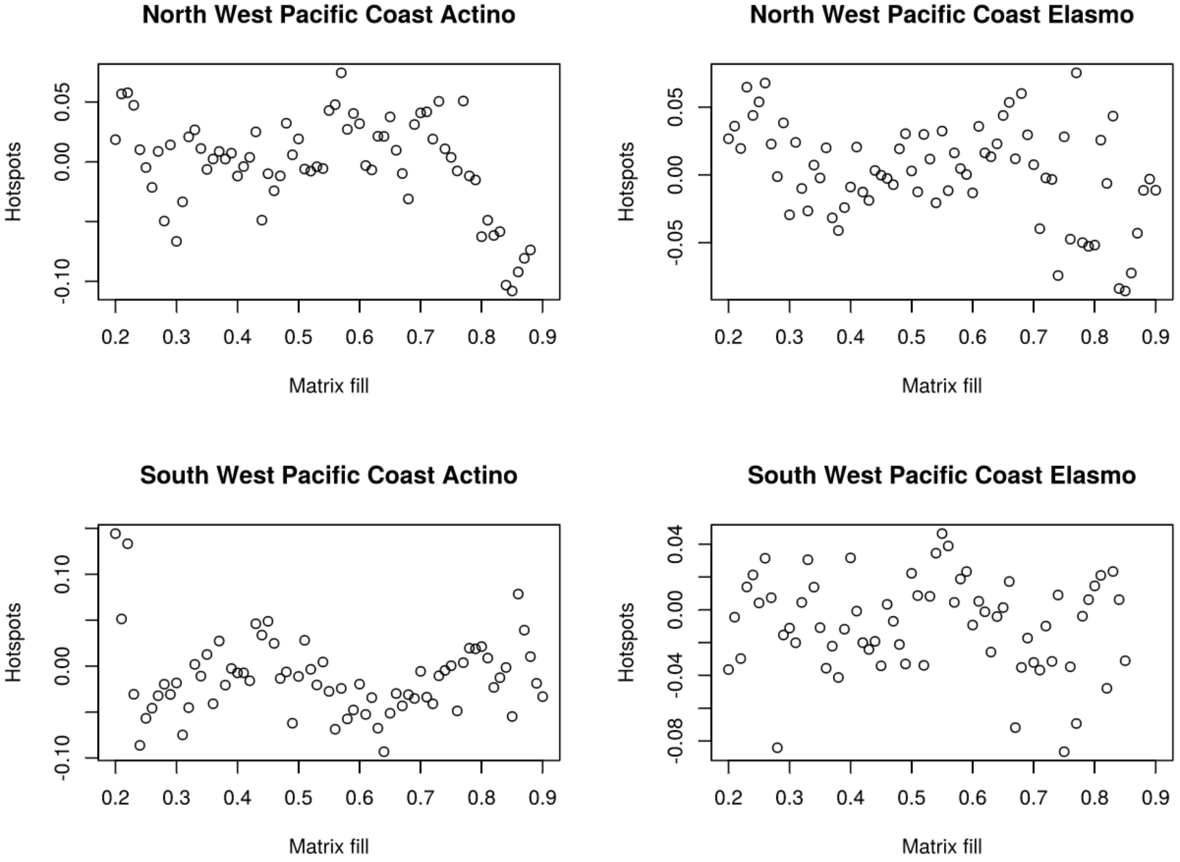

Supplementary Figure 11 | The relationship between the sampling effect and the observed rarity hotspots observed. This supplementary analysis asked if a random species draw (x-axis) in the locality matrix affects the estimation of rare species (y-axis). It was performed using as an example: North West Pacific Coast (bony and cartilaginous species) and South West Pacific Coast (bony and cartilaginous species). Matrix fill refers to fill of a random species while the y axis indicates the hotspots uncovered. As the plots illustrate there is no trend between the sampling effect and the identification of hotspots. These analyses were carried out in order to detect any potential bias related to sampling effect.

Supplementary Tables

Supplementary Table 1 Relevant classes of categorical trait variables used for our chosen species.

Body Shape	Reproductive Guild	Position in Water Column	Swimming Mode
angular	bearers	benthopelagic	amiiform
circular	bearers_and_guarders	pelagic_oceanic	anguilliform
compressed	bearers_external_brooders	bathydemersal	balistiform
eel_like	bearers_internal_live_bearers	demersal	carangiform
eel_like_angular	guarders	bathypelagic	diodontiform
eel_like_circular	guarders_brood_hiders	pelagic_neritic	gymnotiform
eel_like_compressed	guarders_clutch_tenders	reef_associated	labriform
eel_like_other	guarders_nesters	pelagic	ostraciiform
eel_like_oval	guarders_open_water_substratum_egg_scatterers		rajiform
elongated	mixed		subcarangiform
elongated_angular	nonguarders		tetraodontiform
elongated_circular	nonguarders_brood_hiders		thunniform
elongated_compressed	nonguarders_external_brooders		
elongated_flattened	nonguarders_nesters		
elongated_other	nonguarders_open_water_substratum_egg_scatterers		
elongated_oval			
flattened			
fusiform_normal			
fusiform_normal_angular			
fusiform_normal_circular			
fusiform_normal_compressed			
fusiform_normal_flattened			
fusiform_normal_other			
fusiform_normal_oval			
other			
other_angular			
other_compressed			
other_flattened			
other_oval			
others_flattened			
oval			
short_and_or_deep			
short_and_or_deep_angular			
short_and_or_deep_circular			

short_and_or_deep_compressed
short_and_or_deep_flattened
short_and_or_deep_other
short_and_or_deep_oval

Supplementary Table 2 (Please find the full list of rare species for each system at the end of this document)

Supplementary Methods

Code underpinning the analyses done in "**Global patterns in functional rarity of marine fish**".

All data necessary to run these analyses can be found at this repository – https://doi.org/10.17630/397bc872-f7de-4ded-9ed8-4f734c11b14a [1]

PART 1 - R CODE USED TO RUN THE MAIN ANALYSIS

```
### libraries ##########################################################

library(tidyverse)
library(funrar)

### functions ###########################################################

### simple function to swap between wide and long format

getMat<-function(f1) {
  widedf<-f1 %>% spread(key=GridID,value=num)
  widedf[is.na(widedf)]<-0
  return(widedf)
}

### scales given values within a group between 0 and 1

scale_value<-function(x) {
  scaled_x<-(x -min(x))/(max(x)-min(x))
  return(scaled_x)
}

### function takes the full data (get2DegSp) and subsets by both chosen probability
### (prob - between 0 and 1) and class (Elasmobranchii or Actinopterygii)

getProbClass<-function(prob, class) {

  getProb<-subset(get2DegSp, Probability > prob)

  ## merges the probability file with the 2 degree grid cells generated in ArcGIS

  probx<-merge(getProb, spCode, by="SpeciesID")
  probxy<-select(probx, gridID, species, Class.x, Probability, centLat2dG, centLong2d
G)
  colnames(probxy)<-c("GridID", "species", "Class", "Probability", "lat", "long")
```

```r

  getClassx<-subset(probxy, Class==class)
  getClassxy<-select(getClassx, GridID, species)
  classxy<-getClassxy %>% distinct()
  classxy$num<-1

  ## returns the long form file containing species and grid cell

  return(classxy)
}

### function to return the rarity values - uniqueness, restrictedness and
### global distinctiveness, both raw and scaled, for each species by region'
### region is one of the coast or open ocean regions, i.e. North_Atlantic_Coast,
### df is an empty dataframe

getRarityVals<-function(reg, trFile, spFile, df) {

  ## spFile is generated using the getProbClass() function, subset here
  ## based on region entered (reg)

  regFile<-subset(spFile, Region==reg)

  ## select only grid cells with at least 10 species

  numSp<-as.data.frame(regFile %>% group_by(GridID) %>% summarise(N=sum(num)))
  plus10<-subset(numSp, N >= 10)
  getRegFile<-merge(regFile, plus10, by="GridID")

  ## create a list of the unique species in the regional pool and use it to
  ## subset the trait file (trFile) - this is the full species x trait dataset
  ## for the chosen class

  listRegFile<-unique(getRegFile$species)
  tr2<-subset(trFile, Species %in% listRegFile)

  ## organise the categorical variables

  vr<-as.vector(unique(tr2$BodyShapIII_combined))
  rg<-as.vector(unique(tr2$RepGuildCombined))
  pwc<-as.vector(unique(tr2$PositionWaterColumn))
  sm<-as.vector(unique(tr2$SwimMode))
  tr3<-tr2

  ## convert to species x trait matrix

  tr4<-as.data.frame( matrix(NA, nrow(tr3), ncol(tr3),
      dimnames=list(row.names(tr3), names(tr3))))
  tr4[,"K"]<-as.numeric( tr3[,"K"] )
  tr4[,"Lm"]<-as.numeric( tr3[,"Lm"] )
  tr4[,"QB"]<-as.numeric( tr3[,"QB"] )
  tr4[,"Troph"]<-as.numeric( tr3[,"Troph"] )
  tr4[,"DepthMax"]<-as.numeric( tr3[,"DepthMax"] )
  tr4[,"TempPrefMean"]<-as.numeric( tr3[,"TempPrefMean"] )
  tr4[,"Generation_time"]<-as.numeric( tr3[,"Generation_time"] )
```

```
  tr4[,"BodyShapIII_combined"]<-factor(tr3[,"BodyShapIII_combined"], levels=vr, order
ed=FALSE)
  tr4[,"RepGuildCombined"]<-factor(tr3[,"RepGuildCombined"], levels=rg, ordered=FALSE
)
  tr4[,"PositionWaterColumn"]<-factor(tr3[,"PositionWaterColumn"], levels=pwc, ordere
d=FALSE)
  tr4[,"SwimMode"]<-factor(tr3[,"SwimMode"], levels=sm, ordered=FALSE)
  tr4<-tr4[-1]
  row.names(tr4)<-tr3[,1]

  ## calculate the distance matrix for the trait file

  traitT<-compute_dist_matrix(tr4, metric="gower", center=FALSE, scale=FALSE)

  ## select the relevant fields from the regional file and convert to wide format

  getRegFile2<-select(getRegFile, GridID, species, num)
  xsp<-getMat(getRegFile2)

  ## convert to a matrix and prepare for the funrar() function

  xsp<-setNames(data.frame(t(xsp[,-1])), xsp[,1])
  xsp1<-as.matrix(xsp)
  specT<-make_relative(xsp1)

  ## run the functions to calculate functional rarity and global  distinctiveness
  ## using the matrices traitT and specT

  specT<-specT[,rownames(traitT)]
  regFun<-funrar(specT, traitT, rel_abund=TRUE)
  globDist<-distinctiveness_global(traitT, di_name="global_di")

  ## extract the values for uniqueness, restrictedness and global distinctiveness
  ## and apply scale_value() function, then merge three together as a single file

  uniq<-regFun$Ui
  uniq$UiSc<-scale_value(uniq$Ui)
  rest<-regFun$Ri
  rest$RiSc<-scale_value(rest$Ri)
  uniqRest<-merge(uniq, rest, by="species")

  globDist$gdScale<-scale_value(globDist$global_di)
  URGD<-merge(uniqRest, globDist, by="species")

  ## add a field to identify region and coast/open ocean

  URGD$reg<-reg

  ## return data frame for the region

  return(URGD)
}

### function to calculate the top quantiles for restrictedness, distinctiveness
### and uniqueness and count the number of different species that are in the
```

```
### top quartile for both restrictedness and distinctiveness (GDR) and
### restrictedness and uniqueness (UR). This returns a data frame with the
### total species per grid cell plus number of UR and GDR species

getQuantSpecies<-function(regx, regName) {

  ## get the species totals per grid cell

  getNumSp<-as.data.frame(regx %>% group_by(GridID) %>%
      summarise(spNum=n_distinct(species)))

  ## calculate the quantiles for each rarity type - restrictedness (qr),
  ## uniqueness (qu) and global distinctiveness (qd)

  regx2<-unique(select(regx, -GridID))

  qu<-quantile(regx2$UiSc, c(0.25, 0.5, 0.75), type=1)
  qr<-quantile(regx2$RiSc, c(0.25, 0.5, 0.75), type=1)
  qd<-quantile(regx2$gdScale, c(0.25, 0.5, 0.75), type=1)

  ## ensure the inclusion of species with restrictedness=1

  if(qr[[3]]==1 ) {qr[[3]]<-0.99999999999}

  ## subset the region by species that are both unique and restricted (in
  ## the top quantile for each)

  uxr<-subset(regx, regx$UiSc>qu[[3]] & regx$RiSc>qr[[3]])

  ## subset the region by species that are both distinct and restricted
  ## (in the top quantile for each)

  gdxr<-subset(regx, regx$gdScale>qd[[3]] & regx$RiSc>qr[[3]])

  ## group by grid cell and total the rare (UR or GDR) species in each

  getURSp<-as.data.frame(uxr %>% group_by(GridID) %>%
      summarise(spUR=n_distinct(species)))
  getGDRSp<-as.data.frame(gdxr %>% group_by(GridID) %>%
      summarise(spGDR=n_distinct(species)))

  ## create joins between the total species and number of rare species
  ## per grid cell, convert NA to zeros and add a field for region name

  addUR<-left_join(getNumSp, getURSp, by="GridID")
  addGDR<-left_join(addUR, getGDRSp, by="GridID")
  addGDR[is.na(addGDR)]<-0
  addGDR$Region<-regName

  ## return the data frame

  return(addGDR)

}
```

```
### files ###############################################################

### read in the 2 degree grid cell file from Aquamaps - contains species and
### species codes, this is very large ~950MB

get2DegSp<-readRDS("gridCellsSpec2Deg.rds")

### read in the list of species, species codes and class

spCode<-read.csv("speciesClCodes.csv")[-1]

### read in the list of oceanic regions and the grid cells that fall within each

allOceans<-read.csv("allOceansRegions.csv")[-1]

### read the trait files for the relevant class -
### uncomment out to use Actinopterygii (or change names)

tr<-read.csv("elasmo_traits_global_rarity.csv") # Elasmobranchii
trF<-tr[-2]
trFile<-trF[-2]
#trFile<-read.csv("actino_traits_global_rarity.csv")[-1] # Actinopterygii


### generate results #####################################################

### for Elasmobranchii first

### run the getProbClass() function which will return the species file
### ready to be merged with the oceanic regions file

doSpFile<-getProbClass(0.9, "Elasmobranchii")
spFile<-merge(doSpFile, allOceans, by="GridID")

### create an empty data frame to hold results in the loop

df<-data.frame()

### loop through each region and calculate rarity based on the regional pool
### of species

for(reg in unique(allOceans$Region)) {

  getRar<-getRarityVals(reg, trFile, spFile, df)
  df<-rbind(df, getRar)
}

### can write out results file here

#saveRDS(df, "elasmoRegResults.rds")

###########################################################

### now Actinopterygii
```

```r

### read the trait file in here

trFile<-read.csv("actino_traits_global_rarity.csv")[-1] # Actinopterygii

### run the getProbClass() function which will return the species file
### ready to be merged with the oceanic regions file

doSpFile<-getProbClass(0.9, "Actinopterygii")
spFile<-merge(doSpFile, allOceans, by="GridID")

### create an empty data frame to hold results in the loop

df<-data.frame()

### loop through each region and calculate rarity based on the regional pool
### of species

for(reg in unique(allOceans$Region)) {

  getRar<-getRarityVals(reg, trFile, spFile, df)
  df<-rbind(df, getRar)
}

### can write out results file here

#saveRDS(df, "actinoRegResults.rds")


### quantiles ##############################################################


### read in the combined file of the results (as above) and the grid cells
### for each region

getSpID<-readRDS("elasmoGridsRes.rds")
getSp2<-select(getSpID, species, Region, GridID)
useDf<-readRDS("elasmoRegResults.rds")
useDf$Region<-useDf$reg
doDf<-select(useDf, -reg)
elGID<-merge(getSp2, doDf, by=c("species", "Region"))
saveRDS(elGID, "elasmoGridResults.rds")

### first for Elasmobranchii

getSp<-readRDS("elasmoGridResults.rds")

### create an empty data frame

dfG<-data.frame()

### now the loop

for(regName in unique(getSp$Region)) {

```

```
  ## subset for the region (loops through each one)

  regx<-subset(getSp, Region==regName)

  ## call the function to generate the numbers of rare species and species
  ## totals per grid cell

  quantRar<-getQuantSpecies(regx, regName)

  ## add to data frame

  dfG<-rbind(dfG, quantRar)

}

### can save file as an RDS

#saveRDS(dfG, "elasmoQuantRarityNums.rds")


### now for Actinopterygii

getSpID<-readRDS("actinoGridsRes.rds")
getSp2<-select(getSpID, species, Region, GridID)
useDf<-readRDS("actinoRegResults.rds")
useDf$Region<-useDf$reg
doDf<-select(useDf, -reg)
elGID<-merge(getSp2, doDf, by=c("species", "Region"))
saveRDS(elGID, "actinoGridResults.rds")

getSp<-readRDS("actinoGridResults.rds")

### create an empty data frame

dfG<-data.frame()

### now the loop

for(regName in unique(getSp$Region)) {

  ## subset for the region (loops through each one)

  regx<-subset(getSp, Region==regName)

  ## call the function to generate the numbers of rare species and species
  ## totals per grid cell

  quantRar<-getQuantSpecies(regx, regName)

  ## add to data frame

  dfG<-rbind(dfG, quantRar)

}
```

```
### can save file as an RDS

#saveRDS(dfG, "actinoQuantRarityNums.rds")
```

PART 2 - PYTHON CODE USED TO RUN THE NULL MODEL

After running the code above, use as Input for the Null Model both of the following files: 1 - The assemblage matrix for each system, save it at the "Matrix" folder (as shown at the schematic representation below), 2 – The list of rare species found for each system, save at the "Rare Species" folder. The Python code will use those standard file names to proceed with the analysis.

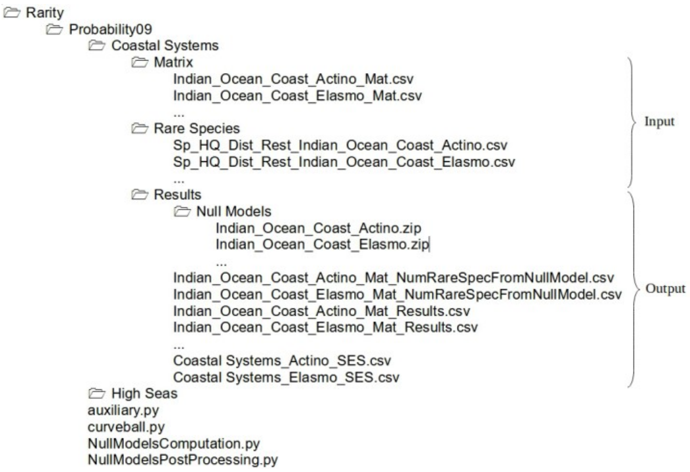

Schematic Representation 1 Folder`s structure for the Input files and Output files.

NullModelsComputation.py

```
import os
import csv
import numpy as np
import shutil
from curveball import * # Code from Strona et al. 2014 [2]
from auxiliary import *

""" This file computes the null models and the resulting SES """

## INPUTS ---------------------------------------------------------------

Reference = "Coastal Systems "      # Name of main folder
AbsencePresenceFolder = "Matrix"    # Name of subfolder containing absence/presence m
atrix
RareSpeciesFolder = "Rare species"  # Name of subfolder containing rare species list
outfolder = "Results"               # Name of output subfolder (it will be automatica
lly created if missing)

NumIter = 2000                      # Number of interactions
b_SaveNullModels = True             # Save all the null models or not
```

```
NMfolder = "Null Models"            # Name of null models folder (subfolder of outfolder)

b_AddModels = False                 # Specify whether to add models to previously calculated ones
b_setFixedSES = True                # Where no rare species occurred in the null models, set SES to:
                                    #   - zero in grid cells with no real rare species (to prevent NaN);
                                    #   - number of real rare species in grid cells with real rare species (to prevent inf);

## READ FILE ------------------------------------------------------------

AbsencePresenceFolder = os.path.join(Reference,AbsencePresenceFolder)
RareSpeciesFolder = os.path.join(Reference,RareSpeciesFolder)

# If output folder doesn't exist, create it
outpath = os.path.join(os.getcwd(),Reference,outfolder)
if not os.path.isdir(outpath):
    os.mkdir(outpath)
if b_SaveNullModels:
    NMpath = os.path.join(outpath,NMfolder)
    if not os.path.isdir(NMpath):
        os.mkdir(NMpath)

# Get all files in AbsencePresenceFolder
FileList = [file for file in os.listdir(AbsencePresenceFolder) if os.path.isfile(os.path.join(AbsencePresenceFolder, file))]

for filename in FileList:

    # Retrieve file names
    basename = filename.split('_Mat.csv')[0]
    AbsencePresenceFile = basename + '_Mat.csv'
    RareSpeciesFile = "Sp_HQ_Dist_Rest_" + basename + ".csv"

    print("Running %s..." % basename)

    # Extract previous results under request
    NMpath = os.path.join(outpath,NMfolder,basename)
    extractpath = os.path.join(outpath,NMfolder,basename)
    if b_AddModels:
        shutil.unpack_archive(NMpath + '.zip', extractpath)
```

```
        kstart = len([file for file in os.listdir(extractpath) if os.path.isfile(os.path.join(extractpath, file))])+1
    else:
        if os.path.isdir(extractpath):
            shutil.rmtree(extractpath)
        kstart = 0

    # Import rare species
    RareSpecs = []
    with open(os.path.join(RareSpeciesFolder, RareSpeciesFile)) as csv_file:
        csv_reader = csv.reader(csv_file, delimiter=',')
        for row in csv_reader:
            RareSpecs.append(row)

    del(RareSpecs[0])
    for i in range(len(RareSpecs)):
        del(RareSpecs[i][:1])
    RareSpecs = [x[0] for x in RareSpecs]

    # Import absence/presence file
    FileContent = []
    with open(os.path.join(AbsencePresenceFolder, AbsencePresenceFile)) as csv_file:
        csv_reader = csv.reader(csv_file, delimiter=',')
        for row in csv_reader:
            FileContent.append(row)

    # Find Columns with Rare Species
    TotSpecies = FileContent[0][2:]
    RareSpecCol = [TotSpecies.index(x) for x in RareSpecs]

    # Get GridCells
    GridCells = [FileContent[x][1] for x in range(1,len(FileContent))]

    # Get the absence/presence matrix
    del(FileContent[0])
    for i in range(len(FileContent)):
        del(FileContent[i][:2])

    Nrow = len(FileContent)
    Ncol = len(FileContent[0])

    M = np.zeros((Nrow,Ncol))
    for i in range(Nrow):
        for j in range(Ncol):
            M[i,j] = float(FileContent[i][j])
```

```python

    ## CALCULATE ----------------------------------------------------------

    # Generate subfolder for saving null models for the current filename
    if b_SaveNullModels:
        NMpath = os.path.join(outpath,NMfolder,basename)
        if not os.path.isdir(NMpath):
            os.mkdir(NMpath)

    # Generate the Null Models
    r_hp = find_presences(M)
    NumRareSpec = np.zeros((len(GridCells),NumIter))
    for k in range(NumIter):
        # Get Null Model
        RM = curve_ball(M,r_hp)
        if b_SaveNullModels:
            fname = os.path.join(NMpath,os.path.splitext(AbsencePresenceFile)[0] + "_NM_%04.0f" % (kstart+k) + ".csv")
            NM = concatenate_list([GridCells,RM.tolist()])
            header = ["GridID"] + TotSpecies
            csvwrite(fname,NM,header)
        # Get Number of rare species for each grid cell
        RM_RareSpecs = RM[:,RareSpecCol]
        NumRareSpec[:,k] = np.sum(RM_RareSpecs,axis=1)

    # Load previous results and merge with current ones
    if b_AddModels:
        fname = os.path.join(outpath,os.path.splitext(AbsencePresenceFile)[0] + "_NumRareSpecFromNullModel.csv")
        NumRareSpecFromFile = []
        with open(fname) as csv_file:
            csv_reader = csv.reader(csv_file, delimiter=',')
            for row in csv_reader:
                NumRareSpecFromFile.append(row)
        del(NumRareSpecFromFile[0])
        for i in range(len(NumRareSpecFromFile)):
            del(NumRareSpecFromFile[i][:3])
        NumRareSpecFromFileArray = np.zeros((Nrow,len(NumRareSpecFromFile[0])))
        for i in range(Nrow):
            for j in range(len(NumRareSpecFromFile[0])):
                NumRareSpecFromFileArray[i,j] = float(NumRareSpecFromFile[i][j])
        NumRareSpec = np.concatenate((NumRareSpecFromFileArray,NumRareSpec),axis=1)

    # Calculate SES
    NumRareSpecReal = np.sum(M[:,RareSpecCol],axis=1)
```

```
    AvgNumRareSpecs = np.mean(NumRareSpec,axis=1)
    StdNumRareSpecs = np.std(NumRareSpec,axis=1)
    SES = (NumRareSpecReal - AvgNumRareSpecs)/StdNumRareSpecs

    # Set SES to zero in grid cells where no rare specied occurred (neither real nor in null models)
    # Set SES to no. of real rare species in grid cells where no rare species occurred in null models
    if b_setFixedSES:
        SES[np.isnan(SES)] = 0
        SES[np.isinf(SES)] = NumRareSpecReal[np.isinf(SES)]

    # Save Results
    fname = os.path.join(outpath,os.path.splitext(AbsencePresenceFile)[0] + "_NumRareSpecFromNullModel.csv")
    filecontent = concatenate_list([NumRareSpecReal.tolist(),SES.tolist(),GridCells,NumRareSpec.tolist()])
    header = ["Real","SES","GridID"] + ["NM_%04.0f" % x for x in range(len(NumRareSpec[0]))]
    csvwrite(fname,filecontent,header)

    fname = os.path.join(outpath,os.path.splitext(AbsencePresenceFile)[0] + "_Results.csv")
    filecontent = concatenate_list([GridCells,NumRareSpecReal.tolist(),SES.tolist(),[basename for x in range(Nrow)]])
    header = ["GridID","Real","SES",Reference]
    csvwrite(fname,filecontent,header)

    if b_SaveNullModels:
        # zip results
        shutil.make_archive(NMpath, 'zip', NMpath)
        # delete folder
        shutil.rmtree(NMpath)

print("Done.")

```

NullModelsPostProcessing.py

```
import os
import csv
import numpy as np
import shutil
from auxiliary import *

```

```
""" This file gathers the resulting SES for each system in a single file,
    one for Actino and one for Elasmos"""

## INPUTS ------------------------------------------------------------------

Reference = "Coastal Systems "  # Name of main folder
outfolder = "Results"           # Name of output subfolder

## READ FILE ---------------------------------------------------------------

outfolder = os.path.join(Reference,outfolder)
FileList = [file for file in os.listdir(outfolder) if file.endswith("_NumRareSpecFrom
NullModel.csv")]
SubsetNames = ["Actino","Elasmo"]
Subsets = [[file for file in FileList if Subset in file] for Subset in SubsetNames] #
 [ActinoFiles,ElasmoFiles]

# Loop Actino and Elasmos separately
for kk in range(len(Subsets)):

    basename = []
    GridCells = []
    NumRareSpecReal = []
    SES = []

    # Read the data from all files
    for filename in Subsets[kk]:

        # Retrieve file names
        basename = filename.split("_Mat_NumRareSpecFromNullModel.csv")[0]

        print("Loading %s..." % basename)

        # Import Results file
        FileContent = []
        with open(os.path.join(outfolder, filename)) as csv_file:
            csv_reader = csv.reader(csv_file, delimiter=',')
            for row in csv_reader:
                FileContent.append(row)

        # Get Real Number Of Rare Species
        NumRareSpecReal.append([FileContent[x][0] for x in range(1,len(FileContent))]
)

        # Get SES
```

```
        SES.append([float(FileContent[x][1]) for x in range(1,len(FileContent))])

        # Get GridCells
        GridCells.append([FileContent[x][2] for x in range(1,len(FileContent))])

    # Get max and min overall SES
    minSES = min([min(SES[x])for x in range(len(SES))])
    maxSES = max([max(SES[x])for x in range(len(SES))])

    # Start writing file
    fname = os.path.join(outfolder,Reference + "_" + SubsetNames[kk] + "_SES.csv")
    header = ["GridID","Real ","SES ",Reference]
    f = open(fname, 'w')
    writer = csv.writer(f, lineterminator = '\n')
    writer.writerow(header)

    # Calculate the scaled SES
    for k in range(len(Subsets[kk])):

        filename = Subsets[kk][k]
        basename = filename.split("_Mat_NumRareSpecFromNullModel.csv")[0]
        Nrow = len(GridCells[k])

        # Save Results
        filecontent = concatenate_list([GridCells[k],NumRareSpecReal[k],SES[k],[basename for x in range(Nrow)]])
        writer.writerows(filecontent)
    f.close()

print("Done.")
```

auxiliary.py

```
import csv

""" Auxiliary functions for null model computations """

def csvwrite(filename, data, header):
    writer = csv.writer(open(filename, 'w'), lineterminator = '\n')
    writer.writerow(header)
    writer.writerows(data)


def concatenate_list(data):
    # Check format of input
```

```
    for i in range(len(data)):
        if not isinstance(data[i][0], list):
            data[i] = [[x] for x in data[i]]
    # Concatenate by column
    M = data[0]
    for j in range(1,len(data)):
        for i in range(len(M)):
          M[i].extend(data[j][i])
    return(M)
```

Supplementary References

1 Trindade-Santos, I., Moyes, F. & Magurran, A. Data Underpinning Global patterns in functional rarity of marine fish. University of St Andrews, doi: https://doi.org/10.17630/397bc872-f7de-4ded-9ed8-4f734c11b14a (2022).

2 Strona, G., Nappo, D., Boccacci, F., Fattorini, S. & San-Miguel-Ayanz, J. A fast and unbiased procedure to randomize ecological binary matrices with fixed row and column totals. *Nature communications* **5**, 1-9 (2014).

Supplementary Table 2 List of rare species found for each System and Class, the indices used here were restrictedness and distinctiveness, and probability higher than 0.9.

Rare Species	System	Class
Acanthanectes hystrix	Indian Ocean Coast	Actinopterygii
Adventor elongatus	Indian Ocean Coast	Actinopterygii
Alabes elongata	Indian Ocean Coast	Actinopterygii
Alabes gibbosa	Indian Ocean Coast	Actinopterygii
Alepocephalus blanfordii	Indian Ocean Coast	Actinopterygii
Alepocephalus productus	Indian Ocean Coast	Actinopterygii
Allenichthys glauerti	Indian Ocean Coast	Actinopterygii
Allocyttus guineensis	Indian Ocean Coast	Actinopterygii
Allocyttus niger	Indian Ocean Coast	Actinopterygii
Amblyeleotris latifasciata	Indian Ocean Coast	Actinopterygii
Amblygobius stethophthalmus	Indian Ocean Coast	Actinopterygii
Andamia heteroptera	Indian Ocean Coast	Actinopterygii
Anguilla australis	Indian Ocean Coast	Actinopterygii
Anguilla celebesensis	Indian Ocean Coast	Actinopterygii
Anguilla nebulosa	Indian Ocean Coast	Actinopterygii
Anguilla reinhardtii	Indian Ocean Coast	Actinopterygii
Antigonia rhomboidea	Indian Ocean Coast	Actinopterygii
Aphanopus microphthalmus	Indian Ocean Coast	Actinopterygii
Apogonichthyoides brevicaudatus	Indian Ocean Coast	Actinopterygii
Apopterygion alta	Indian Ocean Coast	Actinopterygii
Argyrosomus hololepidotus	Indian Ocean Coast	Actinopterygii
Argyrosomus inodorus	Indian Ocean Coast	Actinopterygii
Argyrosomus regius	Indian Ocean Coast	Actinopterygii
Ariomma parini	Indian Ocean Coast	Actinopterygii
Ariosoma sokotranum	Indian Ocean Coast	Actinopterygii
Arius gagora	Indian Ocean Coast	Actinopterygii
Arothron carduus	Indian Ocean Coast	Actinopterygii
Arotrolepis filicauda	Indian Ocean Coast	Actinopterygii
Aspasmogaster occidentalis	Indian Ocean Coast	Actinopterygii
Assiculoides desmonotus	Indian Ocean Coast	Actinopterygii
Asterropteryx atripes	Indian Ocean Coast	Actinopterygii
Bathophilus vaillanti	Indian Ocean Coast	Actinopterygii
Bathyaploactis curtisensis	Indian Ocean Coast	Actinopterygii
Bathycongrus macrocercus	Indian Ocean Coast	Actinopterygii
Bathycongrus nasicus	Indian Ocean Coast	Actinopterygii
Bathydraco macrolepis	Indian Ocean Coast	Actinopterygii
Bathygobius karachiensis	Indian Ocean Coast	Actinopterygii
Bathyonus laticeps	Indian Ocean Coast	Actinopterygii
Bathyphylax omen	Indian Ocean Coast	Actinopterygii
Bathypterois filiferus	Indian Ocean Coast	Actinopterygii
Bathypterois grallator	Indian Ocean Coast	Actinopterygii
Bathypterois longipes	Indian Ocean Coast	Actinopterygii
Bathypterois phenax	Indian Ocean Coast	Actinopterygii

Rare Species	System	Class
Bathytroctes microlepis	Indian Ocean Coast	Actinopterygii
Bathytyphlops sewelli	Indian Ocean Coast	Actinopterygii
Benthodesmus macrophthalmus	Indian Ocean Coast	Actinopterygii
Bothus podas	Indian Ocean Coast	Actinopterygii
Brachionichthys australis	Indian Ocean Coast	Actinopterygii
Bryaninops nexus	Indian Ocean Coast	Actinopterygii
Bufoceratias wedli	Indian Ocean Coast	Actinopterygii
Callionymus tenuis	Indian Ocean Coast	Actinopterygii
Campichthys tricarinatus	Indian Ocean Coast	Actinopterygii
Careproctus albescens	Indian Ocean Coast	Actinopterygii
Careproctus improvisus	Indian Ocean Coast	Actinopterygii
Careproctus vladibeckeri	Indian Ocean Coast	Actinopterygii
Cataetyx bruuni	Indian Ocean Coast	Actinopterygii
Cataetyx laticeps	Indian Ocean Coast	Actinopterygii
Cataetyx niki	Indian Ocean Coast	Actinopterygii
Chaenophryne ramifera	Indian Ocean Coast	Actinopterygii
Champsocephalus gunnari	Indian Ocean Coast	Actinopterygii
Chaunacops coloratus	Indian Ocean Coast	Actinopterygii
Chaunacops melanostomus	Indian Ocean Coast	Actinopterygii
Chaunax nebulosus	Indian Ocean Coast	Actinopterygii
Chelon tricuspidens	Indian Ocean Coast	Actinopterygii
Chlidichthys rubiceps	Indian Ocean Coast	Actinopterygii
Chlorurus microrhinos	Indian Ocean Coast	Actinopterygii
Chlorurus troschelii	Indian Ocean Coast	Actinopterygii
Choeroichthys suillus	Indian Ocean Coast	Actinopterygii
Chrysoblephus cristiceps	Indian Ocean Coast	Actinopterygii
Cirrhilabrus joanallenae	Indian Ocean Coast	Actinopterygii
Cirrhimuraena tapeinoptera	Indian Ocean Coast	Actinopterygii
Cirripectes randalli	Indian Ocean Coast	Actinopterygii
Coelorinchus fasciatus	Indian Ocean Coast	Actinopterygii
Coelorinchus innotabilis	Indian Ocean Coast	Actinopterygii
Coelorinchus kaiyomaru	Indian Ocean Coast	Actinopterygii
Coloconger eximia	Indian Ocean Coast	Actinopterygii
Coryphaenoides ferrieri	Indian Ocean Coast	Actinopterygii
Coryphaenoides hoskynii	Indian Ocean Coast	Actinopterygii
Coryphaenoides lecointei	Indian Ocean Coast	Actinopterygii
Coryphaenoides marshalli	Indian Ocean Coast	Actinopterygii
Coryphaenoides rudis	Indian Ocean Coast	Actinopterygii
Coryphaenoides serrulatus	Indian Ocean Coast	Actinopterygii
Cosmocampus retropinnis	Indian Ocean Coast	Actinopterygii
Cottunculus spinosus	Indian Ocean Coast	Actinopterygii
Crenimugil heterocheilos	Indian Ocean Coast	Actinopterygii
Danaphryne nigrifilis	Indian Ocean Coast	Actinopterygii
Dentex gibbosus	Indian Ocean Coast	Actinopterygii
Dermatopsoides morrisonae	Indian Ocean Coast	Actinopterygii

Rare Species	System	Class
Dibranchus atlanticus	Indian Ocean Coast	Actinopterygii
Dibranchus nasutus	Indian Ocean Coast	Actinopterygii
Dibranchus tremendus	Indian Ocean Coast	Actinopterygii
Dicentrarchus punctatus	Indian Ocean Coast	Actinopterygii
Dicrolene introniger	Indian Ocean Coast	Actinopterygii
Dieidolycus leptodermatus	Indian Ocean Coast	Actinopterygii
Diplecogaster tonstricula	Indian Ocean Coast	Actinopterygii
Diretmoides pauciradiatus	Indian Ocean Coast	Actinopterygii
Dodekablennos fraseri	Indian Ocean Coast	Actinopterygii
Dolichosudis fuliginosa	Indian Ocean Coast	Actinopterygii
Dolopichthys pullatus	Indian Ocean Coast	Actinopterygii
Dysalotus oligoscolus	Indian Ocean Coast	Actinopterygii
Dysomma bucephalus	Indian Ocean Coast	Actinopterygii
Echelus pachyrhynchus	Indian Ocean Coast	Actinopterygii
Ecsenius aroni	Indian Ocean Coast	Actinopterygii
Ecsenius dentex	Indian Ocean Coast	Actinopterygii
Ecsenius lubbocki	Indian Ocean Coast	Actinopterygii
Ecsenius paroculus	Indian Ocean Coast	Actinopterygii
Eleotris lutea	Indian Ocean Coast	Actinopterygii
Ematops randalli	Indian Ocean Coast	Actinopterygii
Enchelybrotula paucidens	Indian Ocean Coast	Actinopterygii
Engraulis capensis	Indian Ocean Coast	Actinopterygii
Engraulis encrasicolus	Indian Ocean Coast	Actinopterygii
Enigmacanthus filamentosus	Indian Ocean Coast	Actinopterygii
Enneapterygius kosiensis	Indian Ocean Coast	Actinopterygii
Enneapterygius qirmiz	Indian Ocean Coast	Actinopterygii
Enneapterygius ziegleri	Indian Ocean Coast	Actinopterygii
Entomacrodus lemuria	Indian Ocean Coast	Actinopterygii
Epinephelus caninus	Indian Ocean Coast	Actinopterygii
Eubalichthys bucephalus	Indian Ocean Coast	Actinopterygii
Eubalichthys caeruleoguttatus	Indian Ocean Coast	Actinopterygii
Eugnathogobius illotus	Indian Ocean Coast	Actinopterygii
Eugnathogobius variegatus	Indian Ocean Coast	Actinopterygii
Eurypleuron owasianum	Indian Ocean Coast	Actinopterygii
Eustomias lipochirus	Indian Ocean Coast	Actinopterygii
Eusurculus andamanensis	Indian Ocean Coast	Actinopterygii
Eviota afelei	Indian Ocean Coast	Actinopterygii
Eviota atriventris	Indian Ocean Coast	Actinopterygii
Eviota lachdeberei	Indian Ocean Coast	Actinopterygii
Eviota latifasciata	Indian Ocean Coast	Actinopterygii
Eviota rubriceps	Indian Ocean Coast	Actinopterygii
Eviota rubrisparsa	Indian Ocean Coast	Actinopterygii
Eviota sodwanaensis	Indian Ocean Coast	Actinopterygii
Genypterus blacodes	Indian Ocean Coast	Actinopterygii
Genypterus capensis	Indian Ocean Coast	Actinopterygii

Rare Species	System	Class
Genypterus tigerinus	Indian Ocean Coast	Actinopterygii
Gigantactis elsmani	Indian Ocean Coast	Actinopterygii
Gigantactis kreffti	Indian Ocean Coast	Actinopterygii
Gigantactis vanhoeffeni	Indian Ocean Coast	Actinopterygii
Gobiodon albofasciatus	Indian Ocean Coast	Actinopterygii
Gobiodon ater	Indian Ocean Coast	Actinopterygii
Gobionotothen acuta	Indian Ocean Coast	Actinopterygii
Gobionotothen gibberifrons	Indian Ocean Coast	Actinopterygii
Gobionotothen marionensis	Indian Ocean Coast	Actinopterygii
Gyrinomimus grahami	Indian Ocean Coast	Actinopterygii
Halichoeres claudia	Indian Ocean Coast	Actinopterygii
Haliichthys taeniophorus	Indian Ocean Coast	Actinopterygii
Halosaurus attenuatus	Indian Ocean Coast	Actinopterygii
Halosaurus guentheri	Indian Ocean Coast	Actinopterygii
Harpadon squamosus	Indian Ocean Coast	Actinopterygii
Harpagifer kerguelensis	Indian Ocean Coast	Actinopterygii
Helcogramma capidata	Indian Ocean Coast	Actinopterygii
Helcogramma maldivensis	Indian Ocean Coast	Actinopterygii
Helicolenus mouchezi	Indian Ocean Coast	Actinopterygii
Hetereleotris bipunctata	Indian Ocean Coast	Actinopterygii
Hetereleotris georgegilli	Indian Ocean Coast	Actinopterygii
Heteroclinus johnstoni	Indian Ocean Coast	Actinopterygii
Himantolophus albinares	Indian Ocean Coast	Actinopterygii
Himantolophus paucifilosus	Indian Ocean Coast	Actinopterygii
Himantolophus rostratus	Indian Ocean Coast	Actinopterygii
Hippocampus bargibanti	Indian Ocean Coast	Actinopterygii
Hippocampus biocellatus	Indian Ocean Coast	Actinopterygii
Hippocampus camelopardalis	Indian Ocean Coast	Actinopterygii
Hippocampus comes	Indian Ocean Coast	Actinopterygii
Hippocampus jayakari	Indian Ocean Coast	Actinopterygii
Hippocampus minotaur	Indian Ocean Coast	Actinopterygii
Hippocampus montebelloensis	Indian Ocean Coast	Actinopterygii
Hippocampus multispinus	Indian Ocean Coast	Actinopterygii
Hippocampus satomiae	Indian Ocean Coast	Actinopterygii
Hippocampus subelongatus	Indian Ocean Coast	Actinopterygii
Histiobranchus bathybius	Indian Ocean Coast	Actinopterygii
Holcomycteronus profundissimus	Indian Ocean Coast	Actinopterygii
Hyporhamphus erythrorinchus	Indian Ocean Coast	Actinopterygii
Hyporhamphus picarti	Indian Ocean Coast	Actinopterygii
Hyporthodus haifensis	Indian Ocean Coast	Actinopterygii
Hypselognathus horridus	Indian Ocean Coast	Actinopterygii
Ijimaia loppei	Indian Ocean Coast	Actinopterygii
Ipnops murrayi	Indian Ocean Coast	Actinopterygii
Istiblennius rivulatus	Indian Ocean Coast	Actinopterygii
Istiblennius unicolor	Indian Ocean Coast	Actinopterygii

**Rare Species**	**System**	**Class**
Jaydia melanopus	Indian Ocean Coast	Actinopterygii
Kajikia albida	Indian Ocean Coast	Actinopterygii
Kuiterichthys furcipilis	Indian Ocean Coast	Actinopterygii
Kyphosus gladius	Indian Ocean Coast	Actinopterygii
Labichthys carinatus	Indian Ocean Coast	Actinopterygii
Lampadena dea	Indian Ocean Coast	Actinopterygii
Lepidion microcephalus	Indian Ocean Coast	Actinopterygii
Leptocephalus giganteus	Indian Ocean Coast	Actinopterygii
Leptocephalus ophichthoides	Indian Ocean Coast	Actinopterygii
Leptostomias longibarba	Indian Ocean Coast	Actinopterygii
Lepturacanthus pantului	Indian Ocean Coast	Actinopterygii
Lestidiops similis	Indian Ocean Coast	Actinopterygii
Lindbergichthys mizops	Indian Ocean Coast	Actinopterygii
Linophryne arcturi	Indian Ocean Coast	Actinopterygii
Linophryne densiramus	Indian Ocean Coast	Actinopterygii
Linophryne pennibarbata	Indian Ocean Coast	Actinopterygii
Linophryne racemifera	Indian Ocean Coast	Actinopterygii
Lobulogobius morrigu	Indian Ocean Coast	Actinopterygii
Lophius vomerinus	Indian Ocean Coast	Actinopterygii
Lophotus capellei	Indian Ocean Coast	Actinopterygii
Luciobrotula corethromycter	Indian Ocean Coast	Actinopterygii
Luciobrotula nolfi	Indian Ocean Coast	Actinopterygii
Lycenchelys hureaui	Indian Ocean Coast	Actinopterygii
Lycodonus vermiformis	Indian Ocean Coast	Actinopterygii
Macrourus carinatus	Indian Ocean Coast	Actinopterygii
Macruronus capensis	Indian Ocean Coast	Actinopterygii
Macruronus novaezelandiae	Indian Ocean Coast	Actinopterygii
Magnisudis atlantica	Indian Ocean Coast	Actinopterygii
Maurolicus mucronatus	Indian Ocean Coast	Actinopterygii
Melamphaes suborbitalis	Indian Ocean Coast	Actinopterygii
Melanocetus murrayi	Indian Ocean Coast	Actinopterygii
Merluccius capensis	Indian Ocean Coast	Actinopterygii
Merluccius paradoxus	Indian Ocean Coast	Actinopterygii
Micrognathus micronotopterus	Indian Ocean Coast	Actinopterygii
Microlophichthys microlophus	Indian Ocean Coast	Actinopterygii
Microphis lineatus	Indian Ocean Coast	Actinopterygii
Mitotichthys tuckeri	Indian Ocean Coast	Actinopterygii
Monomitopus longiceps	Indian Ocean Coast	Actinopterygii
Monomitopus vitiazi	Indian Ocean Coast	Actinopterygii
Mugilogobius littoralis	Indian Ocean Coast	Actinopterygii
Muraenolepis microps	Indian Ocean Coast	Actinopterygii
Mycteroperca rubra	Indian Ocean Coast	Actinopterygii
Myersina crocata	Indian Ocean Coast	Actinopterygii
Nannocampus subosseus	Indian Ocean Coast	Actinopterygii
Nemadactylus vemae	Indian Ocean Coast	Actinopterygii

**Rare Species**	**System**	**Class**
Neobythites multistriatus	Indian Ocean Coast	Actinopterygii
Neocaristius heemstrai	Indian Ocean Coast	Actinopterygii
Neocyema erythrosoma	Indian Ocean Coast	Actinopterygii
Neomerinthe bauchotae	Indian Ocean Coast	Actinopterygii
Nerophis maculatus	Indian Ocean Coast	Actinopterygii
Nesogobius maccullochi	Indian Ocean Coast	Actinopterygii
Nezumia brevibarbata	Indian Ocean Coast	Actinopterygii
Nezumia sclerorhynchus	Indian Ocean Coast	Actinopterygii
Nezumia umbracincta	Indian Ocean Coast	Actinopterygii
Notomuraenobathys microcephalus	Indian Ocean Coast	Actinopterygii
Nototheniops larseni	Indian Ocean Coast	Actinopterygii
Obliquogobius turkayi	Indian Ocean Coast	Actinopterygii
Odontamblyopus rubicundus	Indian Ocean Coast	Actinopterygii
Oneirodes anisacanthus	Indian Ocean Coast	Actinopterygii
Oneirodes carlsbergi	Indian Ocean Coast	Actinopterygii
Oneirodes eschrichtii	Indian Ocean Coast	Actinopterygii
Oneirodes macrosteus	Indian Ocean Coast	Actinopterygii
Oneirodes myrionemus	Indian Ocean Coast	Actinopterygii
Oneirodes theodoritissieri	Indian Ocean Coast	Actinopterygii
Opistognathus cyanospilotus	Indian Ocean Coast	Actinopterygii
Orcynopsis unicolor	Indian Ocean Coast	Actinopterygii
Ostorhinchus melanoproctus	Indian Ocean Coast	Actinopterygii
Pachycara crassiceps	Indian Ocean Coast	Actinopterygii
Pagrus pagrus	Indian Ocean Coast	Actinopterygii
Parablennius cyclops	Indian Ocean Coast	Actinopterygii
Parabrotula plagiophthalma	Indian Ocean Coast	Actinopterygii
Paracaristius maderensis	Indian Ocean Coast	Actinopterygii
Paraliparis antarcticus	Indian Ocean Coast	Actinopterygii
Paraliparis charcoti	Indian Ocean Coast	Actinopterygii
Paraliparis costatus	Indian Ocean Coast	Actinopterygii
Paraliparis gomoni	Indian Ocean Coast	Actinopterygii
Paraliparis obliquosus	Indian Ocean Coast	Actinopterygii
Paraliparis operculosus	Indian Ocean Coast	Actinopterygii
Paraliparis valentinae	Indian Ocean Coast	Actinopterygii
Paraluteres arqat	Indian Ocean Coast	Actinopterygii
Paraploactis intonsa	Indian Ocean Coast	Actinopterygii
Paraploactis trachyderma	Indian Ocean Coast	Actinopterygii
Paxton concilians	Indian Ocean Coast	Actinopterygii
Pegasus lancifer	Indian Ocean Coast	Actinopterygii
Penopus microphthalmus	Indian Ocean Coast	Actinopterygii
Peristrominous dolosus	Indian Ocean Coast	Actinopterygii
Photocorynus spiniceps	Indian Ocean Coast	Actinopterygii
Photostomias guernei	Indian Ocean Coast	Actinopterygii
Phyllorhinichthys balushkini	Indian Ocean Coast	Actinopterygii
Platycephalus chauliodous	Indian Ocean Coast	Actinopterygii

**Rare Species**	**System**	**Class**
Plectroglyphidodon randalli	Indian Ocean Coast	Actinopterygii
Polyipnus bruuni	Indian Ocean Coast	Actinopterygii
Pomacentrus limosus	Indian Ocean Coast	Actinopterygii
Porogadus melanocephalus	Indian Ocean Coast	Actinopterygii
Poromitra crassiceps	Indian Ocean Coast	Actinopterygii
Praealticus triangulus	Indian Ocean Coast	Actinopterygii
Pseudamia nigra	Indian Ocean Coast	Actinopterygii
Pseudamia tarri	Indian Ocean Coast	Actinopterygii
Pseudamiops springeri	Indian Ocean Coast	Actinopterygii
Pseudochromis coccinicauda	Indian Ocean Coast	Actinopterygii
Pseudomonacanthus elongatus	Indian Ocean Coast	Actinopterygii
Pseudomyrophis nimius	Indian Ocean Coast	Actinopterygii
Pyramodon lindas	Indian Ocean Coast	Actinopterygii
Rastrelliger brachysoma	Indian Ocean Coast	Actinopterygii
Rhabdoblennius snowi	Indian Ocean Coast	Actinopterygii
Samariscus maculatus	Indian Ocean Coast	Actinopterygii
Sarda sarda	Indian Ocean Coast	Actinopterygii
Sardinella aurita	Indian Ocean Coast	Actinopterygii
Sarpa salpa	Indian Ocean Coast	Actinopterygii
Scomber colias	Indian Ocean Coast	Actinopterygii
Scomberomorus munroi	Indian Ocean Coast	Actinopterygii
Scomberomorus queenslandicus	Indian Ocean Coast	Actinopterygii
Scomberomorus semifasciatus	Indian Ocean Coast	Actinopterygii
Scombrops boops	Indian Ocean Coast	Actinopterygii
Sebastes capensis	Indian Ocean Coast	Actinopterygii
Seriola carpenteri	Indian Ocean Coast	Actinopterygii
Serrivomer schmidti	Indian Ocean Coast	Actinopterygii
Siokunichthys bentuviai	Indian Ocean Coast	Actinopterygii
Siokunichthys southwelli	Indian Ocean Coast	Actinopterygii
Siphamia goreni	Indian Ocean Coast	Actinopterygii
Spectrunculus grandis	Indian Ocean Coast	Actinopterygii
Spondyliosoma cantharus	Indian Ocean Coast	Actinopterygii
Steeneichthys nativitatus	Indian Ocean Coast	Actinopterygii
Stegastes imbricatus	Indian Ocean Coast	Actinopterygii
Stegastes insularis	Indian Ocean Coast	Actinopterygii
Stegastes pelicieri	Indian Ocean Coast	Actinopterygii
Stemonidium hypomelas	Indian Ocean Coast	Actinopterygii
Sthenopus mollis	Indian Ocean Coast	Actinopterygii
Stigmatopora narinosa	Indian Ocean Coast	Actinopterygii
Stolephorus baganensis	Indian Ocean Coast	Actinopterygii
Sudis hyalina	Indian Ocean Coast	Actinopterygii
Sueviota larsonae	Indian Ocean Coast	Actinopterygii
Suezichthys notatus	Indian Ocean Coast	Actinopterygii
Sufflogobius bibarbatus	Indian Ocean Coast	Actinopterygii
Symphurus variegatus	Indian Ocean Coast	Actinopterygii

**Rare Species**	**System**	**Class**
Syngnathus acus	Indian Ocean Coast	Actinopterygii
Taractichthys longipinnis	Indian Ocean Coast	Actinopterygii
Tasmanogobius lasti	Indian Ocean Coast	Actinopterygii
Tetrapturus belone	Indian Ocean Coast	Actinopterygii
Thorogobius rofeni	Indian Ocean Coast	Actinopterygii
Thunnus thynnus	Indian Ocean Coast	Actinopterygii
Torquigener balteus	Indian Ocean Coast	Actinopterygii
Torquigener paxtoni	Indian Ocean Coast	Actinopterygii
Torquigener vicinus	Indian Ocean Coast	Actinopterygii
Torquigener whitleyi	Indian Ocean Coast	Actinopterygii
Trachinotus anak	Indian Ocean Coast	Actinopterygii
Trachinotus ovatus	Indian Ocean Coast	Actinopterygii
Trachurus capensis	Indian Ocean Coast	Actinopterygii
Trachyscorpia carnomagula	Indian Ocean Coast	Actinopterygii
Trigla lyra	Indian Ocean Coast	Actinopterygii
Trimma bisella	Indian Ocean Coast	Actinopterygii
Trimma fucatum	Indian Ocean Coast	Actinopterygii
Trimma sanguinellus	Indian Ocean Coast	Actinopterygii
Trimmatom nanus	Indian Ocean Coast	Actinopterygii
Trimmatom offucius	Indian Ocean Coast	Actinopterygii
Trimmatom sagma	Indian Ocean Coast	Actinopterygii
Trinorfolkia cristata	Indian Ocean Coast	Actinopterygii
Ulua aurochs	Indian Ocean Coast	Actinopterygii
Uranoscopus dahlakensis	Indian Ocean Coast	Actinopterygii
Vanacampus vercoi	Indian Ocean Coast	Actinopterygii
Vinciguerria mabahiss	Indian Ocean Coast	Actinopterygii
Xyelacyba myersi	Indian Ocean Coast	Actinopterygii
Zenarchopterus pappenheimi	Indian Ocean Coast	Actinopterygii
Zenopsis stabilispinosa	Indian Ocean Coast	Actinopterygii
Zeus capensis	Indian Ocean Coast	Actinopterygii
Zu elongatus	Indian Ocean Coast	Actinopterygii
Anacanthobatis marmorata	Indian Ocean Coast	Elasmobranchii
Apristurus microps	Indian Ocean Coast	Elasmobranchii
Bathyraja eatonii	Indian Ocean Coast	Elasmobranchii
Bathyraja hesperafricana	Indian Ocean Coast	Elasmobranchii
Bathyraja irrasa	Indian Ocean Coast	Elasmobranchii
Bathyraja smithii	Indian Ocean Coast	Elasmobranchii
Benthobatis moresbyi	Indian Ocean Coast	Elasmobranchii
Chiloscyllium hasseltii	Indian Ocean Coast	Elasmobranchii
Cruriraja hulleyi	Indian Ocean Coast	Elasmobranchii
Dentiraja flindersi	Indian Ocean Coast	Elasmobranchii
Dentiraja oculata	Indian Ocean Coast	Elasmobranchii
Dipturus canutus	Indian Ocean Coast	Elasmobranchii
Dipturus wengi	Indian Ocean Coast	Elasmobranchii
Hemitrygon parvonigra	Indian Ocean Coast	Elasmobranchii

**Rare Species**	**System**	**Class**
Heteronarce garmani	Indian Ocean Coast	Elasmobranchii
Malacoraja spinacidermis	Indian Ocean Coast	Elasmobranchii
Notoraja sticta	Indian Ocean Coast	Elasmobranchii
Rajella barnardi	Indian Ocean Coast	Elasmobranchii
Rajella challengeri	Indian Ocean Coast	Elasmobranchii
Rajella dissimilis	Indian Ocean Coast	Elasmobranchii
Rajella ravidula	Indian Ocean Coast	Elasmobranchii
Squatina pseudocellata	Indian Ocean Coast	Elasmobranchii
Tetronarce cowleyi	Indian Ocean Coast	Elasmobranchii
Trygonoptera galba	Indian Ocean Coast	Elasmobranchii
Acanthurus leucocheilus	North Atlantic Coast	Actinopterygii
Acanthurus nigricans	North Atlantic Coast	Actinopterygii
Acanthurus tractus	North Atlantic Coast	Actinopterygii
Allocyttus verrucosus	North Atlantic Coast	Actinopterygii
Anchoa chamensis	North Atlantic Coast	Actinopterygii
Anchoa eigenmannia	North Atlantic Coast	Actinopterygii
Anchoa panamensis	North Atlantic Coast	Actinopterygii
Anchoa pectoralis	North Atlantic Coast	Actinopterygii
Antennarius maculatus	North Atlantic Coast	Actinopterygii
Antennarius randalli	North Atlantic Coast	Actinopterygii
Antennatus dorehensis	North Atlantic Coast	Actinopterygii
Antennatus linearis	North Atlantic Coast	Actinopterygii
Antennatus nummifer	North Atlantic Coast	Actinopterygii
Apogon semiornatus	North Atlantic Coast	Actinopterygii
Apogonichthys ocellatus	North Atlantic Coast	Actinopterygii
Apogonichthys perdix	North Atlantic Coast	Actinopterygii
Arothron hispidus	North Atlantic Coast	Actinopterygii
Arothron mappa	North Atlantic Coast	Actinopterygii
Asterropteryx bipunctata	North Atlantic Coast	Actinopterygii
Asterropteryx ensifera	North Atlantic Coast	Actinopterygii
Asterropteryx semipunctata	North Atlantic Coast	Actinopterygii
Ataxolepis henactis	North Atlantic Coast	Actinopterygii
Awaous ocellaris	North Atlantic Coast	Actinopterygii
Bathygobius cocosensis	North Atlantic Coast	Actinopterygii
Bathygobius laddi	North Atlantic Coast	Actinopterygii
Bothrocara elongatum	North Atlantic Coast	Actinopterygii
Brama japonica	North Atlantic Coast	Actinopterygii
Bryaninops tigris	North Atlantic Coast	Actinopterygii
Bryaninops yongei	North Atlantic Coast	Actinopterygii
Buenia massutii	North Atlantic Coast	Actinopterygii
Cabillus lacertops	North Atlantic Coast	Actinopterygii
Callogobius centrolepis	North Atlantic Coast	Actinopterygii
Calotomus spinidens	North Atlantic Coast	Actinopterygii
Cantherhines dumerilii	North Atlantic Coast	Actinopterygii
Cantherhines fronticinctus	North Atlantic Coast	Actinopterygii

Rare Species	System	Class
Canthigaster bennetti	North Atlantic Coast	Actinopterygii
Canthigaster figueiredoi	North Atlantic Coast	Actinopterygii
Canthigaster ocellicincta	North Atlantic Coast	Actinopterygii
Canthigaster solandri	North Atlantic Coast	Actinopterygii
Canthigaster valentini	North Atlantic Coast	Actinopterygii
Caranx melampygus	North Atlantic Coast	Actinopterygii
Careproctus longifilis	North Atlantic Coast	Actinopterygii
Cataetyx simus	North Atlantic Coast	Actinopterygii
Cetoscarus ocellatus	North Atlantic Coast	Actinopterygii
Cheilodipterus isostigmus	North Atlantic Coast	Actinopterygii
Chiasmodon subniger	North Atlantic Coast	Actinopterygii
Chlorophthalmus mento	North Atlantic Coast	Actinopterygii
Choeroichthys brachysoma	North Atlantic Coast	Actinopterygii
Choeroichthys sculptus	North Atlantic Coast	Actinopterygii
Chromis analis	North Atlantic Coast	Actinopterygii
Chromis lepidolepis	North Atlantic Coast	Actinopterygii
Chromis ternatensis	North Atlantic Coast	Actinopterygii
Chromis viridis	North Atlantic Coast	Actinopterygii
Chrysiptera biocellata	North Atlantic Coast	Actinopterygii
Chrysiptera brownriggii	North Atlantic Coast	Actinopterygii
Chrysiptera rollandi	North Atlantic Coast	Actinopterygii
Cirrhilabrus exquisitus	North Atlantic Coast	Actinopterygii
Cirripectes auritus	North Atlantic Coast	Actinopterygii
Cirripectes castaneus	North Atlantic Coast	Actinopterygii
Cirripectes polyzona	North Atlantic Coast	Actinopterygii
Coris dorsomacula	North Atlantic Coast	Actinopterygii
Coryphaenoides anguliceps	North Atlantic Coast	Actinopterygii
Corythoichthys amplexus	North Atlantic Coast	Actinopterygii
Corythoichthys flavofasciatus	North Atlantic Coast	Actinopterygii
Cosmocampus banneri	North Atlantic Coast	Actinopterygii
Cosmocampus maxweberi	North Atlantic Coast	Actinopterygii
Crocodilichthys gracilis	North Atlantic Coast	Actinopterygii
Crossosalarias macrospilus	North Atlantic Coast	Actinopterygii
Cryptocentrus strigilliceps	North Atlantic Coast	Actinopterygii
Ctenogobiops feroculus	North Atlantic Coast	Actinopterygii
Ctenogobiops tangaroai	North Atlantic Coast	Actinopterygii
Cymolutes torquatus	North Atlantic Coast	Actinopterygii
Dactylagnus mundus	North Atlantic Coast	Actinopterygii
Dactyloscopus minutus	North Atlantic Coast	Actinopterygii
Diademichthys lineatus	North Atlantic Coast	Actinopterygii
Dibranchus erinaceus	North Atlantic Coast	Actinopterygii
Dibranchus hystrix	North Atlantic Coast	Actinopterygii
Dibranchus nudivomer	North Atlantic Coast	Actinopterygii
Dibranchus spinosus	North Atlantic Coast	Actinopterygii
Dicrolene filamentosa	North Atlantic Coast	Actinopterygii

**Rare Species**	**System**	**Class**
Diplacanthopoma jordani	North Atlantic Coast	Actinopterygii
Diplecogaster tonstricula	North Atlantic Coast	Actinopterygii
Discordipinna griessingeri	North Atlantic Coast	Actinopterygii
Dunckerocampus dactyliophorus	North Atlantic Coast	Actinopterygii
Echiodon cryomargarites	North Atlantic Coast	Actinopterygii
Ecsenius bicolor	North Atlantic Coast	Actinopterygii
Enchelynassa canina	North Atlantic Coast	Actinopterygii
Enneanectes macrops	North Atlantic Coast	Actinopterygii
Enneapterygius elegans	North Atlantic Coast	Actinopterygii
Enneapterygius flavoccipitis	North Atlantic Coast	Actinopterygii
Enneapterygius fuscoventer	North Atlantic Coast	Actinopterygii
Enneapterygius hemimelas	North Atlantic Coast	Actinopterygii
Enneapterygius minutus	North Atlantic Coast	Actinopterygii
Enneapterygius philippinus	North Atlantic Coast	Actinopterygii
Entomacrodus decussatus	North Atlantic Coast	Actinopterygii
Eviota nebulosa	North Atlantic Coast	Actinopterygii
Eviota prasina	North Atlantic Coast	Actinopterygii
Eviota queenslandica	North Atlantic Coast	Actinopterygii
Eviota sparsa	North Atlantic Coast	Actinopterygii
Eviota storthynx	North Atlantic Coast	Actinopterygii
Exyrias belissimus	North Atlantic Coast	Actinopterygii
Festucalex erythraeus	North Atlantic Coast	Actinopterygii
Fowleria aurita	North Atlantic Coast	Actinopterygii
Fowleria vaiulae	North Atlantic Coast	Actinopterygii
Fowleria variegata	North Atlantic Coast	Actinopterygii
Fusigobius humeralis	North Atlantic Coast	Actinopterygii
Fusigobius signipinnis	North Atlantic Coast	Actinopterygii
Girella stuebeli	North Atlantic Coast	Actinopterygii
Gnatholepis anjerensis	North Atlantic Coast	Actinopterygii
Gnatholepis cauerensis	North Atlantic Coast	Actinopterygii
Gobiodon citrinus	North Atlantic Coast	Actinopterygii
Gobiodon prolixus	North Atlantic Coast	Actinopterygii
Gobiodon rivulatus	North Atlantic Coast	Actinopterygii
Gobiosoma seminudum	North Atlantic Coast	Actinopterygii
Gobulus birdsongi	North Atlantic Coast	Actinopterygii
Gymnapogon philippinus	North Atlantic Coast	Actinopterygii
Gymnapogon urospilotus	North Atlantic Coast	Actinopterygii
Gymnothorax flavimarginatus	North Atlantic Coast	Actinopterygii
Gyrinomimus bruuni	North Atlantic Coast	Actinopterygii
Halicampus mataafae	North Atlantic Coast	Actinopterygii
Halicampus spinirostris	North Atlantic Coast	Actinopterygii
Halichoeres adustus	North Atlantic Coast	Actinopterygii
Halichoeres argus	North Atlantic Coast	Actinopterygii
Halichoeres prosopeion	North Atlantic Coast	Actinopterygii
Himantolophus nigricornis	North Atlantic Coast	Actinopterygii

**Rare Species**	**System**	**Class**
Himantolophus sagamius	North Atlantic Coast	Actinopterygii
Hippichthys spicifer	North Atlantic Coast	Actinopterygii
Hoplostethus mento	North Atlantic Coast	Actinopterygii
Ilyophis arx	North Atlantic Coast	Actinopterygii
Iniistius baldwini	North Atlantic Coast	Actinopterygii
Iniistius pavo	North Atlantic Coast	Actinopterygii
Istigobius decoratus	North Atlantic Coast	Actinopterygii
Istigobius nigroocellatus	North Atlantic Coast	Actinopterygii
Istigobius ornatus	North Atlantic Coast	Actinopterygii
Istiompax indica	North Atlantic Coast	Actinopterygii
Lagocephalus sceleratus	North Atlantic Coast	Actinopterygii
Lepidonectes clarkhubbsi	North Atlantic Coast	Actinopterygii
Luposicya lupus	North Atlantic Coast	Actinopterygii
Lycenchelys callista	North Atlantic Coast	Actinopterygii
Lycenchelys incisa	North Atlantic Coast	Actinopterygii
Lycenchelys jordani	North Atlantic Coast	Actinopterygii
Lycenchelys scaurus	North Atlantic Coast	Actinopterygii
Lycodapus endemoscotus	North Atlantic Coast	Actinopterygii
Lycodes jugoricus	North Atlantic Coast	Actinopterygii
Lycodes sagittarius	North Atlantic Coast	Actinopterygii
Lythrypnus brasiliensis	North Atlantic Coast	Actinopterygii
Macrodontogobius wilburi	North Atlantic Coast	Actinopterygii
Malacoctenus carrowi	North Atlantic Coast	Actinopterygii
Melanocetus eustalus	North Atlantic Coast	Actinopterygii
Melanocetus polyactis	North Atlantic Coast	Actinopterygii
Monomitopus torvus	North Atlantic Coast	Actinopterygii
Mugil thoburni	North Atlantic Coast	Actinopterygii
Mugilogobius notospilus	North Atlantic Coast	Actinopterygii
Nannosalarias nativitatis	North Atlantic Coast	Actinopterygii
Nectamia savayensis	North Atlantic Coast	Actinopterygii
Novaculichthys taeniourus	North Atlantic Coast	Actinopterygii
Novaculoides macrolepidotus	North Atlantic Coast	Actinopterygii
Ogilbia jewettae	North Atlantic Coast	Actinopterygii
Ogilbia robertsoni	North Atlantic Coast	Actinopterygii
Ogilbia sedorae	North Atlantic Coast	Actinopterygii
Oneirodes clarkei	North Atlantic Coast	Actinopterygii
Oneirodes luetkeni	North Atlantic Coast	Actinopterygii
Opostomias micripnus	North Atlantic Coast	Actinopterygii
Ostorhinchus cavitensis	North Atlantic Coast	Actinopterygii
Ostorhinchus cookii	North Atlantic Coast	Actinopterygii
Ostorhinchus nigrofasciatus	North Atlantic Coast	Actinopterygii
Ostorhinchus taeniophorus	North Atlantic Coast	Actinopterygii
Oxycheilinus bimaculatus	North Atlantic Coast	Actinopterygii
Oxycheilinus celebicus	North Atlantic Coast	Actinopterygii
Pachycara crossacanthum	North Atlantic Coast	Actinopterygii

Rare Species	System	Class
Parablennius dialloi	North Atlantic Coast	Actinopterygii
Paragobiodon echinocephalus	North Atlantic Coast	Actinopterygii
Paraliparis cephalus	North Atlantic Coast	Actinopterygii
Paraliparis rosaceus	North Atlantic Coast	Actinopterygii
Paralonchurus peruanus	North Atlantic Coast	Actinopterygii
Paraluteres prionurus	North Atlantic Coast	Actinopterygii
Penetopteryx taeniocephalus	North Atlantic Coast	Actinopterygii
Periophthalmus kalolo	North Atlantic Coast	Actinopterygii
Pervagor janthinosoma	North Atlantic Coast	Actinopterygii
Phoxocampus belcheri	North Atlantic Coast	Actinopterygii
Phoxocampus tetrophthalmus	North Atlantic Coast	Actinopterygii
Plectroglyphidodon leucozonus	North Atlantic Coast	Actinopterygii
Plesiops coeruleolineatus	North Atlantic Coast	Actinopterygii
Plesiops corallicola	North Atlantic Coast	Actinopterygii
Plesiops verecundus	North Atlantic Coast	Actinopterygii
Pleurosicya coerulea	North Atlantic Coast	Actinopterygii
Pleurosicya mossambica	North Atlantic Coast	Actinopterygii
Pleurosicya muscarum	North Atlantic Coast	Actinopterygii
Pleurosicya plicata	North Atlantic Coast	Actinopterygii
Plicomugil labiosus	North Atlantic Coast	Actinopterygii
Pomacentrus amboinensis	North Atlantic Coast	Actinopterygii
Pomacentrus pavo	North Atlantic Coast	Actinopterygii
Pomacentrus philippinus	North Atlantic Coast	Actinopterygii
Pomacentrus tripunctatus	North Atlantic Coast	Actinopterygii
Priolepis cincta	North Atlantic Coast	Actinopterygii
Priolepis semidoliata	North Atlantic Coast	Actinopterygii
Pristiapogon exostigma	North Atlantic Coast	Actinopterygii
Psammogobius biocellatus	North Atlantic Coast	Actinopterygii
Psednos barnardi	North Atlantic Coast	Actinopterygii
Psednos christinae	North Atlantic Coast	Actinopterygii
Pseudamia amblyuroptera	North Atlantic Coast	Actinopterygii
Pseudamia hayashii	North Atlantic Coast	Actinopterygii
Pseudobathylagus milleri	North Atlantic Coast	Actinopterygii
Pseudocheilinus evanidus	North Atlantic Coast	Actinopterygii
Pseudogramma guineensis	North Atlantic Coast	Actinopterygii
Pseudojuloides splendens	North Atlantic Coast	Actinopterygii
Ptereleotris carinata	North Atlantic Coast	Actinopterygii
Redigobius bikolanus	North Atlantic Coast	Actinopterygii
Saccopharynx lavenbergi	North Atlantic Coast	Actinopterygii
Salarias guttatus	North Atlantic Coast	Actinopterygii
Salarias segmentatus	North Atlantic Coast	Actinopterygii
Salarias sinuosus	North Atlantic Coast	Actinopterygii
Sarda orientalis	North Atlantic Coast	Actinopterygii
Scarus festivus	North Atlantic Coast	Actinopterygii
Scarus niger	North Atlantic Coast	Actinopterygii

**Rare Species**	**System**	**Class**
Scarus prasiognathos	North Atlantic Coast	Actinopterygii
Scarus rivulatus	North Atlantic Coast	Actinopterygii
Scarus rubroviolaceus	North Atlantic Coast	Actinopterygii
Scarus tricolor	North Atlantic Coast	Actinopterygii
Scarus trispinosus	North Atlantic Coast	Actinopterygii
Scarus xanthopleura	North Atlantic Coast	Actinopterygii
Scomber japonicus	North Atlantic Coast	Actinopterygii
Siphamia fistulosa	North Atlantic Coast	Actinopterygii
Siphamia tubifer	North Atlantic Coast	Actinopterygii
Stegastes arcifrons	North Atlantic Coast	Actinopterygii
Stegastes beebei	North Atlantic Coast	Actinopterygii
Stegastes leucorus	North Atlantic Coast	Actinopterygii
Stegastes rocasensis	North Atlantic Coast	Actinopterygii
Stenogobius genivittatus	North Atlantic Coast	Actinopterygii
Sternoptyx obscura	North Atlantic Coast	Actinopterygii
Stethojulis bandanensis	North Atlantic Coast	Actinopterygii
Syngnathus auliscus	North Atlantic Coast	Actinopterygii
Taeniamia biguttata	North Atlantic Coast	Actinopterygii
Talismania bifurcata	North Atlantic Coast	Actinopterygii
Tigrigobius janssi	North Atlantic Coast	Actinopterygii
Trachipterus jacksonensis	North Atlantic Coast	Actinopterygii
Trimma benjamini	North Atlantic Coast	Actinopterygii
Trimma flavatrum	North Atlantic Coast	Actinopterygii
Trimma macrophthalmus	North Atlantic Coast	Actinopterygii
Trimma milta	North Atlantic Coast	Actinopterygii
Trimma okinawae	North Atlantic Coast	Actinopterygii
Trimma tevegae	North Atlantic Coast	Actinopterygii
Trinectes xanthurus	North Atlantic Coast	Actinopterygii
Verulux cypselurus	North Atlantic Coast	Actinopterygii
Wetmorella nigropinnata	North Atlantic Coast	Actinopterygii
Xenisthmus polyzonatus	North Atlantic Coast	Actinopterygii
Zenarchopterus dispar	North Atlantic Coast	Actinopterygii
Zoramia leptacantha	North Atlantic Coast	Actinopterygii
Alopias pelagicus	North Atlantic Coast	Elasmobranchii
Bathyraja pallida	North Atlantic Coast	Elasmobranchii
Bathyraja spinosissima	North Atlantic Coast	Elasmobranchii
Carcharhinus amboinensis	North Atlantic Coast	Elasmobranchii
Diplobatis colombiensis	North Atlantic Coast	Elasmobranchii
Diplobatis guamachensis	North Atlantic Coast	Elasmobranchii
Diplobatis ommata	North Atlantic Coast	Elasmobranchii
Etmopterus benchleyi	North Atlantic Coast	Elasmobranchii
Narcine vermiculata	North Atlantic Coast	Elasmobranchii
Neoraja caerulea	North Atlantic Coast	Elasmobranchii
Neoraja carolinensis	North Atlantic Coast	Elasmobranchii
Raja parva	North Atlantic Coast	Elasmobranchii

**Rare Species**	**System**	**Class**
Rhizoprionodon longurio	North Atlantic Coast	Elasmobranchii
Somniosus antarcticus	North Atlantic Coast	Elasmobranchii
Squalus albicaudus	North Atlantic Coast	Elasmobranchii
Triaenodon obesus	North Atlantic Coast	Elasmobranchii
Urotrygon cimar	North Atlantic Coast	Elasmobranchii
Zanobatus maculatus	North Atlantic Coast	Elasmobranchii
Abalistes filamentosus	North East Pacific Coast	Actinopterygii
Acanthurus auranticavus	North East Pacific Coast	Actinopterygii
Acentrogobius cyanomos	North East Pacific Coast	Actinopterygii
Acentrogobius moloanus	North East Pacific Coast	Actinopterygii
Acentronura breviperula	North East Pacific Coast	Actinopterygii
Acentronura tentaculata	North East Pacific Coast	Actinopterygii
Adventor elongatus	North East Pacific Coast	Actinopterygii
Aldrovandia rostrata	North East Pacific Coast	Actinopterygii
Alepocephalus owstoni	North East Pacific Coast	Actinopterygii
Allips concolor	North East Pacific Coast	Actinopterygii
Amblygobius decussatus	North East Pacific Coast	Actinopterygii
Amblyotrypauchen arctocephalus	North East Pacific Coast	Actinopterygii
Amioides polyacanthus	North East Pacific Coast	Actinopterygii
Anchoa chamensis	North East Pacific Coast	Actinopterygii
Anchoa panamensis	North East Pacific Coast	Actinopterygii
Apagesoma delosommatus	North East Pacific Coast	Actinopterygii
Aphyonus gelatinosus	North East Pacific Coast	Actinopterygii
Apocryptodon madurensis	North East Pacific Coast	Actinopterygii
Apogon seminigracaudus	North East Pacific Coast	Actinopterygii
Apogonichthyoides atripes	North East Pacific Coast	Actinopterygii
Apogonichthyoides heptastygma	North East Pacific Coast	Actinopterygii
Arotrolepis filicauda	North East Pacific Coast	Actinopterygii
Axoclinus multicinctus	North East Pacific Coast	Actinopterygii
Barathrites parri	North East Pacific Coast	Actinopterygii
Barathrodemus manatinus	North East Pacific Coast	Actinopterygii
Barathronus maculatus	North East Pacific Coast	Actinopterygii
Barbuligobius boehlkei	North East Pacific Coast	Actinopterygii
Bassogigas gillii	North East Pacific Coast	Actinopterygii
Bassozetus elongatus	North East Pacific Coast	Actinopterygii
Bassozetus galatheae	North East Pacific Coast	Actinopterygii
Bassozetus glutinosus	North East Pacific Coast	Actinopterygii
Bassozetus normalis	North East Pacific Coast	Actinopterygii
Bassozetus taenia	North East Pacific Coast	Actinopterygii
Bathyaploactis ornatissima	North East Pacific Coast	Actinopterygii
Bathygadus favosus	North East Pacific Coast	Actinopterygii
Bathygadus melanobranchus	North East Pacific Coast	Actinopterygii
Bathygobius petrophilus	North East Pacific Coast	Actinopterygii
Bathymaster derjugini	North East Pacific Coast	Actinopterygii
Bathyonus laticeps	North East Pacific Coast	Actinopterygii

**Rare Species**	**System**	**Class**
Bathypterois grallator	North East Pacific Coast	Actinopterygii
Bathytroctes macrolepis	North East Pacific Coast	Actinopterygii
Bathytyphlops sewelli	North East Pacific Coast	Actinopterygii
Boleophthalmus birdsongi	North East Pacific Coast	Actinopterygii
Borostomias pacificus	North East Pacific Coast	Actinopterygii
Brachaluteres taylori	North East Pacific Coast	Actinopterygii
Brevoortia gunteri	North East Pacific Coast	Actinopterygii
Brinkmannella elongata	North East Pacific Coast	Actinopterygii
Brotulotaenia crassa	North East Pacific Coast	Actinopterygii
Bryaninops annella	North East Pacific Coast	Actinopterygii
Bryaninops isis	North East Pacific Coast	Actinopterygii
Bythites gerdae	North East Pacific Coast	Actinopterygii
Cabillus macrophthalmus	North East Pacific Coast	Actinopterygii
Callogobius centrolepis	North East Pacific Coast	Actinopterygii
Campichthys tricarinatus	North East Pacific Coast	Actinopterygii
Canthigaster tyleri	North East Pacific Coast	Actinopterygii
Cetomimus teevani	North East Pacific Coast	Actinopterygii
Chascanopsetta lugubris	North East Pacific Coast	Actinopterygii
Cheilinus undulatus	North East Pacific Coast	Actinopterygii
Chiasmodon niger	North East Pacific Coast	Actinopterygii
Chlorurus bleekeri	North East Pacific Coast	Actinopterygii
Chlorurus oedema	North East Pacific Coast	Actinopterygii
Chrionema chlorotaenia	North East Pacific Coast	Actinopterygii
Chromis struhsakeri	North East Pacific Coast	Actinopterygii
Chrysiptera rex	North East Pacific Coast	Actinopterygii
Chrysiptera traceyi	North East Pacific Coast	Actinopterygii
Chrysiptera unimaculata	North East Pacific Coast	Actinopterygii
Coelorinchus occa	North East Pacific Coast	Actinopterygii
Conidens samoensis	North East Pacific Coast	Actinopterygii
Conocara nigrum	North East Pacific Coast	Actinopterygii
Coryphaenoides ariommus	North East Pacific Coast	Actinopterygii
Coryphaenoides fernandezianus	North East Pacific Coast	Actinopterygii
Corythoichthys haematopterus	North East Pacific Coast	Actinopterygii
Cosmocampus darrosanus	North East Pacific Coast	Actinopterygii
Cosmocampus investigatoris	North East Pacific Coast	Actinopterygii
Dibranchus tremendus	North East Pacific Coast	Actinopterygii
Dicrolene introniger	North East Pacific Coast	Actinopterygii
Dicrolene kanazawai	North East Pacific Coast	Actinopterygii
Dischistodus melanotus	North East Pacific Coast	Actinopterygii
Dorosoma petenense	North East Pacific Coast	Actinopterygii
Doryrhamphus janssi	North East Pacific Coast	Actinopterygii
Drombus halei	North East Pacific Coast	Actinopterygii
Drombus ocyurus	North East Pacific Coast	Actinopterygii
Drombus triangularis	North East Pacific Coast	Actinopterygii
Ecsenius lineatus	North East Pacific Coast	Actinopterygii

Rare Species	System	Class
Encheliophis sagamianus	North East Pacific Coast	Actinopterygii
Enneapterygius gracilis	North East Pacific Coast	Actinopterygii
Enneapterygius larsonae	North East Pacific Coast	Actinopterygii
Enneapterygius mirabilis	North East Pacific Coast	Actinopterygii
Epigonus telescopus	North East Pacific Coast	Actinopterygii
Etelis radiosus	North East Pacific Coast	Actinopterygii
Etrumeus sadina	North East Pacific Coast	Actinopterygii
Eugnathogobius mindora	North East Pacific Coast	Actinopterygii
Eumegistus brevorti	North East Pacific Coast	Actinopterygii
Eustomias filifer	North East Pacific Coast	Actinopterygii
Eustomias parri	North East Pacific Coast	Actinopterygii
Evermannichthys spongicola	North East Pacific Coast	Actinopterygii
Eviota bipunctata	North East Pacific Coast	Actinopterygii
Eviota latifasciata	North East Pacific Coast	Actinopterygii
Eviota spilota	North East Pacific Coast	Actinopterygii
Festucalex gibbsi	North East Pacific Coast	Actinopterygii
Festucalex kulbickii	North East Pacific Coast	Actinopterygii
Fowleria flammea	North East Pacific Coast	Actinopterygii
Gadella molokaiensis	North East Pacific Coast	Actinopterygii
Glossogobius giuris	North East Pacific Coast	Actinopterygii
Gobiodon erythrospilus	North East Pacific Coast	Actinopterygii
Gobiodon fulvus	North East Pacific Coast	Actinopterygii
Gobiodon okinawae	North East Pacific Coast	Actinopterygii
Gobiodon quinquestrigatus	North East Pacific Coast	Actinopterygii
Gobiodon unicolor	North East Pacific Coast	Actinopterygii
Gobiopsis exigua	North East Pacific Coast	Actinopterygii
Gobiopsis malekulae	North East Pacific Coast	Actinopterygii
Gobiopterus mindanensis	North East Pacific Coast	Actinopterygii
Gobius bontii	North East Pacific Coast	Actinopterygii
Grammatorcynus bilineatus	North East Pacific Coast	Actinopterygii
Grammatostomias dentatus	North East Pacific Coast	Actinopterygii
Grammonus robustus	North East Pacific Coast	Actinopterygii
Gymnapogon annona	North East Pacific Coast	Actinopterygii
Gymnothorax favagineus	North East Pacific Coast	Actinopterygii
Gymnothorax pseudoherrei	North East Pacific Coast	Actinopterygii
Halargyreus johnsonii	North East Pacific Coast	Actinopterygii
Halicampus brocki	North East Pacific Coast	Actinopterygii
Halicampus macrorhynchus	North East Pacific Coast	Actinopterygii
Halicampus nitidus	North East Pacific Coast	Actinopterygii
Halicmetus niger	North East Pacific Coast	Actinopterygii
Haptenchelys texis	North East Pacific Coast	Actinopterygii
Harpadon translucens	North East Pacific Coast	Actinopterygii
Helcogramma gymnauchen	North East Pacific Coast	Actinopterygii
Helcogramma nigra	North East Pacific Coast	Actinopterygii
Helcogramma obtusirostris	North East Pacific Coast	Actinopterygii

Rare Species	System	Class
Helcogramma rhinoceros	North East Pacific Coast	Actinopterygii
Helicolenus avius	North East Pacific Coast	Actinopterygii
Helicolenus dactylopterus	North East Pacific Coast	Actinopterygii
Helicolenus fedorovi	North East Pacific Coast	Actinopterygii
Helotes sexlineatus	North East Pacific Coast	Actinopterygii
Hippichthys cyanospilos	North East Pacific Coast	Actinopterygii
Hippichthys penicillus	North East Pacific Coast	Actinopterygii
Hippocampus alatus	North East Pacific Coast	Actinopterygii
Hippocampus colemani	North East Pacific Coast	Actinopterygii
Hippocampus grandiceps	North East Pacific Coast	Actinopterygii
Hippocampus hendriki	North East Pacific Coast	Actinopterygii
Hippocampus kelloggi	North East Pacific Coast	Actinopterygii
Hippocampus multispinus	North East Pacific Coast	Actinopterygii
Hippocampus zosterae	North East Pacific Coast	Actinopterygii
Holcomycteronus squamosus	North East Pacific Coast	Actinopterygii
Hoplostethus atlanticus	North East Pacific Coast	Actinopterygii
Hozukius guyotensis	North East Pacific Coast	Actinopterygii
Hypopleuron caninum	North East Pacific Coast	Actinopterygii
Ilisha lunula	North East Pacific Coast	Actinopterygii
Iniistius auropunctatus	North East Pacific Coast	Actinopterygii
Ipnops murrayi	North East Pacific Coast	Actinopterygii
Istiblennius dussumieri	North East Pacific Coast	Actinopterygii
Jaydia argyrogaster	North East Pacific Coast	Actinopterygii
Kyonemichthys rumengani	North East Pacific Coast	Actinopterygii
Lepophidium kallion	North East Pacific Coast	Actinopterygii
Leptostomias longibarba	North East Pacific Coast	Actinopterygii
Lestidiops similis	North East Pacific Coast	Actinopterygii
Leucicorus atlanticus	North East Pacific Coast	Actinopterygii
Luciobrotula corethromycter	North East Pacific Coast	Actinopterygii
Lycenchelys bullisi	North East Pacific Coast	Actinopterygii
Lycenchelys parini	North East Pacific Coast	Actinopterygii
Lycenchelys remissaria	North East Pacific Coast	Actinopterygii
Lycenchelys ryukyuensis	North East Pacific Coast	Actinopterygii
Lycenchelys tohokuensis	North East Pacific Coast	Actinopterygii
Lycodapus microchir	North East Pacific Coast	Actinopterygii
Lycodes soldatovi	North East Pacific Coast	Actinopterygii
Lythrypnus insularis	North East Pacific Coast	Actinopterygii
Mesopristes argenteus	North East Pacific Coast	Actinopterygii
Micrognathus natans	North East Pacific Coast	Actinopterygii
Mixomyrophis pusillipinna	North East Pacific Coast	Actinopterygii
Monognathus ahlstromi	North East Pacific Coast	Actinopterygii
Moolgarda pedaraki	North East Pacific Coast	Actinopterygii
Mugilogobius mertoni	North East Pacific Coast	Actinopterygii
Muraenichthys thompsoni	North East Pacific Coast	Actinopterygii
Nannocampus pictus	North East Pacific Coast	Actinopterygii

**Rare Species**	**System**	**Class**
Naso fageni	North East Pacific Coast	Actinopterygii
Nybelinella erikssoni	North East Pacific Coast	Actinopterygii
Omobranchus elongatus	North East Pacific Coast	Actinopterygii
Omobranchus rotundiceps	North East Pacific Coast	Actinopterygii
Oneirodes carlsbergi	North East Pacific Coast	Actinopterygii
Oneirodes macrosteus	North East Pacific Coast	Actinopterygii
Oneirodes melanocauda	North East Pacific Coast	Actinopterygii
Oneirodes thysanema	North East Pacific Coast	Actinopterygii
Osteomugil cunnesius	North East Pacific Coast	Actinopterygii
Ostorhinchus gularis	North East Pacific Coast	Actinopterygii
Ostorhinchus holotaenia	North East Pacific Coast	Actinopterygii
Oxyurichthys cornutus	North East Pacific Coast	Actinopterygii
Oxyurichthys uronema	North East Pacific Coast	Actinopterygii
Pagrus pagrus	North East Pacific Coast	Actinopterygii
Palatogobius grandoculus	North East Pacific Coast	Actinopterygii
Parabathymyrus oregoni	North East Pacific Coast	Actinopterygii
Paracentropogon vespa	North East Pacific Coast	Actinopterygii
Paragobiodon melanosoma	North East Pacific Coast	Actinopterygii
Paragobiodon modestus	North East Pacific Coast	Actinopterygii
Paraliparis penicillus	North East Pacific Coast	Actinopterygii
Paramonacanthus curtorhynchos	North East Pacific Coast	Actinopterygii
Paramonacanthus pusillus	North East Pacific Coast	Actinopterygii
Paramonacanthus tricuspis	North East Pacific Coast	Actinopterygii
Parioglossus rainfordi	North East Pacific Coast	Actinopterygii
Parioglossus taeniatus	North East Pacific Coast	Actinopterygii
Pellona harroweri	North East Pacific Coast	Actinopterygii
Penopus microphthalmus	North East Pacific Coast	Actinopterygii
Peprilus crenulatus	North East Pacific Coast	Actinopterygii
Photonectes margarita	North East Pacific Coast	Actinopterygii
Phyllorhinichthys balushkini	North East Pacific Coast	Actinopterygii
Pleurosicya prognatha	North East Pacific Coast	Actinopterygii
Polyacanthonotus africanus	North East Pacific Coast	Actinopterygii
Polyipnus aquavitus	North East Pacific Coast	Actinopterygii
Pomacentrus bankanensis	North East Pacific Coast	Actinopterygii
Pomacentrus chrysurus	North East Pacific Coast	Actinopterygii
Pomacentrus grammorhynchus	North East Pacific Coast	Actinopterygii
Pomacentrus limosus	North East Pacific Coast	Actinopterygii
Pomacentrus milleri	North East Pacific Coast	Actinopterygii
Pomacentrus moluccensis	North East Pacific Coast	Actinopterygii
Pomacentrus nagasakiensis	North East Pacific Coast	Actinopterygii
Pomacentrus taeniometopon	North East Pacific Coast	Actinopterygii
Pomacentrus trilineatus	North East Pacific Coast	Actinopterygii
Pomadasys trifasciatus	North East Pacific Coast	Actinopterygii
Pomatomus saltatrix	North East Pacific Coast	Actinopterygii
Pontinus tentacularis	North East Pacific Coast	Actinopterygii

Rare Species	System	Class
Porogadus catena	North East Pacific Coast	Actinopterygii
Porogadus silus	North East Pacific Coast	Actinopterygii
Poromitra cristiceps	North East Pacific Coast	Actinopterygii
Priolepis agrena	North East Pacific Coast	Actinopterygii
Priolepis profunda	North East Pacific Coast	Actinopterygii
Pseudamia nigra	North East Pacific Coast	Actinopterygii
Pseudogobius melanostictus	North East Pacific Coast	Actinopterygii
Pseudomonacanthus elongatus	North East Pacific Coast	Actinopterygii
Pycnocraspedum armatum	North East Pacific Coast	Actinopterygii
Pyrolycus manusanus	North East Pacific Coast	Actinopterygii
Redigobius balteatus	North East Pacific Coast	Actinopterygii
Rudarius excelsus	North East Pacific Coast	Actinopterygii
Saccogaster staigeri	North East Pacific Coast	Actinopterygii
Salarias ramosus	North East Pacific Coast	Actinopterygii
Sarda chiliensis	North East Pacific Coast	Actinopterygii
Scarus dimidiatus	North East Pacific Coast	Actinopterygii
Scarus ferrugineus	North East Pacific Coast	Actinopterygii
Scarus quoyi	North East Pacific Coast	Actinopterygii
Scarus viridifucatus	North East Pacific Coast	Actinopterygii
Sebastes exsul	North East Pacific Coast	Actinopterygii
Sebastes flammeus	North East Pacific Coast	Actinopterygii
Sebastes sinensis	North East Pacific Coast	Actinopterygii
Siganus canaliculatus	North East Pacific Coast	Actinopterygii
Siganus punctatissimus	North East Pacific Coast	Actinopterygii
Siokunichthys herrei	North East Pacific Coast	Actinopterygii
Siphamia guttulata	North East Pacific Coast	Actinopterygii
Siphamia majimai	North East Pacific Coast	Actinopterygii
Siphamia tubulata	North East Pacific Coast	Actinopterygii
Solegnathus hardwickii	North East Pacific Coast	Actinopterygii
Solegnathus lettiensis	North East Pacific Coast	Actinopterygii
Sonoda megalophthalma	North East Pacific Coast	Actinopterygii
Stalix histrio	North East Pacific Coast	Actinopterygii
Stegastes obreptus	North East Pacific Coast	Actinopterygii
Stephanoberyx monae	North East Pacific Coast	Actinopterygii
Stichaeus grigorjewi	North East Pacific Coast	Actinopterygii
Strongylura strongylura	North East Pacific Coast	Actinopterygii
Sueviota larsonae	North East Pacific Coast	Actinopterygii
Syngnathus insulae	North East Pacific Coast	Actinopterygii
Tetrapturus georgii	North East Pacific Coast	Actinopterygii
Thamnaconus striatus	North East Pacific Coast	Actinopterygii
Thaumatichthys binghami	North East Pacific Coast	Actinopterygii
Thunnus thynnus	North East Pacific Coast	Actinopterygii
Torquigener parcuspinus	North East Pacific Coast	Actinopterygii
Torquigener whitleyi	North East Pacific Coast	Actinopterygii
Trachinotus anak	North East Pacific Coast	Actinopterygii

**Rare Species**	**System**	**Class**
Trachyrhamphus bicoarctatus	North East Pacific Coast	Actinopterygii
Trachyrhamphus longirostris	North East Pacific Coast	Actinopterygii
Trimma flammeum	North East Pacific Coast	Actinopterygii
Trimma hoesei	North East Pacific Coast	Actinopterygii
Trimma hollemani	North East Pacific Coast	Actinopterygii
Trimma lantana	North East Pacific Coast	Actinopterygii
Trimma nomurai	North East Pacific Coast	Actinopterygii
Trimma pentherum	North East Pacific Coast	Actinopterygii
Trimma readerae	North East Pacific Coast	Actinopterygii
Trimma sheppardi	North East Pacific Coast	Actinopterygii
Trimmatom nanus	North East Pacific Coast	Actinopterygii
Trimmatom pharus	North East Pacific Coast	Actinopterygii
Tyson belos	North East Pacific Coast	Actinopterygii
Urophycis cirrata	North East Pacific Coast	Actinopterygii
Vanderhorstia longimanus	North East Pacific Coast	Actinopterygii
Varicus marilynae	North East Pacific Coast	Actinopterygii
Xenodermichthys nodulosus	North East Pacific Coast	Actinopterygii
Xenomystax austrinus	North East Pacific Coast	Actinopterygii
Zenarchopterus rasori	North East Pacific Coast	Actinopterygii
Zenopsis stabilispinosa	North East Pacific Coast	Actinopterygii
Zesticelus bathybius	North East Pacific Coast	Actinopterygii
Aetomylaeus caeruleofasciatus	North East Pacific Coast	Elasmobranchii
Aetomylaeus milvus	North East Pacific Coast	Elasmobranchii
Carcharhinus sorrah	North East Pacific Coast	Elasmobranchii
Hemigaleus australiensis	North East Pacific Coast	Elasmobranchii
Himantura leoparda	North East Pacific Coast	Elasmobranchii
Narcine brunnea	North East Pacific Coast	Elasmobranchii
Neotrygon picta	North East Pacific Coast	Elasmobranchii
Rhizoprionodon oligolinx	North East Pacific Coast	Elasmobranchii
Acanthoplesiops psilogaster	North West Pacific Coast	Actinopterygii
Acipenser mikadoi	North West Pacific Coast	Actinopterygii
Acipenser sinensis	North West Pacific Coast	Actinopterygii
Adventor elongatus	North West Pacific Coast	Actinopterygii
Aldrovandia phalacra	North West Pacific Coast	Actinopterygii
Alepocephalus owstoni	North West Pacific Coast	Actinopterygii
Alepocephalus tenebrosus	North West Pacific Coast	Actinopterygii
Alepocephalus umbriceps	North West Pacific Coast	Actinopterygii
Allocareproctus jordani	North West Pacific Coast	Actinopterygii
Allocareproctus tanix	North West Pacific Coast	Actinopterygii
Andamia amphibius	North West Pacific Coast	Actinopterygii
Andriashevia aptera	North West Pacific Coast	Actinopterygii
Anguilla bengalensis	North West Pacific Coast	Actinopterygii
Anoplarchus purpurescens	North West Pacific Coast	Actinopterygii
Aphyonus gelatinosus	North West Pacific Coast	Actinopterygii
Apogonichthyoides uninotatus	North West Pacific Coast	Actinopterygii

**Rare Species**	**System**	**Class**
Ariosoma obud	North West Pacific Coast	Actinopterygii
Arius microcephalus	North West Pacific Coast	Actinopterygii
Arius oetik	North West Pacific Coast	Actinopterygii
Arothron carduus	North West Pacific Coast	Actinopterygii
Arotrolepis filicauda	North West Pacific Coast	Actinopterygii
Artediellus ingens	North West Pacific Coast	Actinopterygii
Artediellus scaber	North West Pacific Coast	Actinopterygii
Astrocottus leprops	North West Pacific Coast	Actinopterygii
Astronesthes indopacificus	North West Pacific Coast	Actinopterygii
Atheresthes stomias	North West Pacific Coast	Actinopterygii
Atractoscion aequidens	North West Pacific Coast	Actinopterygii
Barathrodemus nasutus	North West Pacific Coast	Actinopterygii
Barathronus maculatus	North West Pacific Coast	Actinopterygii
Bascanichthys myersi	North West Pacific Coast	Actinopterygii
Bassogigas gillii	North West Pacific Coast	Actinopterygii
Bathophilus vaillanti	North West Pacific Coast	Actinopterygii
Bathycongrus macrocercus	North West Pacific Coast	Actinopterygii
Bathycongrus nasicus	North West Pacific Coast	Actinopterygii
Bathylaco nigricans	North West Pacific Coast	Actinopterygii
Bathymaster derjugini	North West Pacific Coast	Actinopterygii
Bathytroctes zugmayeri	North West Pacific Coast	Actinopterygii
Bellottia galatheae	North West Pacific Coast	Actinopterygii
Benthalbella dentata	North West Pacific Coast	Actinopterygii
Benthodesmus neglectus	North West Pacific Coast	Actinopterygii
Benthodesmus suluensis	North West Pacific Coast	Actinopterygii
Bhanotia nuda	North West Pacific Coast	Actinopterygii
Bhanotia pauciradiata	North West Pacific Coast	Actinopterygii
Bilabria ornata	North West Pacific Coast	Actinopterygii
Blepsias bilobus	North West Pacific Coast	Actinopterygii
Borostomias pacificus	North West Pacific Coast	Actinopterygii
Brotulotaenia crassa	North West Pacific Coast	Actinopterygii
Bryaninops earlei	North West Pacific Coast	Actinopterygii
Careproctus attenuatus	North West Pacific Coast	Actinopterygii
Careproctus curilanus	North West Pacific Coast	Actinopterygii
Careproctus cyclocephalus	North West Pacific Coast	Actinopterygii
Careproctus homopterus	North West Pacific Coast	Actinopterygii
Careproctus macrodiscus	North West Pacific Coast	Actinopterygii
Careproctus mollis	North West Pacific Coast	Actinopterygii
Careproctus pycnosoma	North West Pacific Coast	Actinopterygii
Careproctus rhodomelas	North West Pacific Coast	Actinopterygii
Careproctus roseofuscus	North West Pacific Coast	Actinopterygii
Cataetyx platyrhynchus	North West Pacific Coast	Actinopterygii
Centrodraco atrifilum	North West Pacific Coast	Actinopterygii
Cetonurichthys subinflatus	North West Pacific Coast	Actinopterygii
Chascanopsetta prognatha	North West Pacific Coast	Actinopterygii

Rare Species	System	Class
Chaunax pictus	North West Pacific Coast	Actinopterygii
Chaunax reticulatus	North West Pacific Coast	Actinopterygii
Chirolophis decoratus	North West Pacific Coast	Actinopterygii
Choeroichthys suillus	North West Pacific Coast	Actinopterygii
Chrionema furunoi	North West Pacific Coast	Actinopterygii
Cirrhilabrus joanallenae	North West Pacific Coast	Actinopterygii
Cirripectes jenningsi	North West Pacific Coast	Actinopterygii
Cirripectes kuwamurai	North West Pacific Coast	Actinopterygii
Coelorinchus dorsalis	North West Pacific Coast	Actinopterygii
Coelorinchus japonicus	North West Pacific Coast	Actinopterygii
Coelorinchus kermadecus	North West Pacific Coast	Actinopterygii
Coelorinchus macrochir	North West Pacific Coast	Actinopterygii
Coelorinchus macrolepis	North West Pacific Coast	Actinopterygii
Coelorinchus parallelus	North West Pacific Coast	Actinopterygii
Coelorinchus quadricristatus	North West Pacific Coast	Actinopterygii
Coelorinchus quincunciatus	North West Pacific Coast	Actinopterygii
Coelorinchus sheni	North West Pacific Coast	Actinopterygii
Coelorinchus weberi	North West Pacific Coast	Actinopterygii
Conidens samoensis	North West Pacific Coast	Actinopterygii
Coryphaenoides dubius	North West Pacific Coast	Actinopterygii
Coryphaenoides orthogrammus	North West Pacific Coast	Actinopterygii
Coryphaenoides rudis	North West Pacific Coast	Actinopterygii
Coryphaenoides woodmasoni	North West Pacific Coast	Actinopterygii
Corythoichthys conspicillatus	North West Pacific Coast	Actinopterygii
Crenimugil heterocheilos	North West Pacific Coast	Actinopterygii
Cryptacanthodes aleutensis	North West Pacific Coast	Actinopterygii
Cyclopteropsis brashnikowi	North West Pacific Coast	Actinopterygii
Davidijordania poecilimon	North West Pacific Coast	Actinopterygii
Diagramma labiosum	North West Pacific Coast	Actinopterygii
Diancistrus eremitus	North West Pacific Coast	Actinopterygii
Diaphus drachmanni	North West Pacific Coast	Actinopterygii
Diaphus wisneri	North West Pacific Coast	Actinopterygii
Dibranchus hystrix	North West Pacific Coast	Actinopterygii
Dibranchus nasutus	North West Pacific Coast	Actinopterygii
Diceratias pileatus	North West Pacific Coast	Actinopterygii
Dicrolene nigricaudis	North West Pacific Coast	Actinopterygii
Diplophos pacificus	North West Pacific Coast	Actinopterygii
Drombus halei	North West Pacific Coast	Actinopterygii
Drombus palackyi	North West Pacific Coast	Actinopterygii
Ebinania vermiculata	North West Pacific Coast	Actinopterygii
Ecsenius dilemma	North West Pacific Coast	Actinopterygii
Ecsenius fijiensis	North West Pacific Coast	Actinopterygii
Ecsenius isos	North West Pacific Coast	Actinopterygii
Ecsenius oculus	North West Pacific Coast	Actinopterygii
Ecsenius ops	North West Pacific Coast	Actinopterygii

Rare Species	System	Class
Ecsenius pardus	North West Pacific Coast	Actinopterygii
Ecsenius portenoyi	North West Pacific Coast	Actinopterygii
Ecsenius tessera	North West Pacific Coast	Actinopterygii
Elassodiscus obscurus	North West Pacific Coast	Actinopterygii
Enigmacanthus filamentosus	North West Pacific Coast	Actinopterygii
Enneapterygius cheni	North West Pacific Coast	Actinopterygii
Enneapterygius pusillus	North West Pacific Coast	Actinopterygii
Entomacrodus vermiculatus	North West Pacific Coast	Actinopterygii
Entomacrodus williamsi	North West Pacific Coast	Actinopterygii
Ereunias grallator	North West Pacific Coast	Actinopterygii
Eubalichthys caeruleoguttatus	North West Pacific Coast	Actinopterygii
Eugnathogobius variegatus	North West Pacific Coast	Actinopterygii
Eumicrotremus barbatus	North West Pacific Coast	Actinopterygii
Eumicrotremus derjugini	North West Pacific Coast	Actinopterygii
Eumicrotremus fedorovi	North West Pacific Coast	Actinopterygii
Eumicrotremus schmidti	North West Pacific Coast	Actinopterygii
Eumicrotremus soldatovi	North West Pacific Coast	Actinopterygii
Eumicrotremus taranetzi	North West Pacific Coast	Actinopterygii
Eurypharynx pelecanoides	North West Pacific Coast	Actinopterygii
Eusurculus andamanensis	North West Pacific Coast	Actinopterygii
Eviota epistigmata	North West Pacific Coast	Actinopterygii
Eviota filamentosa	North West Pacific Coast	Actinopterygii
Eviota flebilis	North West Pacific Coast	Actinopterygii
Eviota karaspila	North West Pacific Coast	Actinopterygii
Eviota lachdeberei	North West Pacific Coast	Actinopterygii
Eviota latifasciata	North West Pacific Coast	Actinopterygii
Eviota maculibotella	North West Pacific Coast	Actinopterygii
Eviota mimica	North West Pacific Coast	Actinopterygii
Eviota pinocchioi	North West Pacific Coast	Actinopterygii
Eviota richardi	North West Pacific Coast	Actinopterygii
Eviota rubriceps	North West Pacific Coast	Actinopterygii
Eviota rubriguttata	North West Pacific Coast	Actinopterygii
Eviota rubrimaculata	North West Pacific Coast	Actinopterygii
Eviota shibukawai	North West Pacific Coast	Actinopterygii
Eviota singula	North West Pacific Coast	Actinopterygii
Eviota specca	North West Pacific Coast	Actinopterygii
Eviota teresae	North West Pacific Coast	Actinopterygii
Eviota thamani	North West Pacific Coast	Actinopterygii
Eviota winterbottomi	North West Pacific Coast	Actinopterygii
Evoxymetopon taeniatus	North West Pacific Coast	Actinopterygii
Feia dabra	North West Pacific Coast	Actinopterygii
Festucalex rufus	North West Pacific Coast	Actinopterygii
Festucalex wassi	North West Pacific Coast	Actinopterygii
Gavialiceps taeniola	North West Pacific Coast	Actinopterygii
Gavialiceps taiwanensis	North West Pacific Coast	Actinopterygii

**Rare Species**	**System**	**Class**
Gigantactis kreffti	North West Pacific Coast	Actinopterygii
Gigantactis watermani	North West Pacific Coast	Actinopterygii
Gulaphallus panayensis	North West Pacific Coast	Actinopterygii
Gymnelopsis brevifenestrata	North West Pacific Coast	Actinopterygii
Gymnelus pauciporus	North West Pacific Coast	Actinopterygii
Gymnelus popovi	North West Pacific Coast	Actinopterygii
Hadropareia middendorffii	North West Pacific Coast	Actinopterygii
Halargyreus johnsonii	North West Pacific Coast	Actinopterygii
Halosaurus carinicauda	North West Pacific Coast	Actinopterygii
Halosaurus pectoralis	North West Pacific Coast	Actinopterygii
Hastatobythites arafurensis	North West Pacific Coast	Actinopterygii
Helcogramma cerasina	North West Pacific Coast	Actinopterygii
Helcogramma lacuna	North West Pacific Coast	Actinopterygii
Helcogramma nesion	North West Pacific Coast	Actinopterygii
Helicolenus fedorovi	North West Pacific Coast	Actinopterygii
Hemigobius mingi	North West Pacific Coast	Actinopterygii
Hemilepidotus jordani	North West Pacific Coast	Actinopterygii
Hemilepidotus zapus	North West Pacific Coast	Actinopterygii
Herwigia kreffti	North West Pacific Coast	Actinopterygii
Hetereleotris exilis	North West Pacific Coast	Actinopterygii
Himantolophus borealis	North West Pacific Coast	Actinopterygii
Hippocampus bargibanti	North West Pacific Coast	Actinopterygii
Hippocampus colemani	North West Pacific Coast	Actinopterygii
Hippocampus coronatus	North West Pacific Coast	Actinopterygii
Hippocampus grandiceps	North West Pacific Coast	Actinopterygii
Hippocampus hendriki	North West Pacific Coast	Actinopterygii
Hippocampus japapigu	North West Pacific Coast	Actinopterygii
Hippocampus sindonis	North West Pacific Coast	Actinopterygii
Hirundichthys indicus	North West Pacific Coast	Actinopterygii
Holcomycteronus pterotus	North West Pacific Coast	Actinopterygii
Hoplichthys prosemion	North West Pacific Coast	Actinopterygii
Icelus armatus	North West Pacific Coast	Actinopterygii
Icelus euryops	North West Pacific Coast	Actinopterygii
Ijimaia dofleini	North West Pacific Coast	Actinopterygii
Ilyophis nigeli	North West Pacific Coast	Actinopterygii
Iniistius griffithsi	North West Pacific Coast	Actinopterygii
Istiblennius colei	North West Pacific Coast	Actinopterygii
Japonoconger sivicolus	North West Pacific Coast	Actinopterygii
Japonolycodes abei	North West Pacific Coast	Actinopterygii
Jaydia melanopus	North West Pacific Coast	Actinopterygii
Kali indica	North West Pacific Coast	Actinopterygii
Lachneratus phasmaticus	North West Pacific Coast	Actinopterygii
Laemonema filodorsale	North West Pacific Coast	Actinopterygii
Lampanyctus hubbsi	North West Pacific Coast	Actinopterygii
Lepidotrigla russelli	North West Pacific Coast	Actinopterygii

Rare Species	System	Class
Leptostichaeus pumilus	North West Pacific Coast	Actinopterygii
Leptostomias multifilis	North West Pacific Coast	Actinopterygii
Lestidiops ringens	North West Pacific Coast	Actinopterygii
Lestidium nudum	North West Pacific Coast	Actinopterygii
Lethotremus awae	North West Pacific Coast	Actinopterygii
Liopsetta glacialis	North West Pacific Coast	Actinopterygii
Liparis pulchellus	North West Pacific Coast	Actinopterygii
Lophodolos acanthognathus	North West Pacific Coast	Actinopterygii
Lumpenella longirostris	North West Pacific Coast	Actinopterygii
Lumpenus sagitta	North West Pacific Coast	Actinopterygii
Lycenchelys albomaculata	North West Pacific Coast	Actinopterygii
Lycenchelys alta	North West Pacific Coast	Actinopterygii
Lycenchelys aurantiaca	North West Pacific Coast	Actinopterygii
Lycenchelys crotalinus	North West Pacific Coast	Actinopterygii
Lycenchelys makushok	North West Pacific Coast	Actinopterygii
Lycenchelys melanostomias	North West Pacific Coast	Actinopterygii
Lycenchelys remissaria	North West Pacific Coast	Actinopterygii
Lycenchelys rosea	North West Pacific Coast	Actinopterygii
Lycenchelys ryukyuensis	North West Pacific Coast	Actinopterygii
Lycenchelys squamosa	North West Pacific Coast	Actinopterygii
Lycenchelys tohokuensis	North West Pacific Coast	Actinopterygii
Lycenchelys vitiazi	North West Pacific Coast	Actinopterygii
Lycodapus derjugini	North West Pacific Coast	Actinopterygii
Lycodes caudimaculatus	North West Pacific Coast	Actinopterygii
Lycodes japonicus	North West Pacific Coast	Actinopterygii
Lycodes jenseni	North West Pacific Coast	Actinopterygii
Lycodes jugoricus	North West Pacific Coast	Actinopterygii
Lycodes marisalbi	North West Pacific Coast	Actinopterygii
Lycodes microlepidotus	North West Pacific Coast	Actinopterygii
Lycodes mucosus	North West Pacific Coast	Actinopterygii
Lycodes nakamurae	North West Pacific Coast	Actinopterygii
Lycodes ocellatus	North West Pacific Coast	Actinopterygii
Lycodes paucilepidotus	North West Pacific Coast	Actinopterygii
Lycodes pectoralis	North West Pacific Coast	Actinopterygii
Lycodes soldatovi	North West Pacific Coast	Actinopterygii
Magadanichthys skopetsi	North West Pacific Coast	Actinopterygii
Malacocottus gibber	North West Pacific Coast	Actinopterygii
Malacosteus australis	North West Pacific Coast	Actinopterygii
Malthopsis asperata	North West Pacific Coast	Actinopterygii
Malthopsis parva	North West Pacific Coast	Actinopterygii
Margrethia valentinae	North West Pacific Coast	Actinopterygii
Meadia roseni	North West Pacific Coast	Actinopterygii
Melanostigma orientale	North West Pacific Coast	Actinopterygii
Melanostomias niger	North West Pacific Coast	Actinopterygii
Mephisto fraserbrunneri	North West Pacific Coast	Actinopterygii

Rare Species	System	Class
Microphotolepis schmidti	North West Pacific Coast	Actinopterygii
Microstomus pacificus	North West Pacific Coast	Actinopterygii
Microstomus shuntovi	North West Pacific Coast	Actinopterygii
Monomitopus conjugator	North West Pacific Coast	Actinopterygii
Monomitopus nigripinnis	North West Pacific Coast	Actinopterygii
Mora moro	North West Pacific Coast	Actinopterygii
Myoxocephalus scorpioides	North West Pacific Coast	Actinopterygii
Nannobrachium indicum	North West Pacific Coast	Actinopterygii
Nansenia candida	North West Pacific Coast	Actinopterygii
Narcetes stomias	North West Pacific Coast	Actinopterygii
Nemapteryx macronotacantha	North West Pacific Coast	Actinopterygii
Neobythites bimaculatus	North West Pacific Coast	Actinopterygii
Neobythites fijiensis	North West Pacific Coast	Actinopterygii
Neobythites pallidus	North West Pacific Coast	Actinopterygii
Neostethus villadolidi	North West Pacific Coast	Actinopterygii
Obliquogobius fluvostriatus	North West Pacific Coast	Actinopterygii
Oncorhynchus mykiss	North West Pacific Coast	Actinopterygii
Oneirodes alius	North West Pacific Coast	Actinopterygii
Oneirodes cristatus	North West Pacific Coast	Actinopterygii
Oneirodes micronema	North West Pacific Coast	Actinopterygii
Oneirodes schmidti	North West Pacific Coast	Actinopterygii
Opaeophacus acrogeneius	North West Pacific Coast	Actinopterygii
Ophichthus aphotistos	North West Pacific Coast	Actinopterygii
Ophichthus exourus	North West Pacific Coast	Actinopterygii
Ophichthus microstictus	North West Pacific Coast	Actinopterygii
Ostorhinchus cyanotaenia	North West Pacific Coast	Actinopterygii
Palmoliparis beckeri	North West Pacific Coast	Actinopterygii
Pandaka pusilla	North West Pacific Coast	Actinopterygii
Paracheilinus rubricaudalis	North West Pacific Coast	Actinopterygii
Paragobiodon kasaii	North West Pacific Coast	Actinopterygii
Parahucho perryi	North West Pacific Coast	Actinopterygii
Paraliparis albeolus	North West Pacific Coast	Actinopterygii
Paraliparis entochloris	North West Pacific Coast	Actinopterygii
Paraliparis holomelas	North West Pacific Coast	Actinopterygii
Parascolopsis melanophrys	North West Pacific Coast	Actinopterygii
Parkraemeria saltator	North West Pacific Coast	Actinopterygii
Pentaceros wheeleri	North West Pacific Coast	Actinopterygii
Petroscirtes lupus	North West Pacific Coast	Actinopterygii
Petroscirtes springeri	North West Pacific Coast	Actinopterygii
Petroscirtes thepassii	North West Pacific Coast	Actinopterygii
Phallostethus lehi	North West Pacific Coast	Actinopterygii
Pholidapus dybowskii	North West Pacific Coast	Actinopterygii
Photostomias guernei	North West Pacific Coast	Actinopterygii
Phoxacromion kaneharai	North West Pacific Coast	Actinopterygii
Physiculus japonicus	North West Pacific Coast	Actinopterygii

Rare Species	System	Class
Phytichthys chirus	North West Pacific Coast	Actinopterygii
Platytroctes mirus	North West Pacific Coast	Actinopterygii
Polyipnus ovatus	North West Pacific Coast	Actinopterygii
Polyipnus tridentifer	North West Pacific Coast	Actinopterygii
Pomacentrus albimaculus	North West Pacific Coast	Actinopterygii
Pomacentrus bintanensis	North West Pacific Coast	Actinopterygii
Pomacentrus callainus	North West Pacific Coast	Actinopterygii
Pomacentrus limosus	North West Pacific Coast	Actinopterygii
Pomacentrus microspilus	North West Pacific Coast	Actinopterygii
Pomacentrus wardi	North West Pacific Coast	Actinopterygii
Porocottus camtschaticus	North West Pacific Coast	Actinopterygii
Poromitra crassa	North West Pacific Coast	Actinopterygii
Poromitra cristiceps	North West Pacific Coast	Actinopterygii
Poromitra glochidiata	North West Pacific Coast	Actinopterygii
Praealticus tanegasimae	North West Pacific Coast	Actinopterygii
Praealticus triangulus	North West Pacific Coast	Actinopterygii
Priolepis triops	North West Pacific Coast	Actinopterygii
Priolepis vexilla	North West Pacific Coast	Actinopterygii
Prionurus scalprum	North West Pacific Coast	Actinopterygii
Pseudocaranx dentex	North West Pacific Coast	Actinopterygii
Pseudoliparis amblystomopsis	North West Pacific Coast	Actinopterygii
Pseudomonacanthus elongatus	North West Pacific Coast	Actinopterygii
Pseudonezumia cetonuropsis	North West Pacific Coast	Actinopterygii
Pseudonezumia japonicus	North West Pacific Coast	Actinopterygii
Pterygotrigla pauli	North West Pacific Coast	Actinopterygii
Puck pinnata	North West Pacific Coast	Actinopterygii
Puzanovia rubra	North West Pacific Coast	Actinopterygii
Pyramodon lindas	North West Pacific Coast	Actinopterygii
Randallichthys filamentosus	North West Pacific Coast	Actinopterygii
Redigobius isognathus	North West Pacific Coast	Actinopterygii
Rexea nakamurai	North West Pacific Coast	Actinopterygii
Rouleina danae	North West Pacific Coast	Actinopterygii
Salarias luctuosus	North West Pacific Coast	Actinopterygii
Salarias sexfilum	North West Pacific Coast	Actinopterygii
Salvelinus alpinus	North West Pacific Coast	Actinopterygii
Saurenchelys lateromaculatus	North West Pacific Coast	Actinopterygii
Schindleria brevipinguis	North West Pacific Coast	Actinopterygii
Schindleria pietschmanni	North West Pacific Coast	Actinopterygii
Scopelarchus stephensi	North West Pacific Coast	Actinopterygii
Sebastes baramenuke	North West Pacific Coast	Actinopterygii
Sebastes ciliatus	North West Pacific Coast	Actinopterygii
Sebastes crameri	North West Pacific Coast	Actinopterygii
Sebastes flammeus	North West Pacific Coast	Actinopterygii
Sebastes hubbsi	North West Pacific Coast	Actinopterygii
Sebastes ijimae	North West Pacific Coast	Actinopterygii

**Rare Species**	**System**	**Class**
Sebastes inermis	North West Pacific Coast	Actinopterygii
Sebastes itinus	North West Pacific Coast	Actinopterygii
Sebastes longispinis	North West Pacific Coast	Actinopterygii
Sebastes matsubarai	North West Pacific Coast	Actinopterygii
Sebastes melanops	North West Pacific Coast	Actinopterygii
Sebastes minor	North West Pacific Coast	Actinopterygii
Sebastes mystinus	North West Pacific Coast	Actinopterygii
Sebastes nivosus	North West Pacific Coast	Actinopterygii
Sebastes oblongus	North West Pacific Coast	Actinopterygii
Sebastes pachycephalus	North West Pacific Coast	Actinopterygii
Sebastes proriger	North West Pacific Coast	Actinopterygii
Sebastes scythropus	North West Pacific Coast	Actinopterygii
Sebastes thompsoni	North West Pacific Coast	Actinopterygii
Sebastes trivittatus	North West Pacific Coast	Actinopterygii
Sebastes variegatus	North West Pacific Coast	Actinopterygii
Sebastes ventricosus	North West Pacific Coast	Actinopterygii
Sebastes vulpes	North West Pacific Coast	Actinopterygii
Sebastes wakiyai	North West Pacific Coast	Actinopterygii
Sebastes zacentrus	North West Pacific Coast	Actinopterygii
Sicyopterus japonicus	North West Pacific Coast	Actinopterygii
Sicyopus zosterophorus	North West Pacific Coast	Actinopterygii
Siokunichthys bentuviai	North West Pacific Coast	Actinopterygii
Siokunichthys southwelli	North West Pacific Coast	Actinopterygii
Siphamia cuneiceps	North West Pacific Coast	Actinopterygii
Siphamia guttulata	North West Pacific Coast	Actinopterygii
Siphamia senoui	North West Pacific Coast	Actinopterygii
Stanulus talboti	North West Pacific Coast	Actinopterygii
Stegastes altus	North West Pacific Coast	Actinopterygii
Stegastes aureus	North West Pacific Coast	Actinopterygii
Stegastes insularis	North West Pacific Coast	Actinopterygii
Stephanolepis cirrhifer	North West Pacific Coast	Actinopterygii
Stereolepis doederleini	North West Pacific Coast	Actinopterygii
Stichaeus nozawae	North West Pacific Coast	Actinopterygii
Stichaeus ochriamkini	North West Pacific Coast	Actinopterygii
Sueviota larsonae	North West Pacific Coast	Actinopterygii
Suezichthys notatus	North West Pacific Coast	Actinopterygii
Symphurus regani	North West Pacific Coast	Actinopterygii
Symphurus thermophilus	North West Pacific Coast	Actinopterygii
Taenioides eruptionis	North West Pacific Coast	Actinopterygii
Temnocora candida	North West Pacific Coast	Actinopterygii
Tenualosa ilisha	North West Pacific Coast	Actinopterygii
Thamnaconus fijiensis	North West Pacific Coast	Actinopterygii
Thorophos nexilis	North West Pacific Coast	Actinopterygii
Torquigener whitleyi	North West Pacific Coast	Actinopterygii
Trachinotus anak	North West Pacific Coast	Actinopterygii

Rare Species	System	Class
Trachonurus yiwardaus	North West Pacific Coast	Actinopterygii
Trachurus japonicus	North West Pacific Coast	Actinopterygii
Tribolodon sachalinensis	North West Pacific Coast	Actinopterygii
Triglops dorothy	North West Pacific Coast	Actinopterygii
Trimma anthrenum	North West Pacific Coast	Actinopterygii
Trimma aturirii	North West Pacific Coast	Actinopterygii
Trimma burridgeae	North West Pacific Coast	Actinopterygii
Trimma fasciatum	North West Pacific Coast	Actinopterygii
Trimma habrum	North West Pacific Coast	Actinopterygii
Trimma hamartium	North West Pacific Coast	Actinopterygii
Trimma hotsarihiensis	North West Pacific Coast	Actinopterygii
Trimma imaii	North West Pacific Coast	Actinopterygii
Trimma kardium	North West Pacific Coast	Actinopterygii
Trimma pentherum	North West Pacific Coast	Actinopterygii
Trimma tauroculum	North West Pacific Coast	Actinopterygii
Trimma trioculatum	North West Pacific Coast	Actinopterygii
Trimmatom nanus	North West Pacific Coast	Actinopterygii
Tryssogobius flavolineatus	North West Pacific Coast	Actinopterygii
Tryssogobius porosus	North West Pacific Coast	Actinopterygii
Ulua aurochs	North West Pacific Coast	Actinopterygii
Ungusurculus williamsi	North West Pacific Coast	Actinopterygii
Vanderhorstia nannai	North West Pacific Coast	Actinopterygii
Venefica tentaculata	North West Pacific Coast	Actinopterygii
Zenopsis stabilispinosa	North West Pacific Coast	Actinopterygii
Zesticelus bathybius	North West Pacific Coast	Actinopterygii
Zesticelus ochotensis	North West Pacific Coast	Actinopterygii
Zoramia flebila	North West Pacific Coast	Actinopterygii
Apristurus fedorovi	North West Pacific Coast	Elasmobranchii
Apristurus gibbosus	North West Pacific Coast	Elasmobranchii
Apristurus macrorhynchus	North West Pacific Coast	Elasmobranchii
Bathyraja maculata	North West Pacific Coast	Elasmobranchii
Cephaloscyllium cooki	North West Pacific Coast	Elasmobranchii
Cephaloscyllium signourum	North West Pacific Coast	Elasmobranchii
Chiloscyllium hasseltii	North West Pacific Coast	Elasmobranchii
Galeus corriganae	North West Pacific Coast	Elasmobranchii
Hemiscyllium halmahera	North West Pacific Coast	Elasmobranchii
Narcine maculata	North West Pacific Coast	Elasmobranchii
Narcinops ornata	North West Pacific Coast	Elasmobranchii
Neotrygon picta	North West Pacific Coast	Elasmobranchii
Okamejei schmidti	North West Pacific Coast	Elasmobranchii
Rhinobatos manai	North West Pacific Coast	Elasmobranchii
Rhinoraja kujiensis	North West Pacific Coast	Elasmobranchii
Urogymnus lobistoma	North West Pacific Coast	Elasmobranchii
Urolophus deforgesi	North West Pacific Coast	Elasmobranchii
Acyrtus lanthanum	South Atlantic Coast	Actinopterygii

**Rare Species**	**System**	**Class**
Aiakas kreffti	South Atlantic Coast	Actinopterygii
Akko dionaea	South Atlantic Coast	Actinopterygii
Aluterus monoceros	South Atlantic Coast	Actinopterygii
Amblyrhynchotes honckenii	South Atlantic Coast	Actinopterygii
Anchoviella brevirostris	South Atlantic Coast	Actinopterygii
Anguilla mossambica	South Atlantic Coast	Actinopterygii
Antennablennius bifilum	South Atlantic Coast	Actinopterygii
Antennarius pardalis	South Atlantic Coast	Actinopterygii
Antennatus nummifer	South Atlantic Coast	Actinopterygii
Aphanopus mikhailini	South Atlantic Coast	Actinopterygii
Apogon imberbis	South Atlantic Coast	Actinopterygii
Aulostomus strigosus	South Atlantic Coast	Actinopterygii
Bassozetus levistomatus	South Atlantic Coast	Actinopterygii
Bassozetus zenkevitchi	South Atlantic Coast	Actinopterygii
Bathyonus caudalis	South Atlantic Coast	Actinopterygii
Benthodesmus elongatus	South Atlantic Coast	Actinopterygii
Bollmannia communis	South Atlantic Coast	Actinopterygii
Bothrocara molle	South Atlantic Coast	Actinopterygii
Brama japonica	South Atlantic Coast	Actinopterygii
Brevoortia pectinata	South Atlantic Coast	Actinopterygii
Bryx randalli	South Atlantic Coast	Actinopterygii
Caffrogobius caffer	South Atlantic Coast	Actinopterygii
Calamopteryx robinsorum	South Atlantic Coast	Actinopterygii
Cantherhines pardalis	South Atlantic Coast	Actinopterygii
Canthigaster solandri	South Atlantic Coast	Actinopterygii
Chelon tricuspidens	South Atlantic Coast	Actinopterygii
Chelonodon laticeps	South Atlantic Coast	Actinopterygii
Chriolepis vespa	South Atlantic Coast	Actinopterygii
Chromis cadenati	South Atlantic Coast	Actinopterygii
Chromis chromis	South Atlantic Coast	Actinopterygii
Chromis limbata	South Atlantic Coast	Actinopterygii
Corcyrogobius lubbocki	South Atlantic Coast	Actinopterygii
Coryogalops william	South Atlantic Coast	Actinopterygii
Coryphopterus kuna	South Atlantic Coast	Actinopterygii
Cosmocampus profundus	South Atlantic Coast	Actinopterygii
Cryothenia peninsulae	South Atlantic Coast	Actinopterygii
Ctenogobius phenacus	South Atlantic Coast	Actinopterygii
Decapterus macarellus	South Atlantic Coast	Actinopterygii
Discordipinna filamentosa	South Atlantic Coast	Actinopterygii
Elacatinus chancei	South Atlantic Coast	Actinopterygii
Elacatinus lori	South Atlantic Coast	Actinopterygii
Elagatis bipinnulata	South Atlantic Coast	Actinopterygii
Emblemaria australis	South Atlantic Coast	Actinopterygii
Emblemariopsis pricei	South Atlantic Coast	Actinopterygii
Engraulis capensis	South Atlantic Coast	Actinopterygii

Rare Species	System	Class
Entomacrodus textilis	South Atlantic Coast	Actinopterygii
Ethmalosa fimbriata	South Atlantic Coast	Actinopterygii
Evermannichthys bicolor	South Atlantic Coast	Actinopterygii
Evermannichthys spongicola	South Atlantic Coast	Actinopterygii
Girella stuebeli	South Atlantic Coast	Actinopterygii
Girella zonata	South Atlantic Coast	Actinopterygii
Glossogobius giuris	South Atlantic Coast	Actinopterygii
Gnatholepis thompsoni	South Atlantic Coast	Actinopterygii
Gobiesox adustus	South Atlantic Coast	Actinopterygii
Gobioclinus bucciferus	South Atlantic Coast	Actinopterygii
Gobiosoma hemigymnum	South Atlantic Coast	Actinopterygii
Gorogobius nigricinctus	South Atlantic Coast	Actinopterygii
Grammonus longhursti	South Atlantic Coast	Actinopterygii
Gymnothorax favagineus	South Atlantic Coast	Actinopterygii
Gymnothorax nudivomer	South Atlantic Coast	Actinopterygii
Haemulon squamipinna	South Atlantic Coast	Actinopterygii
Halichoeres garnoti	South Atlantic Coast	Actinopterygii
Halichoeres sazimai	South Atlantic Coast	Actinopterygii
Helcogramma obtusirostris	South Atlantic Coast	Actinopterygii
Helicolenus mouchezi	South Atlantic Coast	Actinopterygii
Hippocampus algiricus	South Atlantic Coast	Actinopterygii
Hippocampus patagonicus	South Atlantic Coast	Actinopterygii
Hippocampus reidi	South Atlantic Coast	Actinopterygii
Holcomycteronus profundissimus	South Atlantic Coast	Actinopterygii
Hypleurochilus springeri	South Atlantic Coast	Actinopterygii
Hyporhamphus picarti	South Atlantic Coast	Actinopterygii
Ilisha africana	South Atlantic Coast	Actinopterygii
Istiompax indica	South Atlantic Coast	Actinopterygii
Kajikia audax	South Atlantic Coast	Actinopterygii
Lachnolaimus maximus	South Atlantic Coast	Actinopterygii
Lagocephalus inermis	South Atlantic Coast	Actinopterygii
Lagocephalus lunaris	South Atlantic Coast	Actinopterygii
Leucicorus atlanticus	South Atlantic Coast	Actinopterygii
Lutjanus agennes	South Atlantic Coast	Actinopterygii
Lutjanus buccanella	South Atlantic Coast	Actinopterygii
Lutjanus campechanus	South Atlantic Coast	Actinopterygii
Lutjanus dentatus	South Atlantic Coast	Actinopterygii
Lycenchelys argentina	South Atlantic Coast	Actinopterygii
Lycenchelys bachmanni	South Atlantic Coast	Actinopterygii
Lycenchelys bullisi	South Atlantic Coast	Actinopterygii
Lycodes terraenovae	South Atlantic Coast	Actinopterygii
Lycodonus vermiformis	South Atlantic Coast	Actinopterygii
Macruronus novaezelandiae	South Atlantic Coast	Actinopterygii
Membras vagrans	South Atlantic Coast	Actinopterygii
Micrognathus erugatus	South Atlantic Coast	Actinopterygii

**Rare Species**	**System**	**Class**
Microspathodon frontatus	South Atlantic Coast	Actinopterygii
Moolgarda pedaraki	South Atlantic Coast	Actinopterygii
Mugil incilis	South Atlantic Coast	Actinopterygii
Myrophis anterodorsalis	South Atlantic Coast	Actinopterygii
Nerophis maculatus	South Atlantic Coast	Actinopterygii
Notacanthus abbotti	South Atlantic Coast	Actinopterygii
Nybelinella erikssoni	South Atlantic Coast	Actinopterygii
Ogilbia jeffwilliamsi	South Atlantic Coast	Actinopterygii
Ogilbia mccoskeri	South Atlantic Coast	Actinopterygii
Ogilbia tyleri	South Atlantic Coast	Actinopterygii
Ogilbichthys ferocis	South Atlantic Coast	Actinopterygii
Ogilbichthys kakuki	South Atlantic Coast	Actinopterygii
Ophidion guianense	South Atlantic Coast	Actinopterygii
Osteomugil robustus	South Atlantic Coast	Actinopterygii
Ostracion cubicus	South Atlantic Coast	Actinopterygii
Oxyurichthys microlepis	South Atlantic Coast	Actinopterygii
Pachycara caribbaeum	South Atlantic Coast	Actinopterygii
Pachycara crossacanthum	South Atlantic Coast	Actinopterygii
Pachycara thermophilum	South Atlantic Coast	Actinopterygii
Paedovaricus imswe	South Atlantic Coast	Actinopterygii
Palatogobius grandoculus	South Atlantic Coast	Actinopterygii
Parablennius dialloi	South Atlantic Coast	Actinopterygii
Parablennius sierraensis	South Atlantic Coast	Actinopterygii
Parablennius verryckeni	South Atlantic Coast	Actinopterygii
Parachaenichthys georgianus	South Atlantic Coast	Actinopterygii
Paraclinus arcanus	South Atlantic Coast	Actinopterygii
Paraclinus fasciatus	South Atlantic Coast	Actinopterygii
Paraclinus spectator	South Atlantic Coast	Actinopterygii
Paramonacanthus pusillus	South Atlantic Coast	Actinopterygii
Pellonula leonensis	South Atlantic Coast	Actinopterygii
Piedrabuenia ringueleti	South Atlantic Coast	Actinopterygii
Platygillellus brasiliensis	South Atlantic Coast	Actinopterygii
Plectorhinchus macrolepis	South Atlantic Coast	Actinopterygii
Plectroglyphidodon leucozonus	South Atlantic Coast	Actinopterygii
Plectrypops lima	South Atlantic Coast	Actinopterygii
Pomadasys maculatus	South Atlantic Coast	Actinopterygii
Porogobius schlegelii	South Atlantic Coast	Actinopterygii
Prionurus biafraensis	South Atlantic Coast	Actinopterygii
Psilotris kaufmani	South Atlantic Coast	Actinopterygii
Rhinecanthus aculeatus	South Atlantic Coast	Actinopterygii
Saccogaster tuberculata	South Atlantic Coast	Actinopterygii
Sanopus splendidus	South Atlantic Coast	Actinopterygii
Sargocentron caudimaculatum	South Atlantic Coast	Actinopterygii
Sarpa salpa	South Atlantic Coast	Actinopterygii
Scartella emarginata	South Atlantic Coast	Actinopterygii

**Rare Species**	**System**	**Class**
Scartella poiti	South Atlantic Coast	Actinopterygii
Scarus hoefleri	South Atlantic Coast	Actinopterygii
Sciades parkeri	South Atlantic Coast	Actinopterygii
Sciades proops	South Atlantic Coast	Actinopterygii
Sciadonus jonassoni	South Atlantic Coast	Actinopterygii
Scomberomorus commerson	South Atlantic Coast	Actinopterygii
Scombrops boops	South Atlantic Coast	Actinopterygii
Sebastes capensis	South Atlantic Coast	Actinopterygii
Secutor insidiator	South Atlantic Coast	Actinopterygii
Secutor ruconius	South Atlantic Coast	Actinopterygii
Seleniolycus laevifasciatus	South Atlantic Coast	Actinopterygii
Seriola rivoliana	South Atlantic Coast	Actinopterygii
Solenostomus cyanopterus	South Atlantic Coast	Actinopterygii
Sphoeroides marmoratus	South Atlantic Coast	Actinopterygii
Sphyraena qenie	South Atlantic Coast	Actinopterygii
Starksia culebrae	South Atlantic Coast	Actinopterygii
Starksia fasciata	South Atlantic Coast	Actinopterygii
Starksia occidentalis	South Atlantic Coast	Actinopterygii
Starksia variabilis	South Atlantic Coast	Actinopterygii
Stegastes otophorus	South Atlantic Coast	Actinopterygii
Stegastes punctatus	South Atlantic Coast	Actinopterygii
Stegastes rocasensis	South Atlantic Coast	Actinopterygii
Stephanolepis auratus	South Atlantic Coast	Actinopterygii
Storrsia olsoni	South Atlantic Coast	Actinopterygii
Strongylura senegalensis	South Atlantic Coast	Actinopterygii
Syngnathoides biaculeatus	South Atlantic Coast	Actinopterygii
Syngnathus acus	South Atlantic Coast	Actinopterygii
Thalassoma purpureum	South Atlantic Coast	Actinopterygii
Thamnaconus modestoides	South Atlantic Coast	Actinopterygii
Thorogobius alvheimi	South Atlantic Coast	Actinopterygii
Thorogobius laureatus	South Atlantic Coast	Actinopterygii
Thorogobius rofeni	South Atlantic Coast	Actinopterygii
Tomicodon leurodiscus	South Atlantic Coast	Actinopterygii
Torquigener hypselogeneion	South Atlantic Coast	Actinopterygii
Trachinotus africanus	South Atlantic Coast	Actinopterygii
Trachinotus maxillosus	South Atlantic Coast	Actinopterygii
Trachinotus teraia	South Atlantic Coast	Actinopterygii
Vanneaugobius canariensis	South Atlantic Coast	Actinopterygii
Varicus marilynae	South Atlantic Coast	Actinopterygii
Vladichthys gloverensis	South Atlantic Coast	Actinopterygii
Volodichthys herwigi	South Atlantic Coast	Actinopterygii
Wheelerigobius maltzani	South Atlantic Coast	Actinopterygii
Carcharhinus amboinensis	South Atlantic Coast	Elasmobranchii
Cruriraja hulleyi	South Atlantic Coast	Elasmobranchii
Leucoraja compagnoi	South Atlantic Coast	Elasmobranchii

Rare Species	System	Class
Neoraja africana	South Atlantic Coast	Elasmobranchii
Raja parva	South Atlantic Coast	Elasmobranchii
Rhizoprionodon acutus	South Atlantic Coast	Elasmobranchii
Tetronarce cowleyi	South Atlantic Coast	Elasmobranchii
Urotrygon microphthalmum	South Atlantic Coast	Elasmobranchii
Zanobatus maculatus	South Atlantic Coast	Elasmobranchii
Abalistes filamentosus	South East Pacific Coast	Actinopterygii
Acentronura breviperula	South East Pacific Coast	Actinopterygii
Acreichthys radiatus	South East Pacific Coast	Actinopterygii
Amblyeleotris ogasawarensis	South East Pacific Coast	Actinopterygii
Amblyeleotris rubrimarginata	South East Pacific Coast	Actinopterygii
Amblygobius cheraphilus	South East Pacific Coast	Actinopterygii
Amblygobius decussatus	South East Pacific Coast	Actinopterygii
Anchoa nasus	South East Pacific Coast	Actinopterygii
Andamia amphibius	South East Pacific Coast	Actinopterygii
Anguilla dieffenbachii	South East Pacific Coast	Actinopterygii
Antennatus linearis	South East Pacific Coast	Actinopterygii
Apogon dammermani	South East Pacific Coast	Actinopterygii
Apogon guadalupensis	South East Pacific Coast	Actinopterygii
Apogon marquesensis	South East Pacific Coast	Actinopterygii
Apogon seminigracaudus	South East Pacific Coast	Actinopterygii
Apopterygion oculus	South East Pacific Coast	Actinopterygii
Atrosalarias holomelas	South East Pacific Coast	Actinopterygii
Barathronus maculatus	South East Pacific Coast	Actinopterygii
Bathygobius lineatus	South East Pacific Coast	Actinopterygii
Bathyphylax pruvosti	South East Pacific Coast	Actinopterygii
Cabillus macrophthalmus	South East Pacific Coast	Actinopterygii
Callionymus brevianalis	South East Pacific Coast	Actinopterygii
Callionymus marquesensis	South East Pacific Coast	Actinopterygii
Calotomus zonarchus	South East Pacific Coast	Actinopterygii
Campichthys tricarinatus	South East Pacific Coast	Actinopterygii
Cantherhines verecundus	South East Pacific Coast	Actinopterygii
Canthigaster callisterna	South East Pacific Coast	Actinopterygii
Careproctus ampliceps	South East Pacific Coast	Actinopterygii
Careproctus vladibeckeri	South East Pacific Coast	Actinopterygii
Chaunax latipunctatus	South East Pacific Coast	Actinopterygii
Chloroscombrus orqueta	South East Pacific Coast	Actinopterygii
Chlorurus bleekeri	South East Pacific Coast	Actinopterygii
Chromis dispilus	South East Pacific Coast	Actinopterygii
Chrysiptera rapanui	South East Pacific Coast	Actinopterygii
Chrysiptera rex	South East Pacific Coast	Actinopterygii
Chrysiptera unimaculata	South East Pacific Coast	Actinopterygii
Cirripectes perustus	South East Pacific Coast	Actinopterygii
Coelophrys oblonga	South East Pacific Coast	Actinopterygii
Coelorinchus commutabilis	South East Pacific Coast	Actinopterygii

**Rare Species**	**System**	**Class**
Conocara nigrum	South East Pacific Coast	Actinopterygii
Contusus richei	South East Pacific Coast	Actinopterygii
Coryphaenoides ferrieri	South East Pacific Coast	Actinopterygii
Cosmocampus heraldi	South East Pacific Coast	Actinopterygii
Crenimugil buchanani	South East Pacific Coast	Actinopterygii
Crocodilichthys gracilis	South East Pacific Coast	Actinopterygii
Ctenochaetus tominiensis	South East Pacific Coast	Actinopterygii
Ctenogobiops mitodes	South East Pacific Coast	Actinopterygii
Dactylagnus mundus	South East Pacific Coast	Actinopterygii
Dieidolycus leptodermatus	South East Pacific Coast	Actinopterygii
Doryrhamphus negrosensis	South East Pacific Coast	Actinopterygii
Dunckerocampus chapmani	South East Pacific Coast	Actinopterygii
Ecsenius fijiensis	South East Pacific Coast	Actinopterygii
Ecsenius pardus	South East Pacific Coast	Actinopterygii
Elops affinis	South East Pacific Coast	Actinopterygii
Enneanectes reticulatus	South East Pacific Coast	Actinopterygii
Enneapterygius atriceps	South East Pacific Coast	Actinopterygii
Enneapterygius mirabilis	South East Pacific Coast	Actinopterygii
Enneapterygius rubicauda	South East Pacific Coast	Actinopterygii
Eviota bipunctata	South East Pacific Coast	Actinopterygii
Eviota hoesei	South East Pacific Coast	Actinopterygii
Eviota karaspila	South East Pacific Coast	Actinopterygii
Eviota kermadecensis	South East Pacific Coast	Actinopterygii
Eviota lacrimosa	South East Pacific Coast	Actinopterygii
Eviota mimica	South East Pacific Coast	Actinopterygii
Eviota punctulata	South East Pacific Coast	Actinopterygii
Eviota queenslandica	South East Pacific Coast	Actinopterygii
Eviota richardi	South East Pacific Coast	Actinopterygii
Eviota spilota	South East Pacific Coast	Actinopterygii
Eviota storthynx	South East Pacific Coast	Actinopterygii
Eviota teresae	South East Pacific Coast	Actinopterygii
Eviota thamani	South East Pacific Coast	Actinopterygii
Exerpes asper	South East Pacific Coast	Actinopterygii
Festucalex gibbsi	South East Pacific Coast	Actinopterygii
Festucalex kulbickii	South East Pacific Coast	Actinopterygii
Fowlerichthys avalonis	South East Pacific Coast	Actinopterygii
Fusigobius gracilis	South East Pacific Coast	Actinopterygii
Girella albostriata	South East Pacific Coast	Actinopterygii
Girella cyanea	South East Pacific Coast	Actinopterygii
Girella fimbriata	South East Pacific Coast	Actinopterygii
Girella freminvillii	South East Pacific Coast	Actinopterygii
Glossogobius celebius	South East Pacific Coast	Actinopterygii
Gnatholepis knighti	South East Pacific Coast	Actinopterygii
Gobiodon erythrospilus	South East Pacific Coast	Actinopterygii
Gobiodon multilineatus	South East Pacific Coast	Actinopterygii

Rare Species	System	Class
Gobiodon okinawae	South East Pacific Coast	Actinopterygii
Gobiodon unicolor	South East Pacific Coast	Actinopterygii
Gobiopsis atrata	South East Pacific Coast	Actinopterygii
Gobiopsis exigua	South East Pacific Coast	Actinopterygii
Gobiopsis malekulae	South East Pacific Coast	Actinopterygii
Grahamichthys radiata	South East Pacific Coast	Actinopterygii
Grallenia lauensis	South East Pacific Coast	Actinopterygii
Grammatorcynus bilineatus	South East Pacific Coast	Actinopterygii
Gymnapogon urospilotus	South East Pacific Coast	Actinopterygii
Halicampus edmondsoni	South East Pacific Coast	Actinopterygii
Halicampus nitidus	South East Pacific Coast	Actinopterygii
Halieutaea nigra	South East Pacific Coast	Actinopterygii
Harpagifer bispinis	South East Pacific Coast	Actinopterygii
Helcogramma cerasina	South East Pacific Coast	Actinopterygii
Helcogramma fuscipectoris	South East Pacific Coast	Actinopterygii
Helcogramma gymnauchen	South East Pacific Coast	Actinopterygii
Helcogramma obtusirostris	South East Pacific Coast	Actinopterygii
Hippichthys spicifer	South East Pacific Coast	Actinopterygii
Hippocampus ingens	South East Pacific Coast	Actinopterygii
Hypopleuron caninum	South East Pacific Coast	Actinopterygii
Kamoharaia megastoma	South East Pacific Coast	Actinopterygii
Lepidopus fitchi	South East Pacific Coast	Actinopterygii
Leptonotus elevatus	South East Pacific Coast	Actinopterygii
Leptonotus norae	South East Pacific Coast	Actinopterygii
Lophogobius bleekeri	South East Pacific Coast	Actinopterygii
Lycenchelys maoriensis	South East Pacific Coast	Actinopterygii
Lycenchelys monstrosa	South East Pacific Coast	Actinopterygii
Lycodapus antarcticus	South East Pacific Coast	Actinopterygii
Lycodapus pachysoma	South East Pacific Coast	Actinopterygii
Lycodichthys antarcticus	South East Pacific Coast	Actinopterygii
Lycodichthys dearborni	South East Pacific Coast	Actinopterygii
Meiacanthus phaeus	South East Pacific Coast	Actinopterygii
Meuschenia scaber	South East Pacific Coast	Actinopterygii
Micrognathus natans	South East Pacific Coast	Actinopterygii
Minyichthys myersi	South East Pacific Coast	Actinopterygii
Mycteroperca xenarcha	South East Pacific Coast	Actinopterygii
Myxodagnus walkeri	South East Pacific Coast	Actinopterygii
Notoclinops segmentatus	South East Pacific Coast	Actinopterygii
Novaculops koteamea	South East Pacific Coast	Actinopterygii
Oligoplites altus	South East Pacific Coast	Actinopterygii
Oneirodes anisacanthus	South East Pacific Coast	Actinopterygii
Ostorhinchus neotes	South East Pacific Coast	Actinopterygii
Ostorhinchus sinus	South East Pacific Coast	Actinopterygii
Oxyurichthys cornutus	South East Pacific Coast	Actinopterygii
Oxyurichthys tentacularis	South East Pacific Coast	Actinopterygii

**Rare Species**	**System**	**Class**
Palutrus scapulopunctatus	South East Pacific Coast	Actinopterygii
Parabothus chlorospilus	South East Pacific Coast	Actinopterygii
Paracheilinus rubricaudalis	South East Pacific Coast	Actinopterygii
Paragobiodon melanosoma	South East Pacific Coast	Actinopterygii
Paragobiodon modestus	South East Pacific Coast	Actinopterygii
Paralichthys woolmani	South East Pacific Coast	Actinopterygii
Paraliparis nassarum	South East Pacific Coast	Actinopterygii
Paramonacanthus curtorhynchos	South East Pacific Coast	Actinopterygii
Parioglossus rainfordi	South East Pacific Coast	Actinopterygii
Phyllorhinichthys balushkini	South East Pacific Coast	Actinopterygii
Plectroglyphidodon sindonis	South East Pacific Coast	Actinopterygii
Poecilopsetta multiradiata	South East Pacific Coast	Actinopterygii
Pomacentrus bankanensis	South East Pacific Coast	Actinopterygii
Pomacentrus microspilus	South East Pacific Coast	Actinopterygii
Pomacentrus moluccensis	South East Pacific Coast	Actinopterygii
Pomacentrus nagasakiensis	South East Pacific Coast	Actinopterygii
Pomacentrus nigromarginatus	South East Pacific Coast	Actinopterygii
Pomacentrus taeniometopon	South East Pacific Coast	Actinopterygii
Porogadus atripectus	South East Pacific Coast	Actinopterygii
Priolepis limbatosquamis	South East Pacific Coast	Actinopterygii
Priolepis pallidicincta	South East Pacific Coast	Actinopterygii
Pseudamiops diaphanes	South East Pacific Coast	Actinopterygii
Pseudonus acutus	South East Pacific Coast	Actinopterygii
Psilogobius mainlandi	South East Pacific Coast	Actinopterygii
Pterygotrigla pauli	South East Pacific Coast	Actinopterygii
Redigobius balteatus	South East Pacific Coast	Actinopterygii
Saccopharynx schmidti	South East Pacific Coast	Actinopterygii
Scarus quoyi	South East Pacific Coast	Actinopterygii
Schindleria pietschmanni	South East Pacific Coast	Actinopterygii
Scomber australasicus	South East Pacific Coast	Actinopterygii
Siganus vermiculatus	South East Pacific Coast	Actinopterygii
Siokunichthys herrei	South East Pacific Coast	Actinopterygii
Siphamia majimai	South East Pacific Coast	Actinopterygii
Siphamia randalli	South East Pacific Coast	Actinopterygii
Siphamia spinicola	South East Pacific Coast	Actinopterygii
Solegnathus hardwickii	South East Pacific Coast	Actinopterygii
Solegnathus spinosissimus	South East Pacific Coast	Actinopterygii
Sphoeroides lispus	South East Pacific Coast	Actinopterygii
Spottobrotula amaculata	South East Pacific Coast	Actinopterygii
Stalix histrio	South East Pacific Coast	Actinopterygii
Stegastes gascoynei	South East Pacific Coast	Actinopterygii
Stonogobiops medon	South East Pacific Coast	Actinopterygii
Stonogobiops yasha	South East Pacific Coast	Actinopterygii
Strongylura exilis	South East Pacific Coast	Actinopterygii
Strophidon sathete	South East Pacific Coast	Actinopterygii

**Rare Species**	**System**	**Class**
Sueviota atrinasa	South East Pacific Coast	Actinopterygii
Sueviota lachneri	South East Pacific Coast	Actinopterygii
Suezichthys notatus	South East Pacific Coast	Actinopterygii
Syngnathus euchrous	South East Pacific Coast	Actinopterygii
Torquigener brevipinnis	South East Pacific Coast	Actinopterygii
Torquigener hicksi	South East Pacific Coast	Actinopterygii
Torquigener parcuspinus	South East Pacific Coast	Actinopterygii
Torquigener randalli	South East Pacific Coast	Actinopterygii
Trachyrhamphus bicoarctatus	South East Pacific Coast	Actinopterygii
Trachyscorpia osheri	South East Pacific Coast	Actinopterygii
Trimma anaima	South East Pacific Coast	Actinopterygii
Trimma cana	South East Pacific Coast	Actinopterygii
Trimma capostriatum	South East Pacific Coast	Actinopterygii
Trimma finistrinum	South East Pacific Coast	Actinopterygii
Trimma flavatrum	South East Pacific Coast	Actinopterygii
Trimma lantana	South East Pacific Coast	Actinopterygii
Trimma macrophthalmus	South East Pacific Coast	Actinopterygii
Trimma nasa	South East Pacific Coast	Actinopterygii
Trimma nomurai	South East Pacific Coast	Actinopterygii
Trimma okinawae	South East Pacific Coast	Actinopterygii
Trimma pajama	South East Pacific Coast	Actinopterygii
Trimma preclarum	South East Pacific Coast	Actinopterygii
Trimma striatum	South East Pacific Coast	Actinopterygii
Trimma xanthochrum	South East Pacific Coast	Actinopterygii
Trimmatom macropodus	South East Pacific Coast	Actinopterygii
Trimmatom zapotes	South East Pacific Coast	Actinopterygii
Tylerius spinosissimus	South East Pacific Coast	Actinopterygii
Yarica hyalosoma	South East Pacific Coast	Actinopterygii
Zoramia flebila	South East Pacific Coast	Actinopterygii
Asymbolus galacticus	South East Pacific Coast	Elasmobranchii
Bathyraja albomaculata	South East Pacific Coast	Elasmobranchii
Bathyraja tzinovskii	South East Pacific Coast	Elasmobranchii
Mobula eregoodootenkee	South East Pacific Coast	Elasmobranchii
Mobula kuhlii	South East Pacific Coast	Elasmobranchii
Sinobatis borneensis	South East Pacific Coast	Elasmobranchii
Stegostoma fasciatum	South East Pacific Coast	Elasmobranchii
Urolophus piperatus	South East Pacific Coast	Elasmobranchii
Acanthaluteres spilomelanurus	South West Pacific Coast	Actinopterygii
Acanthaluteres vittiger	South West Pacific Coast	Actinopterygii
Acromycter alcocki	South West Pacific Coast	Actinopterygii
Aetapcus maculatus	South West Pacific Coast	Actinopterygii
Aioliops tetrophthalmus	South West Pacific Coast	Actinopterygii
Alabes bathys	South West Pacific Coast	Actinopterygii
Alabes dorsalis	South West Pacific Coast	Actinopterygii
Alabes hoesei	South West Pacific Coast	Actinopterygii

**Rare Species**	**System**	**Class**
Alabes parvula	South West Pacific Coast	Actinopterygii
Alepocephalus antipodianus	South West Pacific Coast	Actinopterygii
Alepocephalus blanfordii	South West Pacific Coast	Actinopterygii
Alertichthys blacki	South West Pacific Coast	Actinopterygii
Allocyttus niger	South West Pacific Coast	Actinopterygii
Andamia amphibius	South West Pacific Coast	Actinopterygii
Anguilla australis	South West Pacific Coast	Actinopterygii
Anguilla dieffenbachii	South West Pacific Coast	Actinopterygii
Anguilla nebulosa	South West Pacific Coast	Actinopterygii
Anguilla reinhardtii	South West Pacific Coast	Actinopterygii
Antipodocottus megalops	South West Pacific Coast	Actinopterygii
Apogonichthyoides uninotatus	South West Pacific Coast	Actinopterygii
Apopterygion alta	South West Pacific Coast	Actinopterygii
Apopterygion oculus	South West Pacific Coast	Actinopterygii
Apterichtus australis	South West Pacific Coast	Actinopterygii
Argyrosomus japonicus	South West Pacific Coast	Actinopterygii
Arius microcephalus	South West Pacific Coast	Actinopterygii
Arius oetik	South West Pacific Coast	Actinopterygii
Arnoglossus boops	South West Pacific Coast	Actinopterygii
Arothron carduus	South West Pacific Coast	Actinopterygii
Aspasmogaster occidentalis	South West Pacific Coast	Actinopterygii
Assiculoides desmonotus	South West Pacific Coast	Actinopterygii
Asthenomacrurus victoris	South West Pacific Coast	Actinopterygii
Atractoscion aequidens	South West Pacific Coast	Actinopterygii
Aulastomatomorpha phospherops	South West Pacific Coast	Actinopterygii
Barathrodemus nasutus	South West Pacific Coast	Actinopterygii
Barathronus maculatus	South West Pacific Coast	Actinopterygii
Bascanichthys myersi	South West Pacific Coast	Actinopterygii
Bassozetus nasus	South West Pacific Coast	Actinopterygii
Bathophilus altipinnis	South West Pacific Coast	Actinopterygii
Bathyaploactis curtisensis	South West Pacific Coast	Actinopterygii
Bathycongrus aequoreus	South West Pacific Coast	Actinopterygii
Bathycongrus nasicus	South West Pacific Coast	Actinopterygii
Bathycongrus trilineatus	South West Pacific Coast	Actinopterygii
Bathydraco macrolepis	South West Pacific Coast	Actinopterygii
Bathygadus bowersi	South West Pacific Coast	Actinopterygii
Bathylagus tenuis	South West Pacific Coast	Actinopterygii
Bathymicrops brevianalis	South West Pacific Coast	Actinopterygii
Bathypterois longicauda	South West Pacific Coast	Actinopterygii
Bathypterois oddi	South West Pacific Coast	Actinopterygii
Bathypterois ventralis	South West Pacific Coast	Actinopterygii
Benthodesmus macrophthalmus	South West Pacific Coast	Actinopterygii
Benthodesmus neglectus	South West Pacific Coast	Actinopterygii
Benthodesmus papua	South West Pacific Coast	Actinopterygii
Bhanotia nuda	South West Pacific Coast	Actinopterygii

**Rare Species**	**System**	**Class**
Bhanotia pauciradiata	South West Pacific Coast	Actinopterygii
Bothrocara molle	South West Pacific Coast	Actinopterygii
Bovitrigla acanthomoplate	South West Pacific Coast	Actinopterygii
Brachionichthys australis	South West Pacific Coast	Actinopterygii
Brotulotaenia brevicauda	South West Pacific Coast	Actinopterygii
Brotulotaenia crassa	South West Pacific Coast	Actinopterygii
Bryaninops earlei	South West Pacific Coast	Actinopterygii
Canthigaster callisterna	South West Pacific Coast	Actinopterygii
Canthigaster punctatissima	South West Pacific Coast	Actinopterygii
Careproctus ampliceps	South West Pacific Coast	Actinopterygii
Careproctus improvisus	South West Pacific Coast	Actinopterygii
Careproctus paxtoni	South West Pacific Coast	Actinopterygii
Careproctus vladibeckeri	South West Pacific Coast	Actinopterygii
Caristius macropus	South West Pacific Coast	Actinopterygii
Cataetyx simus	South West Pacific Coast	Actinopterygii
Centroberyx gerrardi	South West Pacific Coast	Actinopterygii
Cepola haastii	South West Pacific Coast	Actinopterygii
Cetomimoides parri	South West Pacific Coast	Actinopterygii
Cetonurichthys subinflatus	South West Pacific Coast	Actinopterygii
Chaenodraco wilsoni	South West Pacific Coast	Actinopterygii
Chaenophryne melanorhabdus	South West Pacific Coast	Actinopterygii
Chascanopsetta prorigera	South West Pacific Coast	Actinopterygii
Chaunacops coloratus	South West Pacific Coast	Actinopterygii
Chaunacops spinosus	South West Pacific Coast	Actinopterygii
Chaunax nudiventer	South West Pacific Coast	Actinopterygii
Chaunax pictus	South West Pacific Coast	Actinopterygii
Chiasmodon subniger	South West Pacific Coast	Actinopterygii
Chionodraco hamatus	South West Pacific Coast	Actinopterygii
Chionodraco myersi	South West Pacific Coast	Actinopterygii
Chlorophthalmus mento	South West Pacific Coast	Actinopterygii
Choerodon melanostigma	South West Pacific Coast	Actinopterygii
Choeroichthys suillus	South West Pacific Coast	Actinopterygii
Chromis dispilus	South West Pacific Coast	Actinopterygii
Chrysiptera traceyi	South West Pacific Coast	Actinopterygii
Cirrhilabrus joanallenae	South West Pacific Coast	Actinopterygii
Coelorinchus cookianus	South West Pacific Coast	Actinopterygii
Coelorinchus gladius	South West Pacific Coast	Actinopterygii
Coelorinchus horribilis	South West Pacific Coast	Actinopterygii
Coelorinchus macrolepis	South West Pacific Coast	Actinopterygii
Coelorinchus notatus	South West Pacific Coast	Actinopterygii
Coelorinchus quadricristatus	South West Pacific Coast	Actinopterygii
Coelorinchus quincunciatus	South West Pacific Coast	Actinopterygii
Coelorinchus spilonotus	South West Pacific Coast	Actinopterygii
Coelorinchus weberi	South West Pacific Coast	Actinopterygii
Coloconger raniceps	South West Pacific Coast	Actinopterygii

**Rare Species**	**System**	**Class**
Congiopodus coriaceus	South West Pacific Coast	Actinopterygii
Conidens samoensis	South West Pacific Coast	Actinopterygii
Coryphaenoides camurus	South West Pacific Coast	Actinopterygii
Coryphaenoides dubius	South West Pacific Coast	Actinopterygii
Coryphaenoides ferrieri	South West Pacific Coast	Actinopterygii
Coryphaenoides lecointei	South West Pacific Coast	Actinopterygii
Coryphaenoides longicirrhus	South West Pacific Coast	Actinopterygii
Coryphaenoides orthogrammus	South West Pacific Coast	Actinopterygii
Coryphaenoides woodmasoni	South West Pacific Coast	Actinopterygii
Corythoichthys conspicillatus	South West Pacific Coast	Actinopterygii
Cottunculus nudus	South West Pacific Coast	Actinopterygii
Cubiceps kotlyari	South West Pacific Coast	Actinopterygii
Cyclothone signata	South West Pacific Coast	Actinopterygii
Cygnodraco mawsoni	South West Pacific Coast	Actinopterygii
Dactylagnus mundus	South West Pacific Coast	Actinopterygii
Diaphus drachmanni	South West Pacific Coast	Actinopterygii
Dibranchus nasutus	South West Pacific Coast	Actinopterygii
Dicrolene filamentosa	South West Pacific Coast	Actinopterygii
Dicrolene multifilis	South West Pacific Coast	Actinopterygii
Dicrolene nigricaudis	South West Pacific Coast	Actinopterygii
Dieidolycus leptodermatus	South West Pacific Coast	Actinopterygii
Diplophos pacificus	South West Pacific Coast	Actinopterygii
Dolloidraco longedorsalis	South West Pacific Coast	Actinopterygii
Dunckerocampus chapmani	South West Pacific Coast	Actinopterygii
Dysomma bucephalus	South West Pacific Coast	Actinopterygii
Ebinania australiae	South West Pacific Coast	Actinopterygii
Ebinania macquariensis	South West Pacific Coast	Actinopterygii
Ecsenius alleni	South West Pacific Coast	Actinopterygii
Ecsenius fijiensis	South West Pacific Coast	Actinopterygii
Ecsenius isos	South West Pacific Coast	Actinopterygii
Ecsenius pardus	South West Pacific Coast	Actinopterygii
Ecsenius stictus	South West Pacific Coast	Actinopterygii
Elassodiscus caudatus	South West Pacific Coast	Actinopterygii
Enigmacanthus filamentosus	South West Pacific Coast	Actinopterygii
Enneapterygius clarkae	South West Pacific Coast	Actinopterygii
Entomacrodus vermiculatus	South West Pacific Coast	Actinopterygii
Entomacrodus williamsi	South West Pacific Coast	Actinopterygii
Epinephelus daemelii	South West Pacific Coast	Actinopterygii
Eubalichthys bucephalus	South West Pacific Coast	Actinopterygii
Eubalichthys caeruleoguttatus	South West Pacific Coast	Actinopterygii
Eubalichthys gunnii	South West Pacific Coast	Actinopterygii
Eubalichthys mosaicus	South West Pacific Coast	Actinopterygii
Eurypleuron owasianum	South West Pacific Coast	Actinopterygii
Eusurculus andamanensis	South West Pacific Coast	Actinopterygii
Eviota brahmi	South West Pacific Coast	Actinopterygii

Rare Species	System	Class
Eviota dorsopurpurea	South West Pacific Coast	Actinopterygii
Eviota karaspila	South West Pacific Coast	Actinopterygii
Eviota kermadecensis	South West Pacific Coast	Actinopterygii
Eviota lateritea	South West Pacific Coast	Actinopterygii
Eviota mikiae	South West Pacific Coast	Actinopterygii
Eviota mimica	South West Pacific Coast	Actinopterygii
Eviota minuta	South West Pacific Coast	Actinopterygii
Eviota pinocchioi	South West Pacific Coast	Actinopterygii
Eviota richardi	South West Pacific Coast	Actinopterygii
Eviota singula	South West Pacific Coast	Actinopterygii
Eviota thamani	South West Pacific Coast	Actinopterygii
Festucalex cinctus	South West Pacific Coast	Actinopterygii
Festucalex rufus	South West Pacific Coast	Actinopterygii
Filicampus tigris	South West Pacific Coast	Actinopterygii
Foa yamba	South West Pacific Coast	Actinopterygii
Gavialiceps taeniola	South West Pacific Coast	Actinopterygii
Genypterus tigerinus	South West Pacific Coast	Actinopterygii
Gigantactis savagei	South West Pacific Coast	Actinopterygii
Girella cyanea	South West Pacific Coast	Actinopterygii
Girella elevata	South West Pacific Coast	Actinopterygii
Gnatholepis gymnocara	South West Pacific Coast	Actinopterygii
Gnathophis nasutus	South West Pacific Coast	Actinopterygii
Grallenia lauensis	South West Pacific Coast	Actinopterygii
Gulaphallus panayensis	South West Pacific Coast	Actinopterygii
Gymnapogon japonicus	South West Pacific Coast	Actinopterygii
Halosaurus attenuatus	South West Pacific Coast	Actinopterygii
Halosaurus carinicauda	South West Pacific Coast	Actinopterygii
Halosaurus radiatus	South West Pacific Coast	Actinopterygii
Harpadon squamosus	South West Pacific Coast	Actinopterygii
Helcogramma cerasina	South West Pacific Coast	Actinopterygii
Helicolenus barathri	South West Pacific Coast	Actinopterygii
Hemigobius mingi	South West Pacific Coast	Actinopterygii
Heraldia nocturna	South West Pacific Coast	Actinopterygii
Hetereleotris readerae	South West Pacific Coast	Actinopterygii
Heteroclinus johnstoni	South West Pacific Coast	Actinopterygii
Hippocampus breviceps	South West Pacific Coast	Actinopterygii
Hippocampus curvicuspis	South West Pacific Coast	Actinopterygii
Hippocampus fuscus	South West Pacific Coast	Actinopterygii
Hippocampus grandiceps	South West Pacific Coast	Actinopterygii
Hippocampus jugumus	South West Pacific Coast	Actinopterygii
Hippocampus minotaur	South West Pacific Coast	Actinopterygii
Hippocampus procerus	South West Pacific Coast	Actinopterygii
Hippocampus pusillus	South West Pacific Coast	Actinopterygii
Hippocampus whitei	South West Pacific Coast	Actinopterygii
Hippocampus zebra	South West Pacific Coast	Actinopterygii

Rare Species	System	Class
Holcomycteronus profundissimus	South West Pacific Coast	Actinopterygii
Holcomycteronus pterotus	South West Pacific Coast	Actinopterygii
Hoplostethus melanopeza	South West Pacific Coast	Actinopterygii
Hoplostethus mento	South West Pacific Coast	Actinopterygii
Hygophum atratum	South West Pacific Coast	Actinopterygii
Hyporthodus ergastularius	South West Pacific Coast	Actinopterygii
Ichthyococcus parini	South West Pacific Coast	Actinopterygii
Idiotropiscis lumnitzeri	South West Pacific Coast	Actinopterygii
Ilypnus gilberti	South West Pacific Coast	Actinopterygii
Iniistius griffithsi	South West Pacific Coast	Actinopterygii
Istigobius campbelli	South West Pacific Coast	Actinopterygii
Istigobius hoesei	South West Pacific Coast	Actinopterygii
Jaydia photogaster	South West Pacific Coast	Actinopterygii
Kaupus costatus	South West Pacific Coast	Actinopterygii
Kimblaeus bassensis	South West Pacific Coast	Actinopterygii
Kopua nuimata	South West Pacific Coast	Actinopterygii
Kuiterichthys furcipilis	South West Pacific Coast	Actinopterygii
Laemonema filodorsale	South West Pacific Coast	Actinopterygii
Lampanyctus hubbsi	South West Pacific Coast	Actinopterygii
Lampanyctus omostigma	South West Pacific Coast	Actinopterygii
Lamprogrammus shcherbachevi	South West Pacific Coast	Actinopterygii
Lasiognathus saccostoma	South West Pacific Coast	Actinopterygii
Lepidonotothen squamifrons	South West Pacific Coast	Actinopterygii
Lepidotrigla annamarae	South West Pacific Coast	Actinopterygii
Lepidotrigla musorstom	South West Pacific Coast	Actinopterygii
Leptobrotula breviventralis	South West Pacific Coast	Actinopterygii
Leptoderma affinis	South West Pacific Coast	Actinopterygii
Leptoichthys fistularius	South West Pacific Coast	Actinopterygii
Leptonotus elevatus	South West Pacific Coast	Actinopterygii
Leptonotus norae	South West Pacific Coast	Actinopterygii
Liparis fucensis	South West Pacific Coast	Actinopterygii
Lissocampus filum	South West Pacific Coast	Actinopterygii
Lissocampus runa	South West Pacific Coast	Actinopterygii
Lophiodes maculatus	South West Pacific Coast	Actinopterygii
Lophotus lacepede	South West Pacific Coast	Actinopterygii
Lumpenopsis hypochroma	South West Pacific Coast	Actinopterygii
Lycenchelys antarctica	South West Pacific Coast	Actinopterygii
Lycenchelys jordani	South West Pacific Coast	Actinopterygii
Lycenchelys maoriensis	South West Pacific Coast	Actinopterygii
Lycenchelys scaurus	South West Pacific Coast	Actinopterygii
Lycodapus antarcticus	South West Pacific Coast	Actinopterygii
Lycodapus endemoscotus	South West Pacific Coast	Actinopterygii
Lycodichthys antarcticus	South West Pacific Coast	Actinopterygii
Lycodichthys dearborni	South West Pacific Coast	Actinopterygii
Malacocephalus laevis	South West Pacific Coast	Actinopterygii

Rare Species	System	Class
Marilyna pleurosticta	South West Pacific Coast	Actinopterygii
Maroubra perserrata	South West Pacific Coast	Actinopterygii
Matanui profundum	South West Pacific Coast	Actinopterygii
Mecaenichthys immaculatus	South West Pacific Coast	Actinopterygii
Meiacanthus limbatus	South West Pacific Coast	Actinopterygii
Melamphaes microps	South West Pacific Coast	Actinopterygii
Mephisto fraserbrunneri	South West Pacific Coast	Actinopterygii
Meuschenia freycineti	South West Pacific Coast	Actinopterygii
Meuschenia hippocrepis	South West Pacific Coast	Actinopterygii
Meuschenia trachylepis	South West Pacific Coast	Actinopterygii
Microbrotula greenfieldi	South West Pacific Coast	Actinopterygii
Micromesistius australis	South West Pacific Coast	Actinopterygii
Microphotolepis schmidti	South West Pacific Coast	Actinopterygii
Microspathodon dorsalis	South West Pacific Coast	Actinopterygii
Mitotichthys semistriatus	South West Pacific Coast	Actinopterygii
Mitotichthys tuckeri	South West Pacific Coast	Actinopterygii
Monomitopus conjugator	South West Pacific Coast	Actinopterygii
Monomitopus nigripinnis	South West Pacific Coast	Actinopterygii
Muraenolepis evseenkoi	South West Pacific Coast	Actinopterygii
Nannobrachium indicum	South West Pacific Coast	Actinopterygii
Nansenia longicauda	South West Pacific Coast	Actinopterygii
Nelusetta ayraud	South West Pacific Coast	Actinopterygii
Nemapteryx caelata	South West Pacific Coast	Actinopterygii
Nemapteryx macronotacantha	South West Pacific Coast	Actinopterygii
Nematalosa nasus	South West Pacific Coast	Actinopterygii
Neoachiropsetta milfordi	South West Pacific Coast	Actinopterygii
Neobythites bimarginatus	South West Pacific Coast	Actinopterygii
Neobythites fijiensis	South West Pacific Coast	Actinopterygii
Neobythites neocaledoniensis	South West Pacific Coast	Actinopterygii
Neobythites stelliferoides	South West Pacific Coast	Actinopterygii
Neocaristius heemstrai	South West Pacific Coast	Actinopterygii
Neopagetopsis ionah	South West Pacific Coast	Actinopterygii
Neopataecus waterhousii	South West Pacific Coast	Actinopterygii
Neostethus villadolidi	South West Pacific Coast	Actinopterygii
Nesogobius greeni	South West Pacific Coast	Actinopterygii
Nesogobius maccullochi	South West Pacific Coast	Actinopterygii
Nezumia loricata	South West Pacific Coast	Actinopterygii
Nezumia merretti	South West Pacific Coast	Actinopterygii
Nezumia soela	South West Pacific Coast	Actinopterygii
Notiocampus ruber	South West Pacific Coast	Actinopterygii
Notolabrus cinctus	South West Pacific Coast	Actinopterygii
Notolepis annulata	South West Pacific Coast	Actinopterygii
Notolepis coatsi	South West Pacific Coast	Actinopterygii
Notothenia angustata	South West Pacific Coast	Actinopterygii
Notothenia coriiceps	South West Pacific Coast	Actinopterygii

**Rare Species**	**System**	**Class**
Notothenia neglecta	South West Pacific Coast	Actinopterygii
Notothenia rossii	South West Pacific Coast	Actinopterygii
Odontamblyopus rubicundus	South West Pacific Coast	Actinopterygii
Omegophora armilla	South West Pacific Coast	Actinopterygii
Omobranchus anolius	South West Pacific Coast	Actinopterygii
Oneirodes cristatus	South West Pacific Coast	Actinopterygii
Oneirodes dicromischus	South West Pacific Coast	Actinopterygii
Oneirodes micronema	South West Pacific Coast	Actinopterygii
Oneirodes schmidti	South West Pacific Coast	Actinopterygii
Ophichthus tetratrema	South West Pacific Coast	Actinopterygii
Ophthalmolepis lineolata	South West Pacific Coast	Actinopterygii
Ophthalmolycus amberensis	South West Pacific Coast	Actinopterygii
Ophthalmolycus bothriocephalus	South West Pacific Coast	Actinopterygii
Pachycara brachycephalum	South West Pacific Coast	Actinopterygii
Pachycara garricki	South West Pacific Coast	Actinopterygii
Pachycara suspectum	South West Pacific Coast	Actinopterygii
Parablennius yatabei	South West Pacific Coast	Actinopterygii
Paracheilinus rubricaudalis	South West Pacific Coast	Actinopterygii
Paraliparis albescens	South West Pacific Coast	Actinopterygii
Paraliparis antarcticus	South West Pacific Coast	Actinopterygii
Paraliparis cephalus	South West Pacific Coast	Actinopterygii
Paraliparis costatus	South West Pacific Coast	Actinopterygii
Paraliparis dactylosus	South West Pacific Coast	Actinopterygii
Paraliparis hawaiiensis	South West Pacific Coast	Actinopterygii
Paraliparis leobergi	South West Pacific Coast	Actinopterygii
Paraliparis meridionalis	South West Pacific Coast	Actinopterygii
Paramonacanthus lowei	South West Pacific Coast	Actinopterygii
Paramonacanthus otisensis	South West Pacific Coast	Actinopterygii
Paraploactis trachyderma	South West Pacific Coast	Actinopterygii
Parascolopsis melanophrys	South West Pacific Coast	Actinopterygii
Parkraemeria ornata	South West Pacific Coast	Actinopterygii
Parvilux boschmai	South West Pacific Coast	Actinopterygii
Pataecus fronto	South West Pacific Coast	Actinopterygii
Pegasus lancifer	South West Pacific Coast	Actinopterygii
Peristrominous dolosus	South West Pacific Coast	Actinopterygii
Petroscirtes lupus	South West Pacific Coast	Actinopterygii
Petroscirtes thepassii	South West Pacific Coast	Actinopterygii
Phallostethus lehi	South West Pacific Coast	Actinopterygii
Photostomias liemi	South West Pacific Coast	Actinopterygii
Phyllopteryx taeniolatus	South West Pacific Coast	Actinopterygii
Platytroctes mirus	South West Pacific Coast	Actinopterygii
Plicofollis dussumieri	South West Pacific Coast	Actinopterygii
Poecilopsetta multiradiata	South West Pacific Coast	Actinopterygii
Pogonophryne marmorata	South West Pacific Coast	Actinopterygii
Polyipnus danae	South West Pacific Coast	Actinopterygii

**Rare Species**	**System**	**Class**
Polyipnus ovatus	South West Pacific Coast	Actinopterygii
Polyipnus tridentifer	South West Pacific Coast	Actinopterygii
Pomacentrus albimaculus	South West Pacific Coast	Actinopterygii
Pomacentrus bintanensis	South West Pacific Coast	Actinopterygii
Pomacentrus callainus	South West Pacific Coast	Actinopterygii
Pomacentrus microspilus	South West Pacific Coast	Actinopterygii
Poromitra glochidiata	South West Pacific Coast	Actinopterygii
Praealticus tanegasimae	South West Pacific Coast	Actinopterygii
Praealticus triangulus	South West Pacific Coast	Actinopterygii
Priolepis triops	South West Pacific Coast	Actinopterygii
Psednos balushkini	South West Pacific Coast	Actinopterygii
Pseudocaranx wrighti	South West Pacific Coast	Actinopterygii
Pterygotrigla robertsi	South West Pacific Coast	Actinopterygii
Pugnaso curtirostris	South West Pacific Coast	Actinopterygii
Pycnocraspedum armatum	South West Pacific Coast	Actinopterygii
Pyramodon lindas	South West Pacific Coast	Actinopterygii
Redigobius isognathus	South West Pacific Coast	Actinopterygii
Redigobius macrostoma	South West Pacific Coast	Actinopterygii
Regalecus russelii	South West Pacific Coast	Actinopterygii
Rexea nakamurai	South West Pacific Coast	Actinopterygii
Saccopharynx schmidti	South West Pacific Coast	Actinopterygii
Sarda australis	South West Pacific Coast	Actinopterygii
Schindleria brevipinguis	South West Pacific Coast	Actinopterygii
Scolecenchelys castlei	South West Pacific Coast	Actinopterygii
Sebastiscus marmoratus	South West Pacific Coast	Actinopterygii
Sebastiscus tertius	South West Pacific Coast	Actinopterygii
Seriola hippos	South West Pacific Coast	Actinopterygii
Silhouettea insinuans	South West Pacific Coast	Actinopterygii
Silhouettea nuchipunctatus	South West Pacific Coast	Actinopterygii
Siphamia cephalotes	South West Pacific Coast	Actinopterygii
Siphamia guttulata	South West Pacific Coast	Actinopterygii
Solegnathus dunckeri	South West Pacific Coast	Actinopterygii
Solegnathus robustus	South West Pacific Coast	Actinopterygii
Solegnathus spinosissimus	South West Pacific Coast	Actinopterygii
Sphagemacrurus gibber	South West Pacific Coast	Actinopterygii
Sprattus muelleri	South West Pacific Coast	Actinopterygii
Stegastes apicalis	South West Pacific Coast	Actinopterygii
Stegastes insularis	South West Pacific Coast	Actinopterygii
Stigmatopora argus	South West Pacific Coast	Actinopterygii
Stigmatopora nigra	South West Pacific Coast	Actinopterygii
Stolephorus baganensis	South West Pacific Coast	Actinopterygii
Symphurus australis	South West Pacific Coast	Actinopterygii
Symphurus regani	South West Pacific Coast	Actinopterygii
Symphurus trifasciatus	South West Pacific Coast	Actinopterygii
Taenioides eruptionis	South West Pacific Coast	Actinopterygii

Rare Species	System	Class
Talismania bifurcata	South West Pacific Coast	Actinopterygii
Taranetzella lyoderma	South West Pacific Coast	Actinopterygii
Tasmanogobius lasti	South West Pacific Coast	Actinopterygii
Tetractenos glaber	South West Pacific Coast	Actinopterygii
Tetractenos hamiltoni	South West Pacific Coast	Actinopterygii
Thalasseleotris adela	South West Pacific Coast	Actinopterygii
Thamnaconus degeni	South West Pacific Coast	Actinopterygii
Thamnaconus fijiensis	South West Pacific Coast	Actinopterygii
Thorophos nexilis	South West Pacific Coast	Actinopterygii
Tomiyamichthys nudus	South West Pacific Coast	Actinopterygii
Torquigener altipinnis	South West Pacific Coast	Actinopterygii
Torquigener andersonae	South West Pacific Coast	Actinopterygii
Torquigener gloerfelti	South West Pacific Coast	Actinopterygii
Trachinotus coppingeri	South West Pacific Coast	Actinopterygii
Trachonurus yiwardaus	South West Pacific Coast	Actinopterygii
Trematomus bernacchii	South West Pacific Coast	Actinopterygii
Trematomus eulepidotus	South West Pacific Coast	Actinopterygii
Trematomus hansoni	South West Pacific Coast	Actinopterygii
Trematomus newnesi	South West Pacific Coast	Actinopterygii
Trematomus nicolai	South West Pacific Coast	Actinopterygii
Trematomus pennellii	South West Pacific Coast	Actinopterygii
Trematomus tokarevi	South West Pacific Coast	Actinopterygii
Triacanthodes intermedius	South West Pacific Coast	Actinopterygii
Trimma aturirii	South West Pacific Coast	Actinopterygii
Trimma fasciatum	South West Pacific Coast	Actinopterygii
Trimma finistrinum	South West Pacific Coast	Actinopterygii
Trimma habrum	South West Pacific Coast	Actinopterygii
Trimma hotsarihiensis	South West Pacific Coast	Actinopterygii
Trimma imaii	South West Pacific Coast	Actinopterygii
Trimma kardium	South West Pacific Coast	Actinopterygii
Trinorfolkia incisa	South West Pacific Coast	Actinopterygii
Tryssogobius flavolineatus	South West Pacific Coast	Actinopterygii
Vanacampus margaritifer	South West Pacific Coast	Actinopterygii
Vanacampus phillipi	South West Pacific Coast	Actinopterygii
Vanacampus poecilolaemus	South West Pacific Coast	Actinopterygii
Xenomystax atrarius	South West Pacific Coast	Actinopterygii
Zanclorhynchus spinifer	South West Pacific Coast	Actinopterygii
Zenopsis conchifer	South West Pacific Coast	Actinopterygii
Zenopsis stabilispinosa	South West Pacific Coast	Actinopterygii
Zoramia flebila	South West Pacific Coast	Actinopterygii
Zu elongatus	South West Pacific Coast	Actinopterygii
Apristurus platyrhynchus	South West Pacific Coast	Elasmobranchii
Bathyraja richardsoni	South West Pacific Coast	Elasmobranchii
Dipturus acrobelus	South West Pacific Coast	Elasmobranchii
Dipturus canutus	South West Pacific Coast	Elasmobranchii

**Rare Species**	**System**	**Class**
Dipturus gigas	South West Pacific Coast	Elasmobranchii
Dipturus melanospilus	South West Pacific Coast	Elasmobranchii
Galeus eastmani	South West Pacific Coast	Elasmobranchii
Galeus priapus	South West Pacific Coast	Elasmobranchii
Hemiscyllium freycineti	South West Pacific Coast	Elasmobranchii
Hemiscyllium michaeli	South West Pacific Coast	Elasmobranchii
Hemitrygon parvonigra	South West Pacific Coast	Elasmobranchii
Narcinops nelsoni	South West Pacific Coast	Elasmobranchii
Narke dipterygia	South West Pacific Coast	Elasmobranchii
Notoraja azurea	South West Pacific Coast	Elasmobranchii
Notoraja sapphira	South West Pacific Coast	Elasmobranchii
Okamejei leptoura	South West Pacific Coast	Elasmobranchii
Pastinachus solocirostris	South West Pacific Coast	Elasmobranchii
Pavoraja mosaica	South West Pacific Coast	Elasmobranchii
Pavoraja umbrosa	South West Pacific Coast	Elasmobranchii
Rajella challengeri	South West Pacific Coast	Elasmobranchii
Sinobatis bulbicauda	South West Pacific Coast	Elasmobranchii
Sinobatis filicauda	South West Pacific Coast	Elasmobranchii
Typhlonarke aysoni	South West Pacific Coast	Elasmobranchii
Urogymnus lobistoma	South West Pacific Coast	Elasmobranchii
Urolophus kapalensis	South West Pacific Coast	Elasmobranchii
Abalistes stellaris	Indian Ocean High Sea	Actinopterygii
Abudefduf sexfasciatus	Indian Ocean High Sea	Actinopterygii
Abudefduf vaigiensis	Indian Ocean High Sea	Actinopterygii
Acanthurus auranticavus	Indian Ocean High Sea	Actinopterygii
Acanthurus blochii	Indian Ocean High Sea	Actinopterygii
Acanthurus dussumieri	Indian Ocean High Sea	Actinopterygii
Acanthurus guttatus	Indian Ocean High Sea	Actinopterygii
Acanthurus leucosternon	Indian Ocean High Sea	Actinopterygii
Acanthurus lineatus	Indian Ocean High Sea	Actinopterygii
Acanthurus nigrofuscus	Indian Ocean High Sea	Actinopterygii
Acanthurus nigroris	Indian Ocean High Sea	Actinopterygii
Acanthurus pyroferus	Indian Ocean High Sea	Actinopterygii
Acanthurus tennentii	Indian Ocean High Sea	Actinopterygii
Acentrogobius audax	Indian Ocean High Sea	Actinopterygii
Acentrogobius caninus	Indian Ocean High Sea	Actinopterygii
Acentrogobius viridipunctatus	Indian Ocean High Sea	Actinopterygii
Acentronura tentaculata	Indian Ocean High Sea	Actinopterygii
Acreichthys hajam	Indian Ocean High Sea	Actinopterygii
Agonostomus telfairii	Indian Ocean High Sea	Actinopterygii
Alloblennius parvus	Indian Ocean High Sea	Actinopterygii
Aluterus monoceros	Indian Ocean High Sea	Actinopterygii
Aluterus scriptus	Indian Ocean High Sea	Actinopterygii
Ambassis interrupta	Indian Ocean High Sea	Actinopterygii
Amblyeleotris downingi	Indian Ocean High Sea	Actinopterygii

**Rare Species**	**System**	**Class**
Amblyglyphidodon batunai	Indian Ocean High Sea	Actinopterygii
Amblyglyphidodon indicus	Indian Ocean High Sea	Actinopterygii
Amblygobius albimaculatus	Indian Ocean High Sea	Actinopterygii
Amblygobius nocturnus	Indian Ocean High Sea	Actinopterygii
Amblygobius semicinctus	Indian Ocean High Sea	Actinopterygii
Amioides polyacanthus	Indian Ocean High Sea	Actinopterygii
Amphiprion akallopisos	Indian Ocean High Sea	Actinopterygii
Anguilla bengalensis	Indian Ocean High Sea	Actinopterygii
Anguilla bicolor	Indian Ocean High Sea	Actinopterygii
Anguilla marmorata	Indian Ocean High Sea	Actinopterygii
Anodontostoma chacunda	Indian Ocean High Sea	Actinopterygii
Antennablennius bifilum	Indian Ocean High Sea	Actinopterygii
Antennablennius variopunctatus	Indian Ocean High Sea	Actinopterygii
Antennatus linearis	Indian Ocean High Sea	Actinopterygii
Apogon campbelli	Indian Ocean High Sea	Actinopterygii
Apogon indicus	Indian Ocean High Sea	Actinopterygii
Apogon rubellus	Indian Ocean High Sea	Actinopterygii
Apogonichthyoides regani	Indian Ocean High Sea	Actinopterygii
Arcygobius baliurus	Indian Ocean High Sea	Actinopterygii
Arius sumatranus	Indian Ocean High Sea	Actinopterygii
Arothron immaculatus	Indian Ocean High Sea	Actinopterygii
Arothron mappa	Indian Ocean High Sea	Actinopterygii
Arothron multilineatus	Indian Ocean High Sea	Actinopterygii
Asterropteryx semipunctata	Indian Ocean High Sea	Actinopterygii
Astronesthes tanibe	Indian Ocean High Sea	Actinopterygii
Balistoides viridescens	Indian Ocean High Sea	Actinopterygii
Barathronus maculatus	Indian Ocean High Sea	Actinopterygii
Barbuligobius boehlkei	Indian Ocean High Sea	Actinopterygii
Bathygobius meggitti	Indian Ocean High Sea	Actinopterygii
Bathytyphlops sewelli	Indian Ocean High Sea	Actinopterygii
Benthodesmus oligoradiatus	Indian Ocean High Sea	Actinopterygii
Bryaninops erythrops	Indian Ocean High Sea	Actinopterygii
Bryaninops natans	Indian Ocean High Sea	Actinopterygii
Bryaninops ridens	Indian Ocean High Sea	Actinopterygii
Bryaninops tigris	Indian Ocean High Sea	Actinopterygii
Bryaninops yongei	Indian Ocean High Sea	Actinopterygii
Bryx analicarens	Indian Ocean High Sea	Actinopterygii
Bulbonaricus davaoensis	Indian Ocean High Sea	Actinopterygii
Cabillus lacertops	Indian Ocean High Sea	Actinopterygii
Cabillus tongarevae	Indian Ocean High Sea	Actinopterygii
Callogobius flavobrunneus	Indian Ocean High Sea	Actinopterygii
Calotomus spinidens	Indian Ocean High Sea	Actinopterygii
Canthigaster amboinensis	Indian Ocean High Sea	Actinopterygii
Canthigaster bennetti	Indian Ocean High Sea	Actinopterygii
Canthigaster cyanospilota	Indian Ocean High Sea	Actinopterygii

Rare Species	System	Class
Canthigaster natalensis	Indian Ocean High Sea	Actinopterygii
Canthigaster petersii	Indian Ocean High Sea	Actinopterygii
Canthigaster tyleri	Indian Ocean High Sea	Actinopterygii
Canthigaster valentini	Indian Ocean High Sea	Actinopterygii
Caranx lugubris	Indian Ocean High Sea	Actinopterygii
Cetoscarus ocellatus	Indian Ocean High Sea	Actinopterygii
Chaetodon octofasciatus	Indian Ocean High Sea	Actinopterygii
Chaunax flammeus	Indian Ocean High Sea	Actinopterygii
Cheilinus undulatus	Indian Ocean High Sea	Actinopterygii
Chelon planiceps	Indian Ocean High Sea	Actinopterygii
Chlorurus atrilunula	Indian Ocean High Sea	Actinopterygii
Chlorurus enneacanthus	Indian Ocean High Sea	Actinopterygii
Chlorurus gibbus	Indian Ocean High Sea	Actinopterygii
Chlorurus oedema	Indian Ocean High Sea	Actinopterygii
Chlorurus rhakoura	Indian Ocean High Sea	Actinopterygii
Choeroichthys brachysoma	Indian Ocean High Sea	Actinopterygii
Choeroichthys sculptus	Indian Ocean High Sea	Actinopterygii
Chromis axillaris	Indian Ocean High Sea	Actinopterygii
Chromis cinerascens	Indian Ocean High Sea	Actinopterygii
Chromis trialpha	Indian Ocean High Sea	Actinopterygii
Chrysiptera brownriggii	Indian Ocean High Sea	Actinopterygii
Cirripectes auritus	Indian Ocean High Sea	Actinopterygii
Cirripectes castaneus	Indian Ocean High Sea	Actinopterygii
Cirripectes filamentosus	Indian Ocean High Sea	Actinopterygii
Cirripectes gilberti	Indian Ocean High Sea	Actinopterygii
Cirripectes perustus	Indian Ocean High Sea	Actinopterygii
Cirripectes quagga	Indian Ocean High Sea	Actinopterygii
Cirripectes stigmaticus	Indian Ocean High Sea	Actinopterygii
Coelorinchus quadricristatus	Indian Ocean High Sea	Actinopterygii
Coris aygula	Indian Ocean High Sea	Actinopterygii
Coryogalops anomolus	Indian Ocean High Sea	Actinopterygii
Coryphaenoides woodmasoni	Indian Ocean High Sea	Actinopterygii
Corythoichthys amplexus	Indian Ocean High Sea	Actinopterygii
Corythoichthys flavofasciatus	Indian Ocean High Sea	Actinopterygii
Corythoichthys haematopterus	Indian Ocean High Sea	Actinopterygii
Corythoichthys nigripectus	Indian Ocean High Sea	Actinopterygii
Corythoichthys schultzi	Indian Ocean High Sea	Actinopterygii
Cosmocampus banneri	Indian Ocean High Sea	Actinopterygii
Cosmocampus maxweberi	Indian Ocean High Sea	Actinopterygii
Cosmocampus retropinnis	Indian Ocean High Sea	Actinopterygii
Cottunculus spinosus	Indian Ocean High Sea	Actinopterygii
Crenimugil buchanani	Indian Ocean High Sea	Actinopterygii
Ctenochaetus striatus	Indian Ocean High Sea	Actinopterygii
Ctenochaetus truncatus	Indian Ocean High Sea	Actinopterygii
Cubiceps kotlyari	Indian Ocean High Sea	Actinopterygii

Rare Species	System	Class
Dascyllus marginatus	Indian Ocean High Sea	Actinopterygii
Decapterus macarellus	Indian Ocean High Sea	Actinopterygii
Discordipinna griessingeri	Indian Ocean High Sea	Actinopterygii
Drombus key	Indian Ocean High Sea	Actinopterygii
Drombus triangularis	Indian Ocean High Sea	Actinopterygii
Dunckerocampus dactyliophorus	Indian Ocean High Sea	Actinopterygii
Ecsenius bicolor	Indian Ocean High Sea	Actinopterygii
Ecsenius lineatus	Indian Ocean High Sea	Actinopterygii
Ecsenius nalolo	Indian Ocean High Sea	Actinopterygii
Enchelynassa canina	Indian Ocean High Sea	Actinopterygii
Enneapterygius abeli	Indian Ocean High Sea	Actinopterygii
Enneapterygius clarkae	Indian Ocean High Sea	Actinopterygii
Enneapterygius gruschkai	Indian Ocean High Sea	Actinopterygii
Enneapterygius pusillus	Indian Ocean High Sea	Actinopterygii
Enneapterygius tutuilae	Indian Ocean High Sea	Actinopterygii
Epinephelus lanceolatus	Indian Ocean High Sea	Actinopterygii
Euthynnus affinis	Indian Ocean High Sea	Actinopterygii
Eviota bipunctata	Indian Ocean High Sea	Actinopterygii
Eviota distigma	Indian Ocean High Sea	Actinopterygii
Eviota guttata	Indian Ocean High Sea	Actinopterygii
Eviota indica	Indian Ocean High Sea	Actinopterygii
Eviota infulata	Indian Ocean High Sea	Actinopterygii
Eviota mikiae	Indian Ocean High Sea	Actinopterygii
Eviota nebulosa	Indian Ocean High Sea	Actinopterygii
Eviota notata	Indian Ocean High Sea	Actinopterygii
Eviota pardalota	Indian Ocean High Sea	Actinopterygii
Eviota prasina	Indian Ocean High Sea	Actinopterygii
Eviota sebreei	Indian Ocean High Sea	Actinopterygii
Eviota sigillata	Indian Ocean High Sea	Actinopterygii
Eviota springeri	Indian Ocean High Sea	Actinopterygii
Eviota zebrina	Indian Ocean High Sea	Actinopterygii
Exyrias belissimus	Indian Ocean High Sea	Actinopterygii
Feia nympha	Indian Ocean High Sea	Actinopterygii
Fusigobius humeralis	Indian Ocean High Sea	Actinopterygii
Fusigobius inframaculatus	Indian Ocean High Sea	Actinopterygii
Fusigobius maximus	Indian Ocean High Sea	Actinopterygii
Fusigobius neophytus	Indian Ocean High Sea	Actinopterygii
Fusigobius pallidus	Indian Ocean High Sea	Actinopterygii
Gnatholepis cauerensis	Indian Ocean High Sea	Actinopterygii
Gobiodon fulvus	Indian Ocean High Sea	Actinopterygii
Gobiodon prolixus	Indian Ocean High Sea	Actinopterygii
Gobiodon rivulatus	Indian Ocean High Sea	Actinopterygii
Gobiodon unicolor	Indian Ocean High Sea	Actinopterygii
Gobionotothen acuta	Indian Ocean High Sea	Actinopterygii
Gobius bontii	Indian Ocean High Sea	Actinopterygii

**Rare Species**	**System**	**Class**
Gymnapogon africanus	Indian Ocean High Sea	Actinopterygii
Gymnosarda unicolor	Indian Ocean High Sea	Actinopterygii
Gymnothorax favagineus	Indian Ocean High Sea	Actinopterygii
Gymnothorax flavimarginatus	Indian Ocean High Sea	Actinopterygii
Gymnothorax javanicus	Indian Ocean High Sea	Actinopterygii
Gymnothorax johnsoni	Indian Ocean High Sea	Actinopterygii
Gymnothorax polyuranodon	Indian Ocean High Sea	Actinopterygii
Halicampus macrorhynchus	Indian Ocean High Sea	Actinopterygii
Halicampus spinirostris	Indian Ocean High Sea	Actinopterygii
Halichoeres leptotaenia	Indian Ocean High Sea	Actinopterygii
Halieutopsis ingerorum	Indian Ocean High Sea	Actinopterygii
Harpadon translucens	Indian Ocean High Sea	Actinopterygii
Helcogramma alkamr	Indian Ocean High Sea	Actinopterygii
Helcogramma chica	Indian Ocean High Sea	Actinopterygii
Helcogramma ellioti	Indian Ocean High Sea	Actinopterygii
Helcogramma obtusirostris	Indian Ocean High Sea	Actinopterygii
Hetereleotris apora	Indian Ocean High Sea	Actinopterygii
Hetereleotris nebulofasciata	Indian Ocean High Sea	Actinopterygii
Hetereleotris vulgaris	Indian Ocean High Sea	Actinopterygii
Hippichthys cyanospilos	Indian Ocean High Sea	Actinopterygii
Hippichthys penicillus	Indian Ocean High Sea	Actinopterygii
Hippocampus fuscus	Indian Ocean High Sea	Actinopterygii
Hippocampus histrix	Indian Ocean High Sea	Actinopterygii
Hippocampus kelloggi	Indian Ocean High Sea	Actinopterygii
Hippocampus kuda	Indian Ocean High Sea	Actinopterygii
Hipposcarus harid	Indian Ocean High Sea	Actinopterygii
Hirculops cornifer	Indian Ocean High Sea	Actinopterygii
Holcomycteronus profundissimus	Indian Ocean High Sea	Actinopterygii
Iniistius griffithsi	Indian Ocean High Sea	Actinopterygii
Jaydia hungi	Indian Ocean High Sea	Actinopterygii
Jaydia queketti	Indian Ocean High Sea	Actinopterygii
Koumansetta hectori	Indian Ocean High Sea	Actinopterygii
Kyonemichthys rumengani	Indian Ocean High Sea	Actinopterygii
Lachneratus phasmaticus	Indian Ocean High Sea	Actinopterygii
Lagocephalus guentheri	Indian Ocean High Sea	Actinopterygii
Lagocephalus lunaris	Indian Ocean High Sea	Actinopterygii
Lagocephalus sceleratus	Indian Ocean High Sea	Actinopterygii
Lagocephalus spadiceus	Indian Ocean High Sea	Actinopterygii
Lates calcarifer	Indian Ocean High Sea	Actinopterygii
Leptocephalus giganteus	Indian Ocean High Sea	Actinopterygii
Leptoscarus vaigiensis	Indian Ocean High Sea	Actinopterygii
Lichia amia	Indian Ocean High Sea	Actinopterygii
Limnichthys nitidus	Indian Ocean High Sea	Actinopterygii
Lindbergichthys mizops	Indian Ocean High Sea	Actinopterygii
Lobulogobius omanensis	Indian Ocean High Sea	Actinopterygii

Rare Species	System	Class
Luposicya lupus	Indian Ocean High Sea	Actinopterygii
Macrodontogobius wilburi	Indian Ocean High Sea	Actinopterygii
Majungaichthys agalegae	Indian Ocean High Sea	Actinopterygii
Mascarenichthys heemstrai	Indian Ocean High Sea	Actinopterygii
Megalops cyprinoides	Indian Ocean High Sea	Actinopterygii
Meiacanthus fraseri	Indian Ocean High Sea	Actinopterygii
Mephisto fraserbrunneri	Indian Ocean High Sea	Actinopterygii
Micrognathus andersonii	Indian Ocean High Sea	Actinopterygii
Microphis argulus	Indian Ocean High Sea	Actinopterygii
Microphis millepunctatus	Indian Ocean High Sea	Actinopterygii
Mimoblennius atrocinctus	Indian Ocean High Sea	Actinopterygii
Minyichthys myersi	Indian Ocean High Sea	Actinopterygii
Nannocampus pictus	Indian Ocean High Sea	Actinopterygii
Naso annulatus	Indian Ocean High Sea	Actinopterygii
Naso elegans	Indian Ocean High Sea	Actinopterygii
Naso fageni	Indian Ocean High Sea	Actinopterygii
Naso tonganus	Indian Ocean High Sea	Actinopterygii
Netuma bilineata	Indian Ocean High Sea	Actinopterygii
Netuma thalassina	Indian Ocean High Sea	Actinopterygii
Oligolepis acutipennis	Indian Ocean High Sea	Actinopterygii
Omobranchus fasciolatus	Indian Ocean High Sea	Actinopterygii
Omobranchus punctatus	Indian Ocean High Sea	Actinopterygii
Opistognathus nigromarginatus	Indian Ocean High Sea	Actinopterygii
Osteomugil robustus	Indian Ocean High Sea	Actinopterygii
Ostichthys archiepiscopus	Indian Ocean High Sea	Actinopterygii
Ostorhinchus gularis	Indian Ocean High Sea	Actinopterygii
Oxyurichthys microlepis	Indian Ocean High Sea	Actinopterygii
Oxyurichthys ophthalmonema	Indian Ocean High Sea	Actinopterygii
Oxyurichthys tentacularis	Indian Ocean High Sea	Actinopterygii
Parablennius opercularis	Indian Ocean High Sea	Actinopterygii
Parablennius thysanius	Indian Ocean High Sea	Actinopterygii
Parachaeturichthys polynema	Indian Ocean High Sea	Actinopterygii
Paragobiodon echinocephalus	Indian Ocean High Sea	Actinopterygii
Paragobiodon lacunicolus	Indian Ocean High Sea	Actinopterygii
Paragobiodon melanosoma	Indian Ocean High Sea	Actinopterygii
Paragobiodon modestus	Indian Ocean High Sea	Actinopterygii
Paragobiodon xanthosoma	Indian Ocean High Sea	Actinopterygii
Paraliparis costatus	Indian Ocean High Sea	Actinopterygii
Paraliparis gracilis	Indian Ocean High Sea	Actinopterygii
Paraluteres prionurus	Indian Ocean High Sea	Actinopterygii
Paramonacanthus frenatus	Indian Ocean High Sea	Actinopterygii
Paramonacanthus pusillus	Indian Ocean High Sea	Actinopterygii
Paratrypauchen microcephalus	Indian Ocean High Sea	Actinopterygii
Parioglossus taeniatus	Indian Ocean High Sea	Actinopterygii
Penopus microphthalmus	Indian Ocean High Sea	Actinopterygii

**Rare Species**	**System**	**Class**
Pervagor aspricaudus	Indian Ocean High Sea	Actinopterygii
Pervagor janthinosoma	Indian Ocean High Sea	Actinopterygii
Pervagor melanocephalus	Indian Ocean High Sea	Actinopterygii
Petroscirtes mitratus	Indian Ocean High Sea	Actinopterygii
Phoxocampus belcheri	Indian Ocean High Sea	Actinopterygii
Phoxocampus diacanthus	Indian Ocean High Sea	Actinopterygii
Planiliza alata	Indian Ocean High Sea	Actinopterygii
Planiliza macrolepis	Indian Ocean High Sea	Actinopterygii
Plectroglyphidodon imparipennis	Indian Ocean High Sea	Actinopterygii
Plectroglyphidodon lacrymatus	Indian Ocean High Sea	Actinopterygii
Plectroglyphidodon leucozonus	Indian Ocean High Sea	Actinopterygii
Plectroglyphidodon phoenixensis	Indian Ocean High Sea	Actinopterygii
Plesiops mystaxus	Indian Ocean High Sea	Actinopterygii
Pleurosicya annandalei	Indian Ocean High Sea	Actinopterygii
Pleurosicya coerulea	Indian Ocean High Sea	Actinopterygii
Pleurosicya elongata	Indian Ocean High Sea	Actinopterygii
Pleurosicya fringilla	Indian Ocean High Sea	Actinopterygii
Pleurosicya mossambica	Indian Ocean High Sea	Actinopterygii
Pleurosicya muscarum	Indian Ocean High Sea	Actinopterygii
Pleurosicya occidentalis	Indian Ocean High Sea	Actinopterygii
Pleurosicya plicata	Indian Ocean High Sea	Actinopterygii
Pleurosicya prognatha	Indian Ocean High Sea	Actinopterygii
Plicofollis dussumieri	Indian Ocean High Sea	Actinopterygii
Plicofollis layardi	Indian Ocean High Sea	Actinopterygii
Plicofollis tonggol	Indian Ocean High Sea	Actinopterygii
Plotosus canius	Indian Ocean High Sea	Actinopterygii
Pomacanthus imperator	Indian Ocean High Sea	Actinopterygii
Pomacentrus albicaudatus	Indian Ocean High Sea	Actinopterygii
Pomacentrus aquilus	Indian Ocean High Sea	Actinopterygii
Pomacentrus baenschi	Indian Ocean High Sea	Actinopterygii
Pomacentrus nagasakiensis	Indian Ocean High Sea	Actinopterygii
Porogadus melanocephalus	Indian Ocean High Sea	Actinopterygii
Priolepis compita	Indian Ocean High Sea	Actinopterygii
Priolepis inhaca	Indian Ocean High Sea	Actinopterygii
Priolepis nocturna	Indian Ocean High Sea	Actinopterygii
Priolepis semidoliata	Indian Ocean High Sea	Actinopterygii
Psettodes erumei	Indian Ocean High Sea	Actinopterygii
Pseudamiops pellucidus	Indian Ocean High Sea	Actinopterygii
Pseudochromis caudalis	Indian Ocean High Sea	Actinopterygii
Pseudogobius melanostictus	Indian Ocean High Sea	Actinopterygii
Pseudojuloides severnsi	Indian Ocean High Sea	Actinopterygii
Pseudorhombus arsius	Indian Ocean High Sea	Actinopterygii
Pteragogus taeniops	Indian Ocean High Sea	Actinopterygii
Rastrelliger kanagurta	Indian Ocean High Sea	Actinopterygii
Rhinomuraena quaesita	Indian Ocean High Sea	Actinopterygii

**Rare Species**	**System**	**Class**
Salarias alboguttatus	Indian Ocean High Sea	Actinopterygii
Samariscus maculatus	Indian Ocean High Sea	Actinopterygii
Sarda orientalis	Indian Ocean High Sea	Actinopterygii
Sardinella fimbriata	Indian Ocean High Sea	Actinopterygii
Sardinella gibbosa	Indian Ocean High Sea	Actinopterygii
Saurenchelys lateromaculatus	Indian Ocean High Sea	Actinopterygii
Scarus caudofasciatus	Indian Ocean High Sea	Actinopterygii
Scarus falcipinnis	Indian Ocean High Sea	Actinopterygii
Scarus ferrugineus	Indian Ocean High Sea	Actinopterygii
Scarus festivus	Indian Ocean High Sea	Actinopterygii
Scarus frenatus	Indian Ocean High Sea	Actinopterygii
Scarus globiceps	Indian Ocean High Sea	Actinopterygii
Scarus niger	Indian Ocean High Sea	Actinopterygii
Scarus oviceps	Indian Ocean High Sea	Actinopterygii
Scarus prasiognathos	Indian Ocean High Sea	Actinopterygii
Scarus psittacus	Indian Ocean High Sea	Actinopterygii
Scarus quoyi	Indian Ocean High Sea	Actinopterygii
Scarus rubroviolaceus	Indian Ocean High Sea	Actinopterygii
Scarus russelii	Indian Ocean High Sea	Actinopterygii
Scarus scaber	Indian Ocean High Sea	Actinopterygii
Scarus tricolor	Indian Ocean High Sea	Actinopterygii
Scarus viridifucatus	Indian Ocean High Sea	Actinopterygii
Scomberomorus commerson	Indian Ocean High Sea	Actinopterygii
Silhouettea insinuans	Indian Ocean High Sea	Actinopterygii
Siokunichthys herrei	Indian Ocean High Sea	Actinopterygii
Siphamia mossambica	Indian Ocean High Sea	Actinopterygii
Solegnathus hardwickii	Indian Ocean High Sea	Actinopterygii
Sphoeroides pachygaster	Indian Ocean High Sea	Actinopterygii
Sphyraena barracuda	Indian Ocean High Sea	Actinopterygii
Spratelloides gracilis	Indian Ocean High Sea	Actinopterygii
Stalix histrio	Indian Ocean High Sea	Actinopterygii
Stanulus seychellensis	Indian Ocean High Sea	Actinopterygii
Stegastes fasciolatus	Indian Ocean High Sea	Actinopterygii
Stegastes nigricans	Indian Ocean High Sea	Actinopterygii
Stegastes obreptus	Indian Ocean High Sea	Actinopterygii
Stonogobiops nematodes	Indian Ocean High Sea	Actinopterygii
Strongylura strongylura	Indian Ocean High Sea	Actinopterygii
Strophidon sathete	Indian Ocean High Sea	Actinopterygii
Sueviota aprica	Indian Ocean High Sea	Actinopterygii
Sueviota lachneri	Indian Ocean High Sea	Actinopterygii
Takifugu oblongus	Indian Ocean High Sea	Actinopterygii
Taractichthys longipinnis	Indian Ocean High Sea	Actinopterygii
Terapon jarbua	Indian Ocean High Sea	Actinopterygii
Terapon puta	Indian Ocean High Sea	Actinopterygii
Terelabrus rubrovittatus	Indian Ocean High Sea	Actinopterygii

Rare Species	System	Class
Thamnaconus fajardoi	Indian Ocean High Sea	Actinopterygii
Thunnus tonggol	Indian Ocean High Sea	Actinopterygii
Thyrsitoides marleyi	Indian Ocean High Sea	Actinopterygii
Tomiyamichthys fourmanoiri	Indian Ocean High Sea	Actinopterygii
Torquigener hypselogeneion	Indian Ocean High Sea	Actinopterygii
Trachyrhamphus bicoarctatus	Indian Ocean High Sea	Actinopterygii
Trachyrhamphus longirostris	Indian Ocean High Sea	Actinopterygii
Triacanthus biaculeatus	Indian Ocean High Sea	Actinopterygii
Trimma anaima	Indian Ocean High Sea	Actinopterygii
Trimma annosum	Indian Ocean High Sea	Actinopterygii
Trimma dalerocheila	Indian Ocean High Sea	Actinopterygii
Trimma emeryi	Indian Ocean High Sea	Actinopterygii
Trimma flammeum	Indian Ocean High Sea	Actinopterygii
Trimma griffithsi	Indian Ocean High Sea	Actinopterygii
Trimma haima	Indian Ocean High Sea	Actinopterygii
Trimma hoesei	Indian Ocean High Sea	Actinopterygii
Trimma macrophthalmus	Indian Ocean High Sea	Actinopterygii
Trimma mendelssohni	Indian Ocean High Sea	Actinopterygii
Trimma sheppardi	Indian Ocean High Sea	Actinopterygii
Trimma striatum	Indian Ocean High Sea	Actinopterygii
Trimma volcana	Indian Ocean High Sea	Actinopterygii
Trimma winterbottomi	Indian Ocean High Sea	Actinopterygii
Trimmatom pharus	Indian Ocean High Sea	Actinopterygii
Trypauchen vagina	Indian Ocean High Sea	Actinopterygii
Valamugil speigleri	Indian Ocean High Sea	Actinopterygii
Valenciennea strigata	Indian Ocean High Sea	Actinopterygii
Xenisthmus polyzonatus	Indian Ocean High Sea	Actinopterygii
Xyelacyba myersi	Indian Ocean High Sea	Actinopterygii
Yongeichthys criniger	Indian Ocean High Sea	Actinopterygii
Zebrasoma desjardinii	Indian Ocean High Sea	Actinopterygii
Zebrasoma scopas	Indian Ocean High Sea	Actinopterygii
Zebrasoma velifer	Indian Ocean High Sea	Actinopterygii
Zebrasoma xanthurum	Indian Ocean High Sea	Actinopterygii
Aetomylaeus milvus	Indian Ocean High Sea	Elasmobranchii
Asymbolus parvus	Indian Ocean High Sea	Elasmobranchii
Bathyraja hesperafricana	Indian Ocean High Sea	Elasmobranchii
Brevitrygon imbricata	Indian Ocean High Sea	Elasmobranchii
Bythaelurus alcockii	Indian Ocean High Sea	Elasmobranchii
Centrophorus westraliensis	Indian Ocean High Sea	Elasmobranchii
Centrophorus zeehaani	Indian Ocean High Sea	Elasmobranchii
Chiloscyllium plagiosum	Indian Ocean High Sea	Elasmobranchii
Glyphis garricki	Indian Ocean High Sea	Elasmobranchii
Himantura leoparda	Indian Ocean High Sea	Elasmobranchii
Maculabatis ambigua	Indian Ocean High Sea	Elasmobranchii
Mobula birostris	Indian Ocean High Sea	Elasmobranchii

**Rare Species**	**System**	**Class**
Narcine brunnea	Indian Ocean High Sea	Elasmobranchii
Okamejei heemstrai	Indian Ocean High Sea	Elasmobranchii
Pristis microdon	Indian Ocean High Sea	Elasmobranchii
Rhizoprionodon taylori	Indian Ocean High Sea	Elasmobranchii
Scymnodon ichiharai	Indian Ocean High Sea	Elasmobranchii
Stegostoma fasciatum	Indian Ocean High Sea	Elasmobranchii
Acantholabrus palloni	North Atlantic High Sea	Actinopterygii
Acipenser oxyrinchus	North Atlantic High Sea	Actinopterygii
Alosa fallax	North Atlantic High Sea	Actinopterygii
Anarhichas denticulatus	North Atlantic High Sea	Actinopterygii
Anarhichas lupus	North Atlantic High Sea	Actinopterygii
Anarhichas minor	North Atlantic High Sea	Actinopterygii
Anguilla rostrata	North Atlantic High Sea	Actinopterygii
Aphanopus intermedius	North Atlantic High Sea	Actinopterygii
Argyripnus atlanticus	North Atlantic High Sea	Actinopterygii
Artediellus atlanticus	North Atlantic High Sea	Actinopterygii
Artediellus uncinatus	North Atlantic High Sea	Actinopterygii
Barathronus bicolor	North Atlantic High Sea	Actinopterygii
Bathyuroconger vicinus	North Atlantic High Sea	Actinopterygii
Benthodesmus simonyi	North Atlantic High Sea	Actinopterygii
Beryx decadactylus	North Atlantic High Sea	Actinopterygii
Callanthias ruber	North Atlantic High Sea	Actinopterygii
Capros aper	North Atlantic High Sea	Actinopterygii
Careproctus reinhardti	North Atlantic High Sea	Actinopterygii
Chelidonichthys cuculus	North Atlantic High Sea	Actinopterygii
Citharichthys arctifrons	North Atlantic High Sea	Actinopterygii
Citharichthys dinoceros	North Atlantic High Sea	Actinopterygii
Clupea harengus	North Atlantic High Sea	Actinopterygii
Conger conger	North Atlantic High Sea	Actinopterygii
Conger oceanicus	North Atlantic High Sea	Actinopterygii
Cosmocampus retropinnis	North Atlantic High Sea	Actinopterygii
Cottunculus microps	North Atlantic High Sea	Actinopterygii
Cottunculus spinosus	North Atlantic High Sea	Actinopterygii
Cubiceps capensis	North Atlantic High Sea	Actinopterygii
Cyclopterus lumpus	North Atlantic High Sea	Actinopterygii
Diaphus minax	North Atlantic High Sea	Actinopterygii
Dibranchus atlanticus	North Atlantic High Sea	Actinopterygii
Dibranchus tremendus	North Atlantic High Sea	Actinopterygii
Diplacanthopoma brachysoma	North Atlantic High Sea	Actinopterygii
Diplospinus multistriatus	North Atlantic High Sea	Actinopterygii
Dysommina proboscideus	North Atlantic High Sea	Actinopterygii
Echiodon dawsoni	North Atlantic High Sea	Actinopterygii
Echiodon dentatus	North Atlantic High Sea	Actinopterygii
Echiodon drummondii	North Atlantic High Sea	Actinopterygii
Enchelyopus cimbrius	North Atlantic High Sea	Actinopterygii

**Rare Species**	**System**	**Class**
Engraulis eurystole	North Atlantic High Sea	Actinopterygii
Epigonus telescopus	North Atlantic High Sea	Actinopterygii
Eumicrotremus spinosus	North Atlantic High Sea	Actinopterygii
Fodiator acutus	North Atlantic High Sea	Actinopterygii
Gadiculus argenteus	North Atlantic High Sea	Actinopterygii
Gadiculus thori	North Atlantic High Sea	Actinopterygii
Gadus morhua	North Atlantic High Sea	Actinopterygii
Gasterosteus aculeatus	North Atlantic High Sea	Actinopterygii
Glyptocephalus cynoglossus	North Atlantic High Sea	Actinopterygii
Gordiichthys leibyi	North Atlantic High Sea	Actinopterygii
Gymnelus viridis	North Atlantic High Sea	Actinopterygii
Helicolenus dactylopterus	North Atlantic High Sea	Actinopterygii
Holcomycteronus profundissimus	North Atlantic High Sea	Actinopterygii
Lepidorhombus whiffiagonis	North Atlantic High Sea	Actinopterygii
Leptoclinus maculatus	North Atlantic High Sea	Actinopterygii
Limanda ferruginea	North Atlantic High Sea	Actinopterygii
Liparis coheni	North Atlantic High Sea	Actinopterygii
Liparis inquilinus	North Atlantic High Sea	Actinopterygii
Lophius americanus	North Atlantic High Sea	Actinopterygii
Lophius budegassa	North Atlantic High Sea	Actinopterygii
Lophius piscatorius	North Atlantic High Sea	Actinopterygii
Lumpenus lampretaeformis	North Atlantic High Sea	Actinopterygii
Lycenchelys kolthoffi	North Atlantic High Sea	Actinopterygii
Lycenchelys paxillus	North Atlantic High Sea	Actinopterygii
Lycenchelys sarsii	North Atlantic High Sea	Actinopterygii
Lycenchelys verrillii	North Atlantic High Sea	Actinopterygii
Lycodes esmarkii	North Atlantic High Sea	Actinopterygii
Lycodes eudipleurostictus	North Atlantic High Sea	Actinopterygii
Lycodes frigidus	North Atlantic High Sea	Actinopterygii
Lycodes gracilis	North Atlantic High Sea	Actinopterygii
Lycodes lavalaei	North Atlantic High Sea	Actinopterygii
Lycodes pallidus	North Atlantic High Sea	Actinopterygii
Lycodes reticulatus	North Atlantic High Sea	Actinopterygii
Lycodes rossi	North Atlantic High Sea	Actinopterygii
Lycodonus mirabilis	North Atlantic High Sea	Actinopterygii
Macrourus berglax	North Atlantic High Sea	Actinopterygii
Melanogrammus aeglefinus	North Atlantic High Sea	Actinopterygii
Merluccius bilinearis	North Atlantic High Sea	Actinopterygii
Merluccius merluccius	North Atlantic High Sea	Actinopterygii
Molva dypterygia	North Atlantic High Sea	Actinopterygii
Molva molva	North Atlantic High Sea	Actinopterygii
Morone saxatilis	North Atlantic High Sea	Actinopterygii
Myoxocephalus scorpius	North Atlantic High Sea	Actinopterygii
Osmerus mordax	North Atlantic High Sea	Actinopterygii
Pachycara bulbiceps	North Atlantic High Sea	Actinopterygii

**Rare Species**	**System**	**Class**
Pachycara caribbaeum	North Atlantic High Sea	Actinopterygii
Pachycara crassiceps	North Atlantic High Sea	Actinopterygii
Pachycara thermophilum	North Atlantic High Sea	Actinopterygii
Pagellus bogaraveo	North Atlantic High Sea	Actinopterygii
Paracaristius nemorosus	North Atlantic High Sea	Actinopterygii
Paraliparis hystrix	North Atlantic High Sea	Actinopterygii
Paraliparis liparinus	North Atlantic High Sea	Actinopterygii
Peristedion longispatha	North Atlantic High Sea	Actinopterygii
Pholis gunnellus	North Atlantic High Sea	Actinopterygii
Phycis blennoides	North Atlantic High Sea	Actinopterygii
Pollachius virens	North Atlantic High Sea	Actinopterygii
Polyipnus clarus	North Atlantic High Sea	Actinopterygii
Polyipnus polli	North Atlantic High Sea	Actinopterygii
Polyprion americanus	North Atlantic High Sea	Actinopterygii
Pterycombus petersii	North Atlantic High Sea	Actinopterygii
Pungitius pungitius	North Atlantic High Sea	Actinopterygii
Reinhardtius hippoglossoides	North Atlantic High Sea	Actinopterygii
Salmo salar	North Atlantic High Sea	Actinopterygii
Scomber scombrus	North Atlantic High Sea	Actinopterygii
Sebastes fasciatus	North Atlantic High Sea	Actinopterygii
Syngnathus fuscus	North Atlantic High Sea	Actinopterygii
Tautogolabrus adspersus	North Atlantic High Sea	Actinopterygii
Trachurus picturatus	North Atlantic High Sea	Actinopterygii
Trachurus trachurus	North Atlantic High Sea	Actinopterygii
Triglops murrayi	North Atlantic High Sea	Actinopterygii
Triglops pingelii	North Atlantic High Sea	Actinopterygii
Urophycis tenuis	North Atlantic High Sea	Actinopterygii
Zenopsis conchifer	North Atlantic High Sea	Actinopterygii
Zoarces americanus	North Atlantic High Sea	Actinopterygii
Dipturus batis	North Atlantic High Sea	Elasmobranchii
Leucoraja naevus	North Atlantic High Sea	Elasmobranchii
Mobula tarapacana	North Atlantic High Sea	Elasmobranchii
Neoraja caerulea	North Atlantic High Sea	Elasmobranchii
Neoraja iberica	North Atlantic High Sea	Elasmobranchii
Rajella kukujevi	North Atlantic High Sea	Elasmobranchii
Acantholiparis opercularis	North East Pacific High Sea	Actinopterygii
Aldrovandia phalacra	North East Pacific High Sea	Actinopterygii
Allocyttus verrucosus	North East Pacific High Sea	Actinopterygii
Anguilla obscura	North East Pacific High Sea	Actinopterygii
Aphanopus arigato	North East Pacific High Sea	Actinopterygii
Barathronus diaphanus	North East Pacific High Sea	Actinopterygii
Barathronus pacificus	North East Pacific High Sea	Actinopterygii
Bassozetus galatheae	North East Pacific High Sea	Actinopterygii
Bassozetus glutinosus	North East Pacific High Sea	Actinopterygii
Bathygadus bowersi	North East Pacific High Sea	Actinopterygii

Rare Species	System	Class
Bathyuroconger vicinus	North East Pacific High Sea	Actinopterygii
Benthodesmus pacificus	North East Pacific High Sea	Actinopterygii
Benthosema panamense	North East Pacific High Sea	Actinopterygii
Bertella idiomorpha	North East Pacific High Sea	Actinopterygii
Beryx splendens	North East Pacific High Sea	Actinopterygii
Bothrocara molle	North East Pacific High Sea	Actinopterygii
Brama orcini	North East Pacific High Sea	Actinopterygii
Brama pauciradiata	North East Pacific High Sea	Actinopterygii
Champsodon longipinnis	North East Pacific High Sea	Actinopterygii
Cheilopogon nigricans	North East Pacific High Sea	Actinopterygii
Clidoderma asperrimum	North East Pacific High Sea	Actinopterygii
Coelorinchus kermadecus	North East Pacific High Sea	Actinopterygii
Congiopodus kieneri	North East Pacific High Sea	Actinopterygii
Conocara nigrum	North East Pacific High Sea	Actinopterygii
Coryphaenoides acrolepis	North East Pacific High Sea	Actinopterygii
Coryphaenoides anguliceps	North East Pacific High Sea	Actinopterygii
Coryphaenoides asprellus	North East Pacific High Sea	Actinopterygii
Coryphaenoides longicirrhus	North East Pacific High Sea	Actinopterygii
Diaphus impostor	North East Pacific High Sea	Actinopterygii
Diaphus regani	North East Pacific High Sea	Actinopterygii
Diaphus wisneri	North East Pacific High Sea	Actinopterygii
Dibranchus spinosus	North East Pacific High Sea	Actinopterygii
Dibranchus spongiosa	North East Pacific High Sea	Actinopterygii
Diretmoides pauciradiatus	North East Pacific High Sea	Actinopterygii
Encheliophis sagamianus	North East Pacific High Sea	Actinopterygii
Gephyroberyx darwinii	North East Pacific High Sea	Actinopterygii
Grammatonotus crosnieri	North East Pacific High Sea	Actinopterygii
Halieutopsis margaretae	North East Pacific High Sea	Actinopterygii
Holcomycteronus aequatoris	North East Pacific High Sea	Actinopterygii
Holcomycteronus profundissimus	North East Pacific High Sea	Actinopterygii
Hoplunnis sicarius	North East Pacific High Sea	Actinopterygii
Hygophum macrochir	North East Pacific High Sea	Actinopterygii
Hyperoglyphe japonica	North East Pacific High Sea	Actinopterygii
Lepidocybium flavobrunneum	North East Pacific High Sea	Actinopterygii
Leptobrotula breviventralis	North East Pacific High Sea	Actinopterygii
Leptostomias gracilis	North East Pacific High Sea	Actinopterygii
Luciobrotula bartschi	North East Pacific High Sea	Actinopterygii
Lycenchelys jordani	North East Pacific High Sea	Actinopterygii
Lycenchelys micropora	North East Pacific High Sea	Actinopterygii
Lycenchelys porifer	North East Pacific High Sea	Actinopterygii
Lycenchelys ratmanovi	North East Pacific High Sea	Actinopterygii
Lycenchelys ryukyuensis	North East Pacific High Sea	Actinopterygii
Merluccius productus	North East Pacific High Sea	Actinopterygii
Mirorictus taningi	North East Pacific High Sea	Actinopterygii
Nesiarchus nasutus	North East Pacific High Sea	Actinopterygii

**Rare Species**	**System**	**Class**
Nettastoma parviceps	North East Pacific High Sea	Actinopterygii
Nezumia burragei	North East Pacific High Sea	Actinopterygii
Nezumia obliquata	North East Pacific High Sea	Actinopterygii
Notacanthus abbotti	North East Pacific High Sea	Actinopterygii
Oneirodes melanocauda	North East Pacific High Sea	Actinopterygii
Pachycara gymninium	North East Pacific High Sea	Actinopterygii
Pachycara lepinium	North East Pacific High Sea	Actinopterygii
Paraliparis hawaiiensis	North East Pacific High Sea	Actinopterygii
Paraliparis meridionalis	North East Pacific High Sea	Actinopterygii
Parascombrops analis	North East Pacific High Sea	Actinopterygii
Pentaceros wheeleri	North East Pacific High Sea	Actinopterygii
Physiculus nematopus	North East Pacific High Sea	Actinopterygii
Polyipnus aquavitus	North East Pacific High Sea	Actinopterygii
Porogadus miles	North East Pacific High Sea	Actinopterygii
Psednos anoderkes	North East Pacific High Sea	Actinopterygii
Psednos cathetostomus	North East Pacific High Sea	Actinopterygii
Psednos griseus	North East Pacific High Sea	Actinopterygii
Psednos pallidus	North East Pacific High Sea	Actinopterygii
Psenopsis humerosa	North East Pacific High Sea	Actinopterygii
Psychrolutes phrictus	North East Pacific High Sea	Actinopterygii
Reinhardtius hippoglossoides	North East Pacific High Sea	Actinopterygii
Saccogaster tuberculata	North East Pacific High Sea	Actinopterygii
Scalicus hians	North East Pacific High Sea	Actinopterygii
Sebastolobus altivelis	North East Pacific High Sea	Actinopterygii
Sebastolobus macrochir	North East Pacific High Sea	Actinopterygii
Snyderidia canina	North East Pacific High Sea	Actinopterygii
Synagrops japonicus	North East Pacific High Sea	Actinopterygii
Synaphobranchus brevidorsalis	North East Pacific High Sea	Actinopterygii
Synaphobranchus kaupii	North East Pacific High Sea	Actinopterygii
Talismania bifurcata	North East Pacific High Sea	Actinopterygii
Venefica ocella	North East Pacific High Sea	Actinopterygii
Xenodermichthys nodulosus	North East Pacific High Sea	Actinopterygii
Xiphasia matsubarai	North East Pacific High Sea	Actinopterygii
Zenopsis nebulosa	North East Pacific High Sea	Actinopterygii
Bythaelurus giddingsi	North East Pacific High Sea	Elasmobranchii
Mobula eregoodootenkee	North East Pacific High Sea	Elasmobranchii
Acanthurus dussumieri	North West Pacific High Sea	Actinopterygii
Acanthurus leucopareius	North West Pacific High Sea	Actinopterygii
Acanthurus thompsoni	North West Pacific High Sea	Actinopterygii
Acanthurus triostegus	North West Pacific High Sea	Actinopterygii
Acanthurus xanthopterus	North West Pacific High Sea	Actinopterygii
Albatrossia pectoralis	North West Pacific High Sea	Actinopterygii
Amioides polyacanthus	North West Pacific High Sea	Actinopterygii
Anguilla marmorata	North West Pacific High Sea	Actinopterygii
Antennatus duescus	North West Pacific High Sea	Actinopterygii

Rare Species	System	Class
Aphareus rutilans	North West Pacific High Sea	Actinopterygii
Aprion virescens	North West Pacific High Sea	Actinopterygii
Argentina kagoshimae	North West Pacific High Sea	Actinopterygii
Auxis thazard	North West Pacific High Sea	Actinopterygii
Bassozetus elongatus	North West Pacific High Sea	Actinopterygii
Bodianus bilunulatus	North West Pacific High Sea	Actinopterygii
Bodianus paraleucosticticus	North West Pacific High Sea	Actinopterygii
Bodianus thoracotaeniatus	North West Pacific High Sea	Actinopterygii
Bolinia euryptera	North West Pacific High Sea	Actinopterygii
Bothrocara brunneum	North West Pacific High Sea	Actinopterygii
Bothrocara hollandi	North West Pacific High Sea	Actinopterygii
Brama dussumieri	North West Pacific High Sea	Actinopterygii
Cabillus macrophthalmus	North West Pacific High Sea	Actinopterygii
Canthigaster coronata	North West Pacific High Sea	Actinopterygii
Carangoides orthogrammus	North West Pacific High Sea	Actinopterygii
Caranx lugubris	North West Pacific High Sea	Actinopterygii
Caranx melampygus	North West Pacific High Sea	Actinopterygii
Carapus mourlani	North West Pacific High Sea	Actinopterygii
Cataetyx platyrhynchus	North West Pacific High Sea	Actinopterygii
Centropyge multicolor	North West Pacific High Sea	Actinopterygii
Cephalopholis igarashiensis	North West Pacific High Sea	Actinopterygii
Cephalopholis polleni	North West Pacific High Sea	Actinopterygii
Cephalopholis spiloparaea	North West Pacific High Sea	Actinopterygii
Chaetodon lineolatus	North West Pacific High Sea	Actinopterygii
Chaetodon lunula	North West Pacific High Sea	Actinopterygii
Chilorhinus platyrhynchus	North West Pacific High Sea	Actinopterygii
Chromis leucura	North West Pacific High Sea	Actinopterygii
Coelorinchus commutabilis	North West Pacific High Sea	Actinopterygii
Dysomma anguillare	North West Pacific High Sea	Actinopterygii
Encheliophis boraborensis	North West Pacific High Sea	Actinopterygii
Epinephelus chlorostigma	North West Pacific High Sea	Actinopterygii
Epinephelus magniscuttis	North West Pacific High Sea	Actinopterygii
Epinephelus miliaris	North West Pacific High Sea	Actinopterygii
Epinephelus morrhua	North West Pacific High Sea	Actinopterygii
Epinephelus radiatus	North West Pacific High Sea	Actinopterygii
Epinephelus retouti	North West Pacific High Sea	Actinopterygii
Epinephelus tauvina	North West Pacific High Sea	Actinopterygii
Etelis radiosus	North West Pacific High Sea	Actinopterygii
Evoxymetopon taeniatus	North West Pacific High Sea	Actinopterygii
Forcipiger flavissimus	North West Pacific High Sea	Actinopterygii
Forcipiger longirostris	North West Pacific High Sea	Actinopterygii
Fowlerichthys scriptissimus	North West Pacific High Sea	Actinopterygii
Genicanthus bellus	North West Pacific High Sea	Actinopterygii
Gracila albomarginata	North West Pacific High Sea	Actinopterygii
Gymnosarda unicolor	North West Pacific High Sea	Actinopterygii

**Rare Species**	**System**	**Class**
Gymnothorax berndti	North West Pacific High Sea	Actinopterygii
Gymnothorax flavimarginatus	North West Pacific High Sea	Actinopterygii
Gymnothorax nudivomer	North West Pacific High Sea	Actinopterygii
Hastatobythites arafurensis	North West Pacific High Sea	Actinopterygii
Helicolenus fedorovi	North West Pacific High Sea	Actinopterygii
Hemitaurichthys thompsoni	North West Pacific High Sea	Actinopterygii
Hoplobrotula armata	North West Pacific High Sea	Actinopterygii
Hoplolatilus marcosi	North West Pacific High Sea	Actinopterygii
Hyporthodus octofasciatus	North West Pacific High Sea	Actinopterygii
Iniistius baldwini	North West Pacific High Sea	Actinopterygii
Lagocephalus lagocephalus	North West Pacific High Sea	Actinopterygii
Lethrinus olivaceus	North West Pacific High Sea	Actinopterygii
Lethrinus xanthochilus	North West Pacific High Sea	Actinopterygii
Limnichthys fasciatus	North West Pacific High Sea	Actinopterygii
Lutjanus bohar	North West Pacific High Sea	Actinopterygii
Lutjanus gibbus	North West Pacific High Sea	Actinopterygii
Lutjanus kasmira	North West Pacific High Sea	Actinopterygii
Lycenchelys camchatica	North West Pacific High Sea	Actinopterygii
Lycodapus poecilus	North West Pacific High Sea	Actinopterygii
Melanostigma orientale	North West Pacific High Sea	Actinopterygii
Minyichthys brachyrhinus	North West Pacific High Sea	Actinopterygii
Myrichthys maculosus	North West Pacific High Sea	Actinopterygii
Myripristis vittata	North West Pacific High Sea	Actinopterygii
Nalbantichthys elongatus	North West Pacific High Sea	Actinopterygii
Naso hexacanthus	North West Pacific High Sea	Actinopterygii
Naso unicornis	North West Pacific High Sea	Actinopterygii
Neoniphon aurolineatus	North West Pacific High Sea	Actinopterygii
Ostichthys acanthorhinus	North West Pacific High Sea	Actinopterygii
Ostorhinchus gularis	North West Pacific High Sea	Actinopterygii
Oxycheilinus unifasciatus	North West Pacific High Sea	Actinopterygii
Paracaesio sordida	North West Pacific High Sea	Actinopterygii
Paracaesio xanthura	North West Pacific High Sea	Actinopterygii
Paracentropyge multifasciata	North West Pacific High Sea	Actinopterygii
Parupeneus multifasciatus	North West Pacific High Sea	Actinopterygii
Plectranthias winniensis	North West Pacific High Sea	Actinopterygii
Plectropomus pessuliferus	North West Pacific High Sea	Actinopterygii
Pogonoperca punctata	North West Pacific High Sea	Actinopterygii
Polyipnus paxtoni	North West Pacific High Sea	Actinopterygii
Polyipnus ruggeri	North West Pacific High Sea	Actinopterygii
Polylepion russelli	North West Pacific High Sea	Actinopterygii
Pristiapogon kallopterus	North West Pacific High Sea	Actinopterygii
Pristipomoides argyrogrammicus	North West Pacific High Sea	Actinopterygii
Pristipomoides flavipinnis	North West Pacific High Sea	Actinopterygii
Pristipomoides sieboldii	North West Pacific High Sea	Actinopterygii
Psammodiscus ocellatus	North West Pacific High Sea	Actinopterygii

**Rare Species**	**System**	**Class**
Pseudanthias ventralis	North West Pacific High Sea	Actinopterygii
Pteroidichthys acutus	North West Pacific High Sea	Actinopterygii
Pterygotrigla ryukyuensis	North West Pacific High Sea	Actinopterygii
Pycnocraspedum squamipinne	North West Pacific High Sea	Actinopterygii
Pyramodon lindas	North West Pacific High Sea	Actinopterygii
Randallichthys filamentosus	North West Pacific High Sea	Actinopterygii
Reinhardtius hippoglossoides	North West Pacific High Sea	Actinopterygii
Roa excelsa	North West Pacific High Sea	Actinopterygii
Schindleria praematura	North West Pacific High Sea	Actinopterygii
Scuticaria okinawae	North West Pacific High Sea	Actinopterygii
Sebastes iracundus	North West Pacific High Sea	Actinopterygii
Seriola dumerili	North West Pacific High Sea	Actinopterygii
Seriola lalandi	North West Pacific High Sea	Actinopterygii
Serranocirrhitus latus	North West Pacific High Sea	Actinopterygii
Sphoeroides pachygaster	North West Pacific High Sea	Actinopterygii
Sufflamen fraenatum	North West Pacific High Sea	Actinopterygii
Synodus kaianus	North West Pacific High Sea	Actinopterygii
Tauredophidium hextii	North West Pacific High Sea	Actinopterygii
Thamnaconus modestoides	North West Pacific High Sea	Actinopterygii
Thamnaconus striatus	North West Pacific High Sea	Actinopterygii
Thyrsitoides marleyi	North West Pacific High Sea	Actinopterygii
Triodon macropterus	North West Pacific High Sea	Actinopterygii
Variola albimarginata	North West Pacific High Sea	Actinopterygii
Variola louti	North West Pacific High Sea	Actinopterygii
Xanthichthys auromarginatus	North West Pacific High Sea	Actinopterygii
Xanthichthys caeruleolineatus	North West Pacific High Sea	Actinopterygii
Xanthichthys mento	North West Pacific High Sea	Actinopterygii
Zanclus cornutus	North West Pacific High Sea	Actinopterygii
Apristurus platyrhynchus	North West Pacific High Sea	Elasmobranchii
Carcharhinus amblyrhynchos	North West Pacific High Sea	Elasmobranchii
Carcharhinus galapagensis	North West Pacific High Sea	Elasmobranchii
Carcharhinus plumbeus	North West Pacific High Sea	Elasmobranchii
Galeocerdo cuvier	North West Pacific High Sea	Elasmobranchii
Galeus eastmani	North West Pacific High Sea	Elasmobranchii
Mobula eregoodootenkee	North West Pacific High Sea	Elasmobranchii
Rhinoraja longicauda	North West Pacific High Sea	Elasmobranchii
Aluterus heudelotii	South Atlantic High Sea	Actinopterygii
Amblyrhynchotes honckenii	South Atlantic High Sea	Actinopterygii
Ancylopsetta antillarum	South Atlantic High Sea	Actinopterygii
Aphanopus carbo	South Atlantic High Sea	Actinopterygii
Apterichtus monodi	South Atlantic High Sea	Actinopterygii
Argyrosomus regius	South Atlantic High Sea	Actinopterygii
Ariosoma balearicum	South Atlantic High Sea	Actinopterygii
Ariosoma selenops	South Atlantic High Sea	Actinopterygii
Arnoglossus capensis	South Atlantic High Sea	Actinopterygii

**Rare Species**	**System**	**Class**
Arnoglossus imperialis	South Atlantic High Sea	Actinopterygii
Arothron firmamentum	South Atlantic High Sea	Actinopterygii
Assurger anzac	South Atlantic High Sea	Actinopterygii
Atractoscion aequidens	South Atlantic High Sea	Actinopterygii
Auxis thazard	South Atlantic High Sea	Actinopterygii
Balistes capriscus	South Atlantic High Sea	Actinopterygii
Barathrodemus manatinus	South Atlantic High Sea	Actinopterygii
Barathronus bicolor	South Atlantic High Sea	Actinopterygii
Bassozetus levistomatus	South Atlantic High Sea	Actinopterygii
Bathyuroconger vicinus	South Atlantic High Sea	Actinopterygii
Boops boops	South Atlantic High Sea	Actinopterygii
Bothus podas	South Atlantic High Sea	Actinopterygii
Bregmaceros nectabanus	South Atlantic High Sea	Actinopterygii
Brotula barbata	South Atlantic High Sea	Actinopterygii
Butis melanostigma	South Atlantic High Sea	Actinopterygii
Caranx hippos	South Atlantic High Sea	Actinopterygii
Careproctus improvisus	South Atlantic High Sea	Actinopterygii
Cataetyx messieri	South Atlantic High Sea	Actinopterygii
Chelidonichthys lastoviza	South Atlantic High Sea	Actinopterygii
Chionodraco hamatus	South Atlantic High Sea	Actinopterygii
Coloconger eximia	South Atlantic High Sea	Actinopterygii
Corniger spinosus	South Atlantic High Sea	Actinopterygii
Cottunculus granulosus	South Atlantic High Sea	Actinopterygii
Dactylopterus volitans	South Atlantic High Sea	Actinopterygii
Decapterus macarellus	South Atlantic High Sea	Actinopterygii
Decapterus punctatus	South Atlantic High Sea	Actinopterygii
Decapterus tabl	South Atlantic High Sea	Actinopterygii
Dentex gibbosus	South Atlantic High Sea	Actinopterygii
Dentex macrophthalmus	South Atlantic High Sea	Actinopterygii
Dibranchus tremendus	South Atlantic High Sea	Actinopterygii
Dicrolene kanazawai	South Atlantic High Sea	Actinopterygii
Diplacanthopoma brachysoma	South Atlantic High Sea	Actinopterygii
Diplodus cervinus	South Atlantic High Sea	Actinopterygii
Diplodus sargus	South Atlantic High Sea	Actinopterygii
Echiodon cryomargarites	South Atlantic High Sea	Actinopterygii
Engraulis encrasicolus	South Atlantic High Sea	Actinopterygii
Epinephelus caninus	South Atlantic High Sea	Actinopterygii
Epinephelus costae	South Atlantic High Sea	Actinopterygii
Epinephelus marginatus	South Atlantic High Sea	Actinopterygii
Erythrocles monodi	South Atlantic High Sea	Actinopterygii
Exechodontes daidaleus	South Atlantic High Sea	Actinopterygii
Fodiator acutus	South Atlantic High Sea	Actinopterygii
Genypterus blacodes	South Atlantic High Sea	Actinopterygii
Gephyroberyx darwinii	South Atlantic High Sea	Actinopterygii
Gymnothorax maderensis	South Atlantic High Sea	Actinopterygii

Rare Species	System	Class
Gymnothorax polygonius	South Atlantic High Sea	Actinopterygii
Histiopterus typus	South Atlantic High Sea	Actinopterygii
Hollardia hollardi	South Atlantic High Sea	Actinopterygii
Hyporthodus haifensis	South Atlantic High Sea	Actinopterygii
Kajikia audax	South Atlantic High Sea	Actinopterygii
Lagocephalus inermis	South Atlantic High Sea	Actinopterygii
Lagocephalus lagocephalus	South Atlantic High Sea	Actinopterygii
Lamprogrammus exutus	South Atlantic High Sea	Actinopterygii
Lamprogrammus shcherbachevi	South Atlantic High Sea	Actinopterygii
Lepidonotothen squamifrons	South Atlantic High Sea	Actinopterygii
Lepidopus caudatus	South Atlantic High Sea	Actinopterygii
Lepidotrigla cadmani	South Atlantic High Sea	Actinopterygii
Letholycus microphthalmus	South Atlantic High Sea	Actinopterygii
Lophiomus setigerus	South Atlantic High Sea	Actinopterygii
Luciobrotula corethromycter	South Atlantic High Sea	Actinopterygii
Lycenchelys paxillus	South Atlantic High Sea	Actinopterygii
Macrorhamphosodes uradoi	South Atlantic High Sea	Actinopterygii
Microchirus boscanion	South Atlantic High Sea	Actinopterygii
Micromesistius australis	South Atlantic High Sea	Actinopterygii
Mixomyrophis pusillipinna	South Atlantic High Sea	Actinopterygii
Monocentris japonica	South Atlantic High Sea	Actinopterygii
Monomitopus agassizii	South Atlantic High Sea	Actinopterygii
Monomitopus americanus	South Atlantic High Sea	Actinopterygii
Naucrates ductor	South Atlantic High Sea	Actinopterygii
Nemoossis belloci	South Atlantic High Sea	Actinopterygii
Neobythites braziliensis	South Atlantic High Sea	Actinopterygii
Neobythites elongatus	South Atlantic High Sea	Actinopterygii
Notolycodes schmidti	South Atlantic High Sea	Actinopterygii
Notothenia rossii	South Atlantic High Sea	Actinopterygii
Oidiphorus brevis	South Atlantic High Sea	Actinopterygii
Ophidion puck	South Atlantic High Sea	Actinopterygii
Ophisurus serpens	South Atlantic High Sea	Actinopterygii
Ophthalmolycus amberensis	South Atlantic High Sea	Actinopterygii
Pachycara brachycephalum	South Atlantic High Sea	Actinopterygii
Pachycara caribbaeum	South Atlantic High Sea	Actinopterygii
Pachycara crassiceps	South Atlantic High Sea	Actinopterygii
Pagrus pagrus	South Atlantic High Sea	Actinopterygii
Parabathymyrus oregoni	South Atlantic High Sea	Actinopterygii
Pentaceros capensis	South Atlantic High Sea	Actinopterygii
Peristedion cataphractum	South Atlantic High Sea	Actinopterygii
Peristedion longispatha	South Atlantic High Sea	Actinopterygii
Plesienchelys stehmanni	South Atlantic High Sea	Actinopterygii
Pogonolycus elegans	South Atlantic High Sea	Actinopterygii
Pogonophryne marmorata	South Atlantic High Sea	Actinopterygii
Polyipnus clarus	South Atlantic High Sea	Actinopterygii

**Rare Species**	**System**	**Class**
Polyipnus laternatus	South Atlantic High Sea	Actinopterygii
Polyipnus polli	South Atlantic High Sea	Actinopterygii
Prognichthys sealei	South Atlantic High Sea	Actinopterygii
Rachycentron canadum	South Atlantic High Sea	Actinopterygii
Salilota australis	South Atlantic High Sea	Actinopterygii
Sarda sarda	South Atlantic High Sea	Actinopterygii
Sardinella aurita	South Atlantic High Sea	Actinopterygii
Sardinops sagax	South Atlantic High Sea	Actinopterygii
Saurida brasiliensis	South Atlantic High Sea	Actinopterygii
Schedophilus griseolineatus	South Atlantic High Sea	Actinopterygii
Scomber colias	South Atlantic High Sea	Actinopterygii
Scorpaena scrofa	South Atlantic High Sea	Actinopterygii
Seleniolycus laevifasciatus	South Atlantic High Sea	Actinopterygii
Seriola lalandi	South Atlantic High Sea	Actinopterygii
Sonoda megalophthalma	South Atlantic High Sea	Actinopterygii
Stephanolepis hispidus	South Atlantic High Sea	Actinopterygii
Symphodus bailloni	South Atlantic High Sea	Actinopterygii
Taractes rubescens	South Atlantic High Sea	Actinopterygii
Thyrsites atun	South Atlantic High Sea	Actinopterygii
Trachinocephalus myops	South Atlantic High Sea	Actinopterygii
Trachinotus ovatus	South Atlantic High Sea	Actinopterygii
Trachinus radiatus	South Atlantic High Sea	Actinopterygii
Trachurus picturatus	South Atlantic High Sea	Actinopterygii
Trematomus eulepidotus	South Atlantic High Sea	Actinopterygii
Trematomus hansoni	South Atlantic High Sea	Actinopterygii
Trematomus loennbergii	South Atlantic High Sea	Actinopterygii
Trematomus scotti	South Atlantic High Sea	Actinopterygii
Trematomus tokarevi	South Atlantic High Sea	Actinopterygii
Trigla lyra	South Atlantic High Sea	Actinopterygii
Tylerius spinosissimus	South Atlantic High Sea	Actinopterygii
Umbrina canariensis	South Atlantic High Sea	Actinopterygii
Umbrina ronchus	South Atlantic High Sea	Actinopterygii
Xenomystax bidentatus	South Atlantic High Sea	Actinopterygii
Zenopsis conchifer	South Atlantic High Sea	Actinopterygii
Bathyraja macloviana	South Atlantic High Sea	Elasmobranchii
Bathyraja magellanica	South Atlantic High Sea	Elasmobranchii
Bathyraja multispinis	South Atlantic High Sea	Elasmobranchii
Bathyraja schroederi	South Atlantic High Sea	Elasmobranchii
Breviraja colesi	South Atlantic High Sea	Elasmobranchii
Carcharhinus galapagensis	South Atlantic High Sea	Elasmobranchii
Cruriraja rugosa	South Atlantic High Sea	Elasmobranchii
Dactylobatus clarkii	South Atlantic High Sea	Elasmobranchii
Fenestraja cubensis	South Atlantic High Sea	Elasmobranchii
Fenestraja ishiyamai	South Atlantic High Sea	Elasmobranchii
Galeocerdo cuvier	South Atlantic High Sea	Elasmobranchii

Rare Species	System	Class
Raja bahamensis	South Atlantic High Sea	Elasmobranchii
Rajella fuliginea	South Atlantic High Sea	Elasmobranchii
Acanthochaenus luetkenii	South East Pacific High Sea	Actinopterygii
Anampses elegans	South East Pacific High Sea	Actinopterygii
Anguilla megastoma	South East Pacific High Sea	Actinopterygii
Anguilla obscura	South East Pacific High Sea	Actinopterygii
Argyripnus electronus	South East Pacific High Sea	Actinopterygii
Argyripnus ephippiatus	South East Pacific High Sea	Actinopterygii
Aseraggodes bahamondei	South East Pacific High Sea	Actinopterygii
Assurger anzac	South East Pacific High Sea	Actinopterygii
Astroscopus zephyreus	South East Pacific High Sea	Actinopterygii
Auxis rochei	South East Pacific High Sea	Actinopterygii
Barathronus diaphanus	South East Pacific High Sea	Actinopterygii
Bathyuroconger vicinus	South East Pacific High Sea	Actinopterygii
Bodianus unimaculatus	South East Pacific High Sea	Actinopterygii
Bollmannia stigmatura	South East Pacific High Sea	Actinopterygii
Bothrocara molle	South East Pacific High Sea	Actinopterygii
Canthigaster inframacula	South East Pacific High Sea	Actinopterygii
Caprodon longimanus	South East Pacific High Sea	Actinopterygii
Caprodon schlegelii	South East Pacific High Sea	Actinopterygii
Centrodraco atrifilum	South East Pacific High Sea	Actinopterygii
Centropyge interrupta	South East Pacific High Sea	Actinopterygii
Chaenopsis alepidota	South East Pacific High Sea	Actinopterygii
Chaetodon guentheri	South East Pacific High Sea	Actinopterygii
Chaunax latipunctatus	South East Pacific High Sea	Actinopterygii
Chaunax penicillatus	South East Pacific High Sea	Actinopterygii
Cheilodactylus gibbosus	South East Pacific High Sea	Actinopterygii
Cheilodactylus vittatus	South East Pacific High Sea	Actinopterygii
Congiopodus coriaceus	South East Pacific High Sea	Actinopterygii
Congiopodus kieneri	South East Pacific High Sea	Actinopterygii
Cosmocampus arctus	South East Pacific High Sea	Actinopterygii
Cottunculus nudus	South East Pacific High Sea	Actinopterygii
Decapterus tabl	South East Pacific High Sea	Actinopterygii
Diaphus kora	South East Pacific High Sea	Actinopterygii
Dibranchus hystrix	South East Pacific High Sea	Actinopterygii
Dicrolene longimana	South East Pacific High Sea	Actinopterygii
Dicrolene nigra	South East Pacific High Sea	Actinopterygii
Diplacanthopoma jordani	South East Pacific High Sea	Actinopterygii
Engyprosopon arenicola	South East Pacific High Sea	Actinopterygii
Enneapterygius rufopileus	South East Pacific High Sea	Actinopterygii
Festucalex erythraeus	South East Pacific High Sea	Actinopterygii
Gadomus aoteanus	South East Pacific High Sea	Actinopterygii
Glyptophidium lucidum	South East Pacific High Sea	Actinopterygii
Gymnothorax australicola	South East Pacific High Sea	Actinopterygii
Gymnothorax berndti	South East Pacific High Sea	Actinopterygii

**Rare Species**	**System**	**Class**
Gymnothorax kidako	South East Pacific High Sea	Actinopterygii
Gymnothorax porphyreus	South East Pacific High Sea	Actinopterygii
Gymnothorax ypsilon	South East Pacific High Sea	Actinopterygii
Halicampus boothae	South East Pacific High Sea	Actinopterygii
Helicolenus avius	South East Pacific High Sea	Actinopterygii
Heteroclinus roseus	South East Pacific High Sea	Actinopterygii
Hime japonica	South East Pacific High Sea	Actinopterygii
Histiophryne cryptacanthus	South East Pacific High Sea	Actinopterygii
Holcomycteronus digittatus	South East Pacific High Sea	Actinopterygii
Homostolus acer	South East Pacific High Sea	Actinopterygii
Hypopleuron caninum	South East Pacific High Sea	Actinopterygii
Hyporhamphus rosae	South East Pacific High Sea	Actinopterygii
Hypsoblennius gentilis	South East Pacific High Sea	Actinopterygii
Hypsoblennius jenkinsi	South East Pacific High Sea	Actinopterygii
Ilypnus gilberti	South East Pacific High Sea	Actinopterygii
Itycirrhitus wilhelmi	South East Pacific High Sea	Actinopterygii
Kentrocapros aculeatus	South East Pacific High Sea	Actinopterygii
Kentrocapros flavofasciatus	South East Pacific High Sea	Actinopterygii
Labrisomus xanti	South East Pacific High Sea	Actinopterygii
Lagocephalus lagocephalus	South East Pacific High Sea	Actinopterygii
Lamprogrammus shcherbachevi	South East Pacific High Sea	Actinopterygii
Leptonotus blainvilleanus	South East Pacific High Sea	Actinopterygii
Limnichthys fasciatus	South East Pacific High Sea	Actinopterygii
Lumpenopsis hypochroma	South East Pacific High Sea	Actinopterygii
Lycenchelys pearcyi	South East Pacific High Sea	Actinopterygii
Lycenchelys porifer	South East Pacific High Sea	Actinopterygii
Lythrypnus dalli	South East Pacific High Sea	Actinopterygii
Lythrypnus zebra	South East Pacific High Sea	Actinopterygii
Macrorhamphosodes uradoi	South East Pacific High Sea	Actinopterygii
Malthopsis parva	South East Pacific High Sea	Actinopterygii
Matanui bathytaton	South East Pacific High Sea	Actinopterygii
Metavelifer multiradiatus	South East Pacific High Sea	Actinopterygii
Microcanthus strigatus	South East Pacific High Sea	Actinopterygii
Micromesistius australis	South East Pacific High Sea	Actinopterygii
Mugil cephalus	South East Pacific High Sea	Actinopterygii
Naucrates ductor	South East Pacific High Sea	Actinopterygii
Neobythites fasciatus	South East Pacific High Sea	Actinopterygii
Neobythites pallidus	South East Pacific High Sea	Actinopterygii
Nototheniops nybelini	South East Pacific High Sea	Actinopterygii
Novaculops koteamea	South East Pacific High Sea	Actinopterygii
Ophichthus triserialis	South East Pacific High Sea	Actinopterygii
Oplegnathus punctatus	South East Pacific High Sea	Actinopterygii
Pachycara brachycephalum	South East Pacific High Sea	Actinopterygii
Pachycara suspectum	South East Pacific High Sea	Actinopterygii
Paralichthys patagonicus	South East Pacific High Sea	Actinopterygii

**Rare Species**	**System**	**Class**
Paraliparis cephalus	South East Pacific High Sea	Actinopterygii
Paraliparis meridionalis	South East Pacific High Sea	Actinopterygii
Parupeneus spilurus	South East Pacific High Sea	Actinopterygii
Patagonotothen cornucola	South East Pacific High Sea	Actinopterygii
Patagonotothen sima	South East Pacific High Sea	Actinopterygii
Pentaceros capensis	South East Pacific High Sea	Actinopterygii
Pentaceros quinquespinis	South East Pacific High Sea	Actinopterygii
Pherallodus indicus	South East Pacific High Sea	Actinopterygii
Plectranthias yamakawai	South East Pacific High Sea	Actinopterygii
Polyipnus matsubarai	South East Pacific High Sea	Actinopterygii
Polyipnus triphanos	South East Pacific High Sea	Actinopterygii
Polylepion russelli	South East Pacific High Sea	Actinopterygii
Priacanthus nasca	South East Pacific High Sea	Actinopterygii
Prionotus stephanophrys	South East Pacific High Sea	Actinopterygii
Pristilepis oligolepis	South East Pacific High Sea	Actinopterygii
Prognathodes falcifer	South East Pacific High Sea	Actinopterygii
Promethichthys prometheus	South East Pacific High Sea	Actinopterygii
Psednos cathetostomus	South East Pacific High Sea	Actinopterygii
Pseudocaranx dentex	South East Pacific High Sea	Actinopterygii
Pseudonus squamiceps	South East Pacific High Sea	Actinopterygii
Pteraclis aesticola	South East Pacific High Sea	Actinopterygii
Pterygotrigla picta	South East Pacific High Sea	Actinopterygii
Sardinops sagax	South East Pacific High Sea	Actinopterygii
Sargocentron spinosissimum	South East Pacific High Sea	Actinopterygii
Saurenchelys stylura	South East Pacific High Sea	Actinopterygii
Scalicus engyceros	South East Pacific High Sea	Actinopterygii
Scomber japonicus	South East Pacific High Sea	Actinopterygii
Scorpaenodes evides	South East Pacific High Sea	Actinopterygii
Scuticaria okinawae	South East Pacific High Sea	Actinopterygii
Seriola dumerili	South East Pacific High Sea	Actinopterygii
Sphoeroides lispus	South East Pacific High Sea	Actinopterygii
Sphoeroides pachygaster	South East Pacific High Sea	Actinopterygii
Syngnathus auliscus	South East Pacific High Sea	Actinopterygii
Synodus doaki	South East Pacific High Sea	Actinopterygii
Taractes rubescens	South East Pacific High Sea	Actinopterygii
Tauredophidium hextii	South East Pacific High Sea	Actinopterygii
Trachinotus paitensis	South East Pacific High Sea	Actinopterygii
Trachurus murphyi	South East Pacific High Sea	Actinopterygii
Trichiurus lepturus	South East Pacific High Sea	Actinopterygii
Tylosurus pacificus	South East Pacific High Sea	Actinopterygii
Xiphasia matsubarai	South East Pacific High Sea	Actinopterygii
Bathyraja abyssicola	South East Pacific High Sea	Elasmobranchii
Bathyraja shuntovi	South East Pacific High Sea	Elasmobranchii
Carcharhinus brachyurus	South East Pacific High Sea	Elasmobranchii
Galeocerdo cuvier	South East Pacific High Sea	Elasmobranchii

**Rare Species**	**System**	**Class**
Sinobatis borneensis	South East Pacific High Sea	Elasmobranchii
Tetronarce microdiscus	South East Pacific High Sea	Elasmobranchii
Anguilla australis	South West Pacific High Sea	Actinopterygii
Anguilla reinhardtii	South West Pacific High Sea	Actinopterygii
Antipodocottus galatheae	South West Pacific High Sea	Actinopterygii
Aphanopus capricornis	South West Pacific High Sea	Actinopterygii
Auxis thazard	South West Pacific High Sea	Actinopterygii
Azygopus pinnifasciatus	South West Pacific High Sea	Actinopterygii
Barathronus maculatus	South West Pacific High Sea	Actinopterygii
Bathysaurus mollis	South West Pacific High Sea	Actinopterygii
Bathyuroconger vicinus	South West Pacific High Sea	Actinopterygii
Benthodesmus elongatus	South West Pacific High Sea	Actinopterygii
Benthodesmus tenuis	South West Pacific High Sea	Actinopterygii
Benthodesmus tuckeri	South West Pacific High Sea	Actinopterygii
Beryx decadactylus	South West Pacific High Sea	Actinopterygii
Bodianus cylindriatus	South West Pacific High Sea	Actinopterygii
Brotulotaenia nielseni	South West Pacific High Sea	Actinopterygii
Careproctus novaezelandiae	South West Pacific High Sea	Actinopterygii
Caristius meridionalis	South West Pacific High Sea	Actinopterygii
Centrodraco atrifilum	South West Pacific High Sea	Actinopterygii
Chaunacops coloratus	South West Pacific High Sea	Actinopterygii
Chaunax penicillatus	South West Pacific High Sea	Actinopterygii
Cheilopogon atrisignis	South West Pacific High Sea	Actinopterygii
Cheilopogon nigricans	South West Pacific High Sea	Actinopterygii
Cheilopogon spilonotopterus	South West Pacific High Sea	Actinopterygii
Chiasmodon subniger	South West Pacific High Sea	Actinopterygii
Coelophrys oblonga	South West Pacific High Sea	Actinopterygii
Conocara nigrum	South West Pacific High Sea	Actinopterygii
Cubiceps capensis	South West Pacific High Sea	Actinopterygii
Cypselurus naresii	South West Pacific High Sea	Actinopterygii
Cyttomimus stelgis	South West Pacific High Sea	Actinopterygii
Cyttus traversi	South West Pacific High Sea	Actinopterygii
Diaphus chrysorhynchus	South West Pacific High Sea	Actinopterygii
Diaphus impostor	South West Pacific High Sea	Actinopterygii
Diaphus regani	South West Pacific High Sea	Actinopterygii
Dicrolene tristis	South West Pacific High Sea	Actinopterygii
Diplospinus multistriatus	South West Pacific High Sea	Actinopterygii
Dissostichus eleginoides	South West Pacific High Sea	Actinopterygii
Dysommina rugosa	South West Pacific High Sea	Actinopterygii
Eustomias vulgaris	South West Pacific High Sea	Actinopterygii
Gadomus aoteanus	South West Pacific High Sea	Actinopterygii
Genypterus blacodes	South West Pacific High Sea	Actinopterygii
Gephyroberyx darwinii	South West Pacific High Sea	Actinopterygii
Glyptophidium lucidum	South West Pacific High Sea	Actinopterygii
Halicmetus reticulatus	South West Pacific High Sea	Actinopterygii

**Rare Species**	**System**	**Class**
Halicmetus ruber	South West Pacific High Sea	Actinopterygii
Halieutopsis margaretae	South West Pacific High Sea	Actinopterygii
Halimochirurgus alcocki	South West Pacific High Sea	Actinopterygii
Helicolenus avius	South West Pacific High Sea	Actinopterygii
Helicolenus barathri	South West Pacific High Sea	Actinopterygii
Helicolenus percoides	South West Pacific High Sea	Actinopterygii
Homostolus acer	South West Pacific High Sea	Actinopterygii
Hoplichthys haswelli	South West Pacific High Sea	Actinopterygii
Hoplostethus melanopeza	South West Pacific High Sea	Actinopterygii
Hymenocephalus antraeus	South West Pacific High Sea	Actinopterygii
Ichthyococcus intermedius	South West Pacific High Sea	Actinopterygii
Leptobrotula breviventralis	South West Pacific High Sea	Actinopterygii
Lophiodes endoi	South West Pacific High Sea	Actinopterygii
Lycodapus endemoscotus	South West Pacific High Sea	Actinopterygii
Macroramphosus scolopax	South West Pacific High Sea	Actinopterygii
Malthopsis gigas	South West Pacific High Sea	Actinopterygii
Malthopsis parva	South West Pacific High Sea	Actinopterygii
Monodactylus argenteus	South West Pacific High Sea	Actinopterygii
Myctophum lunatum	South West Pacific High Sea	Actinopterygii
Neobythites fasciatus	South West Pacific High Sea	Actinopterygii
Ophichthus tomioi	South West Pacific High Sea	Actinopterygii
Ostichthys sandix	South West Pacific High Sea	Actinopterygii
Oxyporhamphus micropterus	South West Pacific High Sea	Actinopterygii
Pachycara brachycephalum	South West Pacific High Sea	Actinopterygii
Pachycara suspectum	South West Pacific High Sea	Actinopterygii
Paraliparis cephalus	South West Pacific High Sea	Actinopterygii
Parexocoetus mento	South West Pacific High Sea	Actinopterygii
Pentaceros richardsoni	South West Pacific High Sea	Actinopterygii
Peristedion longicornutum	South West Pacific High Sea	Actinopterygii
Polyipnus aquavitus	South West Pacific High Sea	Actinopterygii
Polyprion oxygeneios	South West Pacific High Sea	Actinopterygii
Promethichthys prometheus	South West Pacific High Sea	Actinopterygii
Psenes arafurensis	South West Pacific High Sea	Actinopterygii
Pseudonus squamiceps	South West Pacific High Sea	Actinopterygii
Pterygotrigla andertoni	South West Pacific High Sea	Actinopterygii
Pterygotrigla macrorhynchus	South West Pacific High Sea	Actinopterygii
Pterygotrigla megalops	South West Pacific High Sea	Actinopterygii
Pyramodon punctatus	South West Pacific High Sea	Actinopterygii
Rexea solandri	South West Pacific High Sea	Actinopterygii
Ruvettus pretiosus	South West Pacific High Sea	Actinopterygii
Saccopharynx schmidti	South West Pacific High Sea	Actinopterygii
Seriola lalandi	South West Pacific High Sea	Actinopterygii
Solocisquama stellulata	South West Pacific High Sea	Actinopterygii
Symphurus thermophilus	South West Pacific High Sea	Actinopterygii
Symphysanodon maunaloae	South West Pacific High Sea	Actinopterygii

**Rare Species**	**System**	**Class**
Talismania bifurcata	South West Pacific High Sea	Actinopterygii
Tetragonurus pacificus	South West Pacific High Sea	Actinopterygii
Thamnaconus tessellatus	South West Pacific High Sea	Actinopterygii
Thyrsites atun	South West Pacific High Sea	Actinopterygii
Trachurus murphyi	South West Pacific High Sea	Actinopterygii
Trichiurus lepturus	South West Pacific High Sea	Actinopterygii
Valenciennellus carlsbergi	South West Pacific High Sea	Actinopterygii
Zenopsis nebulosa	South West Pacific High Sea	Actinopterygii
Asymbolus galacticus	South West Pacific High Sea	Elasmobranchii
Bathyraja andriashevi	South West Pacific High Sea	Elasmobranchii
Bathyraja shuntovi	South West Pacific High Sea	Elasmobranchii
Bathyraja spinosissima	South West Pacific High Sea	Elasmobranchii
Bathyraja tzinovskii	South West Pacific High Sea	Elasmobranchii
Carcharhinus longimanus	South West Pacific High Sea	Elasmobranchii
Notoraja sapphira	South West Pacific High Sea	Elasmobranchii
Pteroplatytrygon violacea	South West Pacific High Sea	Elasmobranchii
