# Supplementary material for: Global patterns in functional rarity of marine fish
Source: Nat Commun. 2022 Feb 15;13:877. doi: 10.1038/s41467-022-28488-1 (PMC8847455; doi:10.1038/s41467-022-28488-1)
Supplement: Supplementary file 3 — Reporting Summary [file 41467_2022_28488_MOESM3_ESM.pdf]

nature portfolio

Corresponding author(s): Isaac Trindade-Santos

Last updated by author(s): 26 Jan 2022

Reporting Summary

Nature Portfolio wishes to improve the reproducibility of the work that we publish. This form provides structure for consistency and transparency in reporting. For further information on Nature Portfolio policies, see our Editorial Policies and the Editorial Policy Checklist.

Statistics

For all statistical analyses, confirm that the following items are present in the figure legend, table legend, main text, or Methods section.

n/a	Confirmed	
☐	☒	The exact sample size (*n*) for each experimental group/condition, given as a discrete number and unit of measurement
☐	☒	A statement on whether measurements were taken from distinct samples or whether the same sample was measured repeatedly
☐	☒	The statistical test(s) used AND whether they are one- or two-sided<br>*Only common tests should be described solely by name; describe more complex techniques in the Methods section.*
☐	☒	A description of all covariates tested
☐	☒	A description of any assumptions or corrections, such as tests of normality and adjustment for multiple comparisons
☐	☒	A full description of the statistical parameters including central tendency (e.g. means) or other basic estimates (e.g. regression coefficient) AND variation (e.g. standard deviation) or associated estimates of uncertainty (e.g. confidence intervals)
☐	☒	For null hypothesis testing, the test statistic (e.g. *F*, *t*, *r*) with confidence intervals, effect sizes, degrees of freedom and *P* value noted<br>*Give P values as exact values whenever suitable.*
☒	☐	For Bayesian analysis, information on the choice of priors and Markov chain Monte Carlo settings
☒	☐	For hierarchical and complex designs, identification of the appropriate level for tests and full reporting of outcomes
☐	☒	Estimates of effect sizes (e.g. Cohen's *d*, Pearson's *r*), indicating how they were calculated

Our web collection on statistics for biologists contains articles on many of the points above.

Software and code

Policy information about availability of computer code

Data collection	All data used in this study was collected directly from the FishBase (Nicolas Bailly: n.bailly@q-quatics.org) and AquaMaps (Cristina Garilao: cgarilao@geomar.de) teams. No software was used to acquire/collect the data. Distribution data was compiled and assembled from AquaMaps (https://www.aquamaps.org/), trait data was compiled and assembled from FishBase (https://www.fishbase.in/search.php).
Data analysis	The following R packages were used to run the analysis: "funrar" (version 1.4.1), "tidyverse" (1.3.1), "missForest" (1.4), "stats" (4.1.0), "DescTools" (0.99.42). The "curveball" algorithm developed by Strona etal., 2014 (https://doi.org/10.1038/ncomms5114) was applied using Python. The software ArcGIS version 10.7 was used to produce the global maps.

For manuscripts utilizing custom algorithms or software that are central to the research but not yet described in published literature, software must be made available to editors and reviewers. We strongly encourage code deposition in a community repository (e.g. GitHub). See the Nature Portfolio guidelines for submitting code & software for further information.

Data

Policy information about availability of data

All manuscripts must include a data availability statement. This statement should provide the following information, where applicable:

- Accession codes, unique identifiers, or web links for publicly available datasets
- A description of any restrictions on data availability
- For clinical datasets or third party data, please ensure that the statement adheres to our policy

All data compiled and assembled can be accessed at the following link: https://doi.org/10.17630/397bc872-f7de-4ded-9ed8-4f734c11b14a. Trait data was acquired from FishBase and distribution data was acquired from AquaMaps.

Field-specific reporting

Please select the one below that is the best fit for your research. If you are not sure, read the appropriate sections before making your selection.

☐ Life sciences ☐ Behavioural & social sciences ☒ Ecological, evolutionary & environmental sciences

For a reference copy of the document with all sections, see nature.com/documents/nr-reporting-summary-flat.pdf

Ecological, evolutionary & environmental sciences study design

All studies must disclose on these points even when the disclosure is negative.

Study description	Here we adopt an integrated approach, combining information on the rarity of species trait combinations, and their spatial restrictedness, to quantify the biogeography of rare fish (a taxon with almost 13,000 species) in the world`s oceans.
Research sample	We used distribution data for all marine fish species presented in AquaMaps. This database provides occurrence data at the half degree grid cells. We first assigned each half degree grid cell into two degree grid cells in order to save computational power due to the high number of half degree grid cells. Second, we classified the two degree grid cells as seven coastal systems and seven high seas systems. Therefore, the "research sample" used here is composed by all described bony fishes (11,961 species) and cartilaginous fishes (866).
Sampling strategy	AquaMaps provides distribution data based on different probabilities of occurrences. We decided to run all the analysis using three different probabilities. Those are: probabilities higher than 0.9, higher than 0.7 and higher than 0.5. This decision
Data collection	Data was complied and assembled from all existing information from AquaMaps and FishBase.
Timing and spatial scale	No temporal scale was used in this study. The analysis was done at global spatial scale, encompassing all oceanic regions of the world.
Data exclusions	The AquaMaps online database provides species occurrences based on data from GBIF and OBIS complemented with information from FishBase and SeaLifeBase. It also gives probabilities of the occurrence of a given species between 0 and 1 (this is better explained at the methods section). In our analysis we selected occurrence data (the presence of a given species in a certain grid cell) with a probability higher than 0.9, 0.7 and 0.5. We repeated all the analysis for each of those probabilities to show the robustness of our approach.
Reproducibility	We are providing the code and data to ensure the reproducibility of our framework. All analysis was replicated for each of those independent systems: 2 indices (distinctivness and uniqueness), 2 classes of fish (bony and cartilaginous fish), 14 oceanic regions (coastal and high sea systems), 3 probabilities of occurrences (0.9, 0.7 and 0.5).
Randomization	Randomization was part of the null model applied, with 2000 randomized loops to generate the null expectation. Following the curve ball algorithm.
Blinding	Blinding was not relevant for this study because this is a macroecological analysis applied using all distribution data available for all described fish in AquaMaps.

Did the study involve field work? ☐ Yes ☒ No

Reporting for specific materials, systems and methods

We require information from authors about some types of materials, experimental systems and methods used in many studies. Here, indicate whether each material, system or method listed is relevant to your study. If you are not sure if a list item applies to your research, read the appropriate section before selecting a response.

Materials & experimental systems

n/a	Involved in the study	
☒	☐	Antibodies
☒	☐	Eukaryotic cell lines
☒	☐	Palaeontology and archaeology
☒	☐	Animals and other organisms
☒	☐	Human research participants
☒	☐	Clinical data
☒	☐	Dual use research of concern

Methods

n/a	Involved in the study	
☒	☐	ChIP-seq
☒	☐	Flow cytometry
☒	☐	MRI-based neuroimaging
